# A theoretical study of the radical scavenging activity of natural stilbenes†

**DOI:** 10.1039/c9ra08381b

**Published:** 2019-12-18

**Authors:** Quan V. Vo, Pham Cam Nam, Mai Van Bay, Nguyen Minh Thong, Le Trung Hieu, Adam Mechler

**Affiliations:** Institute of Research and Development, Duy Tan University Danang 550000 Vietnam vovanquan2@duytan.edu.vn; Department of Chemical Engineering, The University of Da Nang – University of Science and Technology Danang 550000 Vietnam; Department of Chemistry, The University of Da Nang – University of Education Danang 550000 Vietnam; The University of Danang, Campus in Kon Tum 704 Phan Dinh Phung Kon Tum Vietnam nmthong@kontum.udn.vn; Hue University of Sciences – Hue University Vietnam; Department of Chemistry and Physics, La Trobe University Victoria 3086 Australia a.mechler@latrobe.edu.au

## Abstract

Oxidative stress is implicated in aging and aging-related diseases, including cancer. Prevention-focused health management approaches emphasize the importance of dietary antioxidants, which naturally draws attention to the antioxidant capacity of natural products. Several groups of plant-derived antioxidant compounds have been identified and their radical scavenging activity confirmed and measured; it has proven challenging, however, to link the experimentally determined activity quantitatively to a molecular mechanism of action. Based on our success with a computational approach, in this study, the methylperoxyl radical scavenging activity of 12 natural stilbenes was evaluated based on kinetic and thermodynamic calculations. The results suggest that for stilbenes hydrogen atom transfer (HAT) is a main mechanism for the ROO˙ radical scavenging in the gas. Assessing the role of substitutes on the antioxidant properties of stilbenes revealed that the presence of O–H groups in ring B can increase the antioxidant activity due to a decrease in the bond dissociation energy (BDE) of the O4′–H, while the replacement of a H atom in the O–H groups by a methyl group reduces the radical scavenging capacity. Among the studied compounds, astringin is a promising antioxidant with the low BDE(O–H) value (73.4 kcal mol^−1^) and the high rate constants (3.36 × 10^6^, 4.11 × 10^3^ and 9.31 × 10^8^ M^−1^ s^−1^ in the gas phase, pentyl ethanoate and water, respectively) that suggest higher activity than *trans*-resveratrol.

## Abstract 2

Oxidative stress is implicated in aging and aging-related diseases, including cancer.

## Graphical abstract

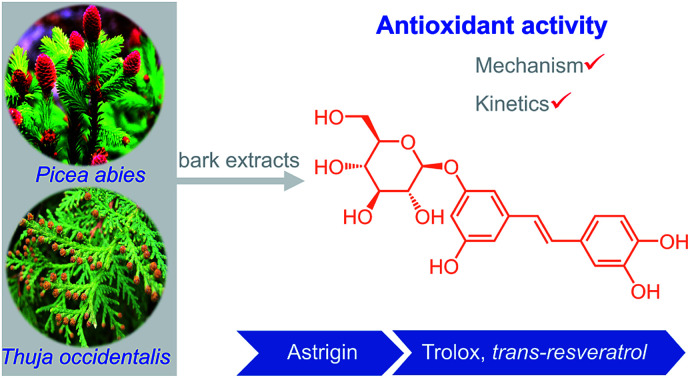

## 1. Introduction

Stilbenes exhibit diverse biological activities including anticancer^1,2^ and anti-inflammatory^3,4^ activity. Natural stilbenes are also good antioxidants^5^ and thus the radical scavenging activity of this class of compounds is an active area of research.^6–16^ As one of the antioxidant polyphenols found in red wine, resveratrol and its derivatives gained much attention.^6,8,9,11,12,15,17,18^ Several studies demonstrated that the radical scavenging abilities of resveratrol and its metabolite piceatannol are higher than that of Trolox, the universally accepted benchmark.^6,8,11,15^ The effect of derivatization of resveratrol has also been studied: longer conjugated chain or complexation with macromolecules could improve the antioxidant activity.^12,17^ Experimental studies also confirmed the potential antioxidant properties of resveratrol and its derivatives.^8,9,18^ A particularly interesting result was the realization that the presence of O3′–H group could increase the antioxidant activity of piceatannol due to the intramolecular hydrogen bond between O3′ and O4′ in the ring B of the molecule.^9^ These reports infer that there can be natural or artificial stilbenes of even higher activity.

However, studies only focused on representative stilbenes *e.g.* resveratrol and piceatannol thus far, while other stilbenes, that are nevertheless also promising antioxidants, have been overlooked. No attention was paid to the effect of substitutes, such as hydroxyl, methoxyl and glucose groups in the aromatic rings, even though it is known that the presence or absence of these moieties can alter the antioxidant properties of phenolic compounds.^8,9,19^ Studies of the thermodynamics and kinetics of antioxidants mainly focused on the HOO˙ and HO˙ scavenging activity,^11,15^ however, the high reactivity of HO˙ and H-bond interactions of H–OO˙ with antioxidants may affect the results.^20^ Larger radicals (ROO˙) are also abundant in biological systems,^15^ yet not much attention was paid to the activity of antioxidants against these radicals.

The purpose of this study is to investigate the influence of various substitutes in the aromatic rings on the BDE(O–H) values and the radical scavenging capacity of natural stilbenes. Potential energy surfaces (PES) and kinetics will be calculated for the initial reactions in the auto-oxidation mechanism of representative antioxidants with methylperoxyl radical (CH_3_OO˙).

## 2. Computational methods

The thermochemical properties: bond dissociation energies (BDEs), ionization energies (IEs), and proton affinities (PAs) of the compounds were obtained following (RO)B3LYP/6-311++G(2df,2p)//B3LYP/6-311G(d,p) methods that are well established in the literature and best suited for thermodynamic calculations yielding data that are in good agreement with experimental values.^19,21–27^ Kinetic calculations were performed in the gas phase as well as in water and pentyl ethanoate solvents (the solvation model density (SMD)) by using the M05-2X/6-311++G(d,p) method and the Eyringpy code,^28–31^ following the quantum mechanics based test for overall free radical scavenging activity (QM-ORSA) protocol.^30,32–35^ The conventional transition state theory and 1 M standard state at 298.15 K were applied to compute rate constant (*k*) according to the equation:^35–40^
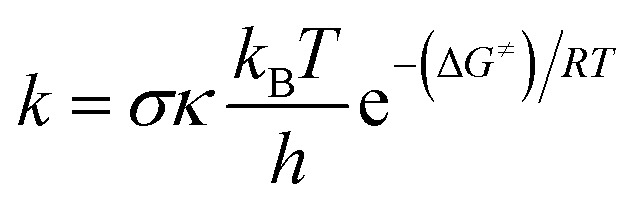
where *σ* is the reaction symmetry number that represents reaction path degeneracy (which was calculated following the literature^41,42^), *κ* accounts for tunneling corrections which were calculated using Eckart barrier,^43^*k*_*B*_ and *h* are the Boltzmann and Planck constants, respectively, ΔG^≠^ is Gibbs free energy of activation of the studied reaction. The SET reaction barriers were corrected by using the Marcus theory,^44,45^ whereas, the Collins–Kimball theory was used to calculate the apparent rate constants (*k*_app_) in solvents.^46^ The diffusion limit rate constant (*k*_D_) for an irreversible bimolecular reaction was calculated according to the literature.^30,47^ The overall rate constant (*k*_overall_), and branching ratios (*Γ*) were computed following the QM-ORSA model.^30^

For the species that have multiple conformers, all of these were investigated and the conformer with the lowest electronic energy was included in the analysis. The correction of transition states was confirmed by the calculating of intrinsic coordinate calculations (IRCs) and the presence of only one single imaginary frequency. The enthalpy value for the hydrogen atom in the gas phase and solvents was calculated at the same level of theory, while the calculated enthalpies of the proton (H^+^) and electron (e^−^) were taken from the literature.^48–51^ All of the calculations were performed with the Gaussian 09 suite of programs.^52^ Atom-in-molecule (AIM) analysis^53^ was performed at the B3LYP/6–311 G(d,p) level by using AIM2000 software.^54^

## 3. Results and discussions

### 3.1. Evaluation of the radical scavenging mechanism

Antioxidant activity follows either of three mechanisms: (1) hydrogen atom transfer (HAT); (2) sequential electron transfer proton transfer (SETPT); and (3) sequential proton loss electron transfer (SPLET).^20,23,55–58^ Thermochemical characteristics of the first step in each mechanism are used to compare the activities of different moieties within a single compound, or to compare different compounds. The HAT mechanism is characterized by bond dissociation energy values, while ionization energies (IEs) and proton dissociation affinities (PDAs) define the SETPT pathway. Proton affinities (PAs) and electron transfer energy (ETE) values are the descriptors of the SPLET mechanism. The comparison of the thermodynamics of the first step is then used to identify which mechanism is preferred for a given compound. Therefore, to identify the main antioxidant mechanism of each studied compound, the thermochemical parameters (BDEs, PAs and IEs) were calculated first by using the ROB3LYP/6-311++G(2df,2p)//B3LYP/6-311G(d,p) method,^23^ in the gas phase and shown in the Table 1.

**Table 1:** The calculated PAs and IEs of the studied compounds (in kcal mol^−1^)

Comp.	O–H position	BDE	PA	IE
1	O3–H	89.5	342.7	168.7
	O5–H	87.8	341.2	
2	O3–H	89.2	343.2	168.7
3	O3–H	88.3	345.2	182.9
	O5–H	86.9	344.1	
4	O3–H	89.3	343.9	161.9
	O5–H	87.6	342.4	
	O4′–H	82.3	334.9	
5	O3–H	88.5	335.0	161.9
	O3′–H	76.9	327.1	
	O4′–H	73.4	322.2	
6	O3–H	87.9	337.7	159
	O4′–H	82.7	336.0	
7	O3–H	88.4	335.5	158.4
	O3′–H	85.9	341.8	
8	O4′–H	81.3	336.4	158.2
9	O3–H	88.6	344.1	156.7
	O5–H	86.8	342.6	
10	O3–H	88.6	343.2	157.2
	O5–H	87.1	341.9	
	O4′–H	81.5	338.9	
11	O3–H	88.7	343.6	160.7
	O5–H	87.1	342.3	
	O3′–H	77.4	330.3	
	O4′–H	73.1	326.0	
12	O3–H	88.5	345.0	157.5
	O5–H	86.9	343.5	
	O2′–H	79.9	333.2	
	O4′–H	80.1	332.4	

The results in Table 1 show that the IE values were in the range of 156.7–182.9 kcal mol^−1^. The lowest IE value was determined for compound 9, whereas that of compound 3 was the highest at 182.9 kcal mol^−1^. On the basis of the calculated IE values, the sequential electron transfer of the studied compounds follows the sequence: 9 < 10 < 12 < 8 < 7 < 6 < 11 < 5 ≈ 4 < 1 ≈ 2 < 3

Considering whether deprotonation is a viable first step in the radical scavenging mechanism, as in SPLET, the PA values of O–H bonds were also calculated. The values are in the range of 322.2–345.2 kcal mol^−1^ in the gas phase. The deprotonation occurs much easier in the O4′–H bond than in the other bonds. Additionally, the presence of O3′–H groups can reduce the PA of O4′–H bond. Thus the lowest PA values were observed at compounds 5 and 11 with PA = 322.2 and 326.0 kcal mol^−1^, respectively. Based on these data SPLET could also occur in the reactions of 5 and 11 with free radicals.

BDE(O–H) values were calculated for assessing the possibility of HAT mechanism; in the gas phase the values were in the range of 73.1–89.9 kcal mol^−1^ (Table 1). It is generally acknowledged that the antioxidant mechanism of phenolic compounds follows the HAT mechanism if the absolute values of ΔBDE and ΔIE compared to phenol (BDE(O–H) = 87.7 kcal mol^−1^, IE_(adiabatic)_ = 193.8 kcal mol^−1^ at the same model chemistry) are higher than 10 kcal mol^−1^ and 36 kcal mol^−1^, respectively.^23,59^ The ΔBDEs and ΔIEs of the studied compounds are in the range of −14.6 to 2.2 and to −37.1 to −10.8 kcal mol^−1^, respectively, making HAT the most likely mechanism for the radical scavenging activity of stilbenes.

To confirm HAT as the most likely mechanism, the free energy change of the first step was calculated for each mechanism in a reaction with CH_3_OO˙ radicals, in vacuum. The results are shown in Table S1, ESI.† It was found that the reactions following the sequential proton and sequential electron transfer mechanisms are not spontaneous in the studied environment; only the HAT pathway yielded exothermic and spontaneous reactions. Hence the SETPT and SPLET mechanisms are generally not favored for the ROO˙ radical scavenging activity of any of the studied stilbenes. On the basis of the calculated data, the HAT mechanism appears to be the main radical scavenging pathway for the neutral stilbenes in the gas phase.

### 3.2. Effects of substitutes on BDEs and antioxidant activity

**Fig. 1:**
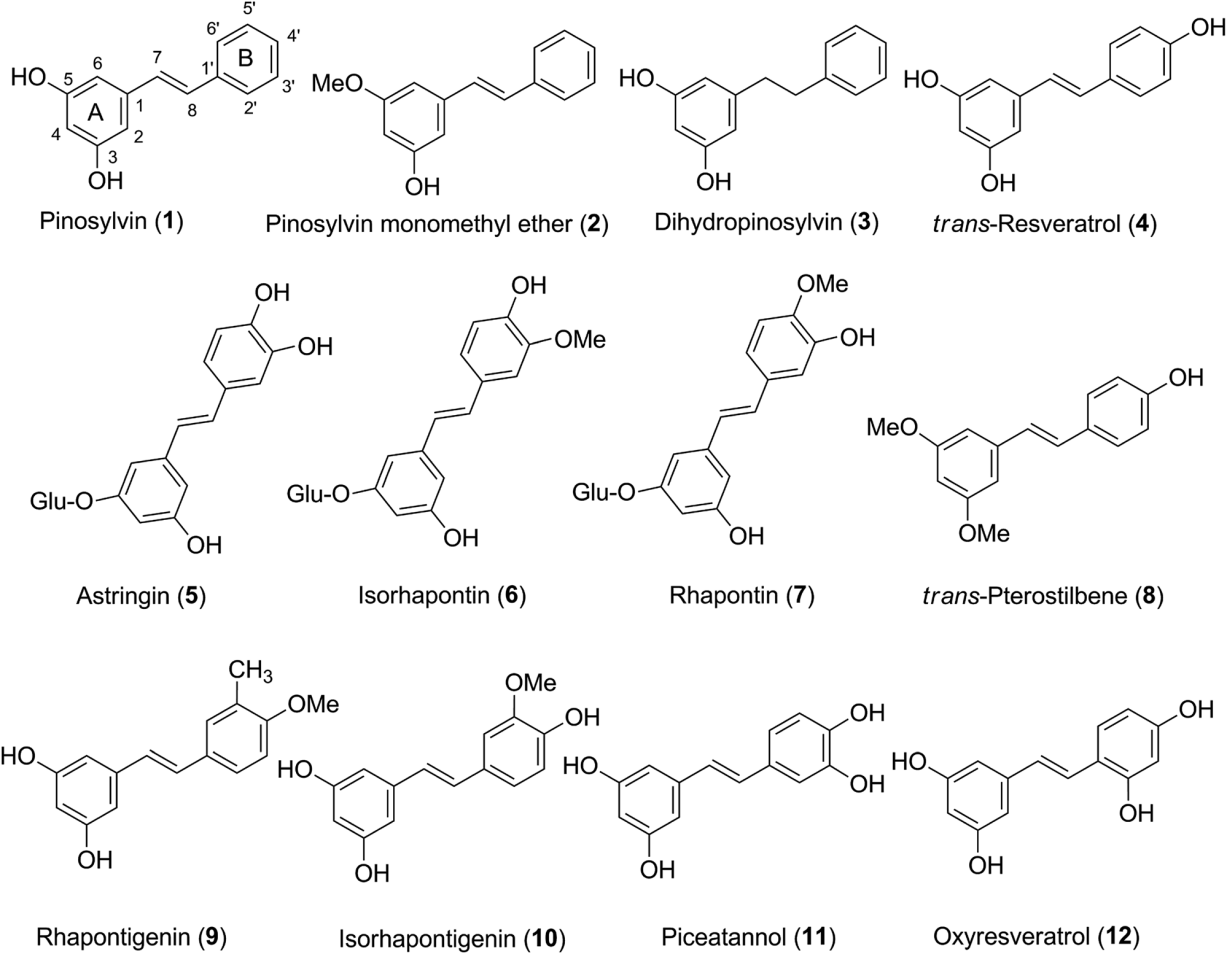
Structures of the 12 stilbenes studied here for their antioxidant properties.

Assessing the co-relationship between substitutes at rings A and B revealed that the BDE values of O–H bonds in the ring A are not affected by substitutes in the ring B, whereas the presence of O–H groups in position 2′ or 3′ at the ring B have significant impacts on the BDE value of O4′–H. In particular the presence of O3′–H group can reduce the BDE value of O4′–H by ∼9 kcal mol^−1^ (4 and 11 in Fig. 1), while the presence of O2′–H group can reduce this figure by ∼2 kcal mol^−1^ (compounds 4 and 12 in Fig. 1). Furthermore the substitution of O–H groups with methoxy groups can increase the BDE value of other O–H bonds at ring B, whereas the BDE values of O3–H bonds at ring A were not affected by the presence of the O5–glucose group (compounds 5, 6 and 7). For example, the BDE(O4′–H) increases by 8.4 kcal mol^−1^ (Table 1) when replacing O3′–H group by methoxy group (observed at O4′–H of compound 11 and compound 10). Thus the O–H groups at the ring B must play a key role in the antioxidant activity of stilbenes due to their low BDE values.

**Fig. 2:**
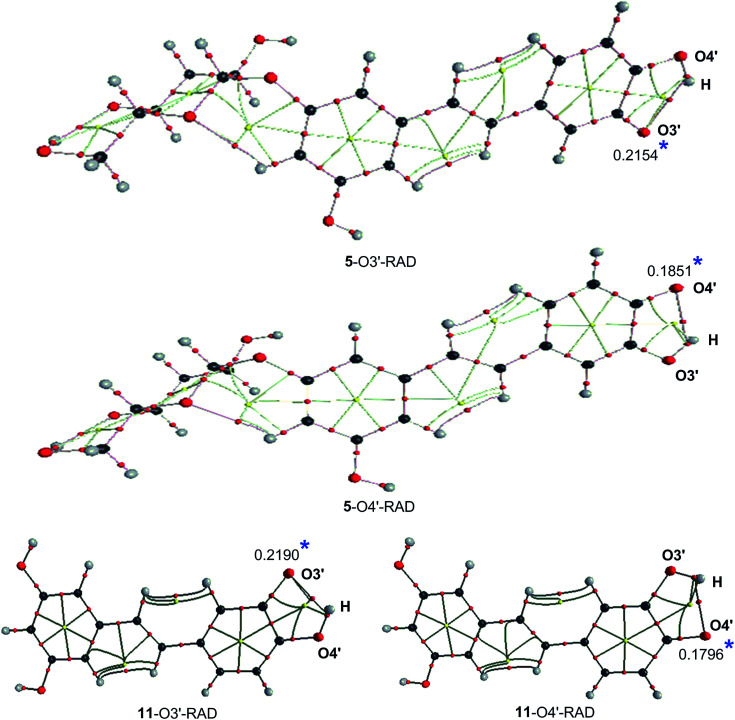
Topological shapes of radicals formed from compounds 5 and 11: (*) Mulliken atomic spin density.

To gain further insights into the effect of the O3′–H group on the BDEs of O4′–H in the ring B, atom in molecule analysis was used to investigate the intermolecular interactions of the 5–O3′–RAD, 5–O4′–RAD, 11–O3′–RAD and 11–O4′–RAD radicals. Topological shapes and selected parameters at the BCPs at intermolecular contacts of the radicals were presented at Fig. 2 and Table 2. The formation of intermolecular interactions was observed between H atom of the neighbor O–H group with the O atom of the radicals, with E_HB_ values in the range of −7.4 to −6.9 kcal mol^−1^. The positive values of ∇^2^*ρ*(r) (0.1018–0.1048 a.u.), *H*(r) (0.0014–0.0017 a.u.) and *G*(r)/|*V*(r)| (1.0582–1.0794) > 1 suggest that this intermolecular contact is a weak hydrogen bond.^60^ The formation of a five-membered ring critical point (RCP), marked by a small yellow ball in the ring of C3′–C4′–O4′–H–O3′ atoms (Fig. 2) can also contribute to the stability of the formed radicals. The analysis suggests that a single electron of the O atom of the radical is released to the five-membered ring and the whole molecule, resulting in a reduction of the atomic spin density of the O atom of the radical (0.1796–0.2190, Fig. 2), hence stabilizing the radical.

**Table 2:** Selected parameters at the bond critical points (BCPs) at intermolecular contacts for radicals formed by 5 and 11 (B3LYP/6-311G(d,p))

Contacts	*ρ*(r) (a.u.)	∇^2^*ρ*(r) (a.u.)	*G*(r)^a^^(a.u.)^	*V*(r)^b^^(a.u.)^	*G*(r)/|*V*(r)|	*H*(r)^c^^(a.u.)^	*E*_HB_ d (kcal mol^−1^)
5**–O3′–RAD**							
O4′–H⋯O3′	0.0286	0.1034	0.0244	−0.0229	1.0640	0.0015	−7.2
RCP	0.0270	0.1482	0.0322	−0.0274	1.1771	0.0048	−8.6

5**–O4′–RAD**							
O3′–H⋯O4′	0.0274	0.1018	0.0237	−0.0220	1.0794	0.0017	−6.9
RCP	0.0262	0.1415	0.0307	−0.0261	1.1783	0.0046	−8.2

11**–O3′–RAD**							
O4′–H⋯O3′	0.0292	0.1048	0.0248	−0.0235	1.0582	0.0014	−7.4
RCP	0.0274	0.1516	0.0330	−0.0280	1.1769	0.0050	−8.8

11**–O4′–RAD**							
O3′–H⋯O4′	0.0279	0.1031	0.0241	−0.0224	1.0746	0.0017	−7.0
RCP	0.0265	0.1446	0.0314	−0.0267	1.1783	0.0048	−8.4

a electron kinetic energy density.

b electron potential energy density.

c total electron energy density.

d individual energies of each hydrogen bond.

It was suggested that H-abstraction could occur at the O3′–H bond of the catechol moiety of 11 to from a stable quinone.^61^ Therefore the donation of the hydrogen atom at the O3′–H bond of compound 5 and 11 was also investigated. The results show that the BDE values of the O3′–H bond at 5–O4′ and 11–O4′ radicals are 76.4 and 76.0 kcal mol^−1^, respectively. Thus the second H-abstraction at the O3′–H bond may contribute to the antioxidant activity of 5 and 11, however the lower BDE values of the O4′–H bond (73.1 and 73.4 kcal mol^−1^) suggest that dissociation of the O4′–H bond defines the antioxidant properties of these compound in the HAT mechanism.

On the basis of the gas phase BDE values, the H-donation ability of the studied compounds follows the sequence 11 ≈ 5 > 12 > 8 ≈ 10 > 4 ≈ 6 > 7 > 9 > 3 > 1 > 2. Therefore compound 5 and 11 are the most potent antioxidants with the lowest BDE(O–H) in the range of 73.1–73.4 kcal mol^−1^ in the gas phase that is much lower than those of *trans*-resveratrol 4 (BDE(O–H) 82.3 kcal mol^−1^). The results suggest that astringin 5 and piceatannol 11 have substantially higher radical scavenging potential than *trans*-resveratrol 4.

### 3.3. The reaction of CH_3_OO˙ radical with stilbenes following the HAT mechanism in the gas phase

#### 3.3.1 Potential energy surfaces (PES)

**Fig. 3:**
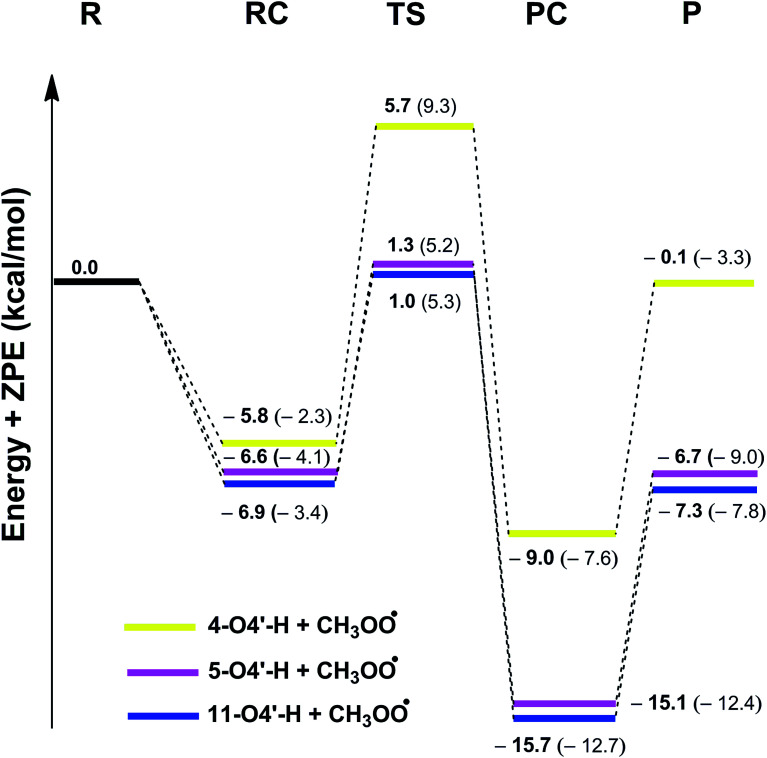
Potential Energy Surfaces of the reactions between selected stilbenes and CH_3_OO˙ following HAT mechanism in the gas phase. The energies of the same states in aqueous phase are also shown for comparison (in brackets).

**Fig. 4:**
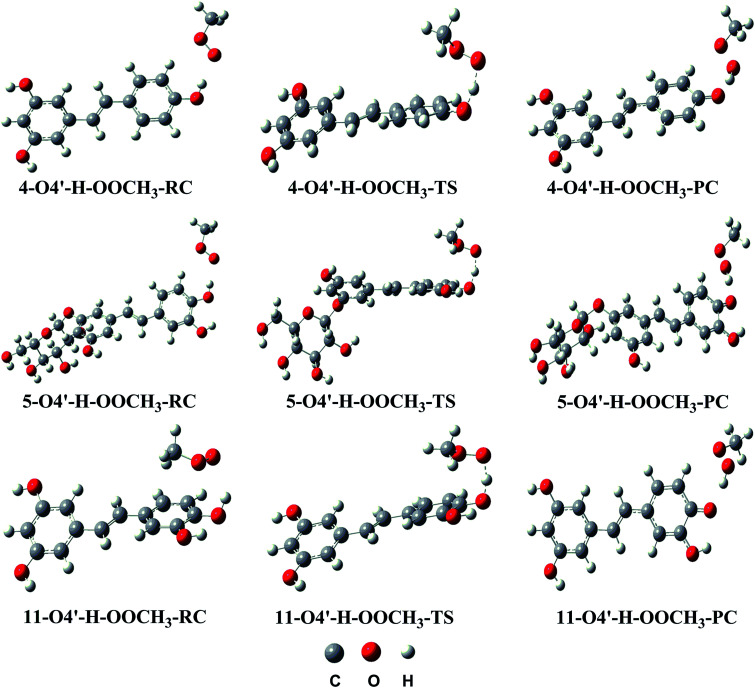
Optimized geometries of RC, TS and PC for the H abstraction channel of reaction between 4, 5 and 11 and the CH_3_OO˙ radical in the gas phase.

To better understand the mechanisms of stilbenes in the gas phase, potential energy surfaces were calculated for the reaction between CH_3_OO˙ and the most active antioxidants 4, 5, and 11. The calculated Gibb free energies of the radical adduct formation (RAF) of CH_3_OO˙ radicals into the C=C double bonds (C7 and C8) (Table S2, ESI†) indicate that the reactions following the RAF mechanism are not spontaneous (Δ*G* > 0) in the studied environment. Thus addition reactions of CH_3_OO˙ radicals into the C=C double bonds did not have any contributions to the radical scavenging activity of stilbenes. This result is in good agreement with previous studies.^11,15^ Therefore, the PES was only studied for the HAT mechanism of the lowest O4′–H bonds with CH_3_OO˙. The results are shown in Fig. 3 and the Optimized geometries of RC, TS and PC are presented in Fig. 4. (reagent (R), pre-complex (RC), transition state (TS), post-complex (PC) and product (P)).

As depicted in Fig. 3, in the gas phase the barrier heights of the reaction of the O4′–H bond of 4, 5 and 11 with CH_3_OO˙ following the HAT mechanism are in the range of 7.9 to 11.5 kcal mol^−1^. Compounds 5 and 11 have the highest antioxidant activity due to the lowest barrier heights at 7.9 kcal mol^−1^. That is 1.45 times lower than that for compound 4. This result is consistent with the calculated BDE values (BDE(5–O4′–H) = 73.4 kcal mol^−1^ and BDE(11-O4′–H) = 73.1 kcal mol^−1^).

Calculating the PESs of the reactions in aqueous solution yielded barrier heights in the range of 8.7 to 11.6 kcal mol^−1^, *i.e.* higher than those in the gas phase, as expected; however the CH_3_OO˙ radical scavenging abilities of 5 and 11 are still higher than that of compound 4 (the barrier heights of the reactions are 8.7 and 9.3 kcal mol^−1^*vs.* 11.6 kcal mol^−1^, respectively).

#### 3.3.2 Kinetic study

Kinetic analysis is a standard method to evaluate antioxidant activity of organic compounds.^11,15,30,62^ In this work transition state theory was used to study the kinetics of model reactions.^36,37^ The Gibbs free energy of activation (Δ*G*^≠^) and rate constants (*k*) were calculated at the M05-2X/6-311++G(d,p) level of theory at 298.15 K in the gas phase and the results are shown in Table 3. It was shown that the reactions between 5 and 11 and CH_3_OO˙ had the highest rate constants with *k*_Eck_ = 3.36 × 10^6^ M^−1^ s^−1^ (Δ*G*^≠^ = 10.9 kcal mol^−1^) and *k*_Eck_ = 3.61 × 10^6^ M^−1^ s^−1^ (Δ*G*^≠^ = 10.8 kcal mol^−1^), respectively, whereas that of 4 was *k*_Eck_ = 8.10 × 10^4^ M^−1^ s^−1^ (Δ*G*^≠^ = 14.5 kcal mol^−1^). The tunneling correction (*κ*) of the reactions of 4 (*κ* = 571.4) with CH_3_OO˙ was more than 10 times higher than these of 5 (*κ* = 48.8) or 11 (*κ* = 47.1). That explains the high rate constant of the CH_3_OO˙ radical scavenging of 4 (*k*_Eck_ = 8.10 × 10^4^ M^−1^ s^−1^), despite of the large activation energy (Δ*G*^≠^ = 14.5 kcal mol^−1^). Thus the HAT H-abstraction rates of 5 and 11 are 41.5 and 44.6 times faster than 4, respectively. These reactions are as fast as the CH_3_OO˙ radical scavenging of Trolox (*k*_Trolox_ = 4.11 × 10^6^ M^−1^ s^−1^). This again suggests that astringin 5 is a highly potent antioxidant and should be considered for preventive medicine along with *trans*-resveratrol 4 and piceatannol 11.

**Table 3:** The calculated Δ*G*^≠^ (kcal mol^−1^), tunneling corrections (*κ*) and *k* (M^−1^ s^−1^) at 298.15 K in the gas phase

Reactions	Δ*G*^≠^	*κ*	*k*_Eck_
4–O41–H + CH_3_OO˙	14.5	571.4	8.10 × 10^4^
5–O41–H + CH_3_OO˙	10.9	48.8	3.36 × 10^6^
11–O41–H + CH_3_OO˙	10.8	47.1	3.61 × 10^6^
Trolox + CH_3_OO˙	10.9	63.4	4.11 × 10^6^

### 3.4. The CH_3_OO˙ scavenging of the most active antioxidants in physiological environments

Kinetics of the CH_3_OO˙ radical scavenging of the compounds 4, 5 and 11 in water (at pH = 7.40) and pentyl ethanoate as a model for lipid media were computed following the QM-ORSA model.^30^ From previous study, the p*K*_a_ values for compound 4 and 11 were 9.16 and 7.86.^11^ The p*K*_a_ was calculated by using the model reaction (1) below, following the literature:^63^

1 5–O4′–H + Ref^−^ → 5–O4′^−^ + HRef

The value of p*K*_a_ was defined by eqn (2):^63–65^

2 p*K*_a_ = Δ*G*_s_/*RT* ln(10) + p*K*_a_(HRef)

where the HRef is catechol with the experimental p*K*_a_(O4′–H) = 10.09.^66^ The calculated p*K*_a_(O4′–H) value in this work is 7.83. The result are in a good agreement with the previous data of the compound 11 (p*K*_a_ = 7.86)^11^. Thus under physiological conditions (pH 7.40), compounds 4, 5 and 11 exist both in the neutral state (98.3%, 73.4% and 74.2%) and anion state (1.70%, 26.6% and 25.8%, at O4′), respectively. These forms were used to evaluate the radical scavenging of the studied antioxidants. A previous study showed that the HAT mechanism at the O4′–H bond decided the overall rate constant (*k*_overall_) of the HOO˙ radical scavenging of compound 4 in lipid media, whereas that for 11 in was defined by the HAT mechanism at O3–H and O4′–H bonds.^11^ However, in polar solvent the radical scavenging was defined by the SET mechanism (for the anion state) for both compounds 4 and 11.^11^ Thus in this study the *k*_overall_ of the CH_3_OO˙ radical scavenging of 4, 5 and 11 was calculated according to the equationsin lipid media:

3 *k*_overall_ (4) = *k*_(HAT)_ (O4′–H)

4 *k*_overall_ (5 or 11) = *k*_(HAT)_ (O3′–H) + *k*_(HAT)_ (O4′–H)

in the aqueous solution:

5 *k*_overall_ (4) = 0.017 × *k*_(SET)_

6 *k*_overall_ (5) = 0.266 × *k*_(SET)_

7 *k*_overall_ (11) = 0.258 × *k*_(SET)_

As shown in Table 4, the overall rate constants of the lead compounds + CH_3_OO˙ reaction in the aqueous solution (*k*_overall_ = 1.29 × 10^7^ to 9.31 × 10^8^ M^−1^ s^−1^) is about 10^5^ times higher than these for the lipid media (*k*_overall_ = 5.80 × 10^2^ to 5.90 × 10^3^ M^−1^ s^−1^). In the aqueous solution, the SET mechanism defined the CH_3_OO˙ radical scavenging of the studied stilbenes. The 5 + CH_3_OO˙ reaction in the aqueous solution is 2.25 and 72.2 times faster than that of compounds 11 and 4, respectively. However, the HAT mechanism played a key role in the lipid media. The highest *k*_overall_ was observed at the CH_3_OO˙ radical scavenging of 11 with *k*_overall_ = 5.90 × 10^3^ M^−1^ s^−1^. That is 1.4 and 10.2 times higher than these of 5 and 4 in non-polar solvent, respectively. Thus, the results again confirm that the radical scavenging of astringin is as high as piceatannol in biological environments. According to these results the ROO˙ scavenging of 5 is higher than that of Trolox and *trans*-resveratrol in both aqueous and lipid media.^11,30^ Thus 5 is a promising natural antioxidant.

**Table 4:** The calculated Δ*G*^≠^ (in kcal mol^−1^), *k*_app_ (M^−1^ s^−1^) and *Γ* (%) of the studied compounds + CH_3_OO˙ reaction in water and pentyl ethanoate solvents

Comp.	Mechanism		Water			Pentyl ethanoate		
			Δ*G*^≠^	*k*_app_	*Γ*	Δ*G*^≠^	*k*_app_	*Γ*
4	SET		5.3	7.60 × 10^8^	100			
	HAT	O4′				18.1	5.80 × 10^2^	100
	*k*_overall_			1.29 × 10^7^			5.80 × 10^2^	
5	SET		4.1	3.50 × 10^9^	100			
	HAT	O3′				19.4	1.20 × 10^1^	0.3
		O4′				15.3	4.10 × 10^3^	99.7
	*k*_overall_			9.31 × 10^8^			4.11 × 10^3^	
11	SET		4.8	1.60 × 10^9^	100			
	HAT	O3′				20.1	4.2	0.1
		O4′				14.9	5.90 × 10^3^	99.9
	*k*_overall_			4.13 × 10^8^			5.90 × 10^3^	

## 4. Conclusions

The methylperoxyl radical scavenging activity of 12 natural stilbenes was investigated by kinetic and thermodynamic calculations. It was found that HAT is the main radical scavenging mechanism for these stilbenes in the gas phase and in solutions. The presence of O–H groups in ring B can increase the antioxidant activity due to a decrease in the BDE(O4′–H) values. Replacement of the H atom at O–H groups by the methyl group can reduce antioxidant activity, while replacement of the glucose group has no effect. Among of the studied compounds, astringin is a promising antioxidant with the low BDE(O4′–H) at 73.4 kcal mol^−1^ and the HAT H-abstraction rate at 3.36 × 10^6^ M^−1^ s^−1^ at 298.15 K in the gas phase and 4.11 × 10^3^ M^−1^ s^−1^, 9.31 × 10^8^ M^−1^ s^−1^ in the lipid and aqueous media, respectively. Astringin shows similar antioxidant activity to piceatannol but higher than *trans*-resveratrol. These results suggest that natural astringin should be considered for preventive/therapeutic use along with piceatannol and *trans*-resveratrol.

## Conflicts of interest

There are no conflicts to declare.

## Supplementary Material

RA-009-C9RA08381B-s001
