# Supplementary material for: A theoretical study of the radical scavenging activity of natural stilbenes
Source: RSC Adv. 2019 Dec 18;9(72):42020–8. doi: 10.1039/c9ra08381b (PMC9076562; doi:10.1039/c9ra08381b)
Supplement: RA-009-C9RA08381B-s001 [file RA-009-C9RA08381B-s001.pdf]

Electronic Supplementary Material (ESI) for RSC Advances.
This journal is © The Royal Society of Chemistry 2019

Supporting information (SI)

A theoretical study of the radical scavenging activity of natural stilbenes

Quan V. Vo,[1*] Pham Cam Nam,[2] Mai Van Bay,[3] Nguyen Minh Thong,[4] Le Trung Hieu,[5] Adam Mechler[6*]

[1]Institute of Research and Development, Duy Tan University, Danang 550000, Vietnam

[2]Department of Chemical Engineering, The University of Da Nang - University of Science and Technology, Danang 550000, Vietnam

[3]Department of Chemistry, The University of Da Nang - University of Education, Danang 550000, Vietnam

[4]The University of Danang, Campus in Kon Tum, 704 Phan Dinh Phung, Kon Tum, Vietnam

[5]Hue University of Sciences – Hue University

[6]Department of Chemistry and Physics, La Trobe University, Victoria 3086, Australia

*Corresponding author: vovanquan2@duytan.edu.vn, a.mechler@latrobe.edu.au

Table of contents

Table S1: The calculated thermal properties (in Kcal/mol) of the reaction between the studied compounds (for the lowest PAs(X–H)) with $CH_3OO^•$ radical *via* the sequential proton, SET processes and HAT mechanism using the ROB3LYP/6-311G++G(2df,2p)//B3LYP/6-311G(d,p) methods in the gas phase. S2

Table S2: The calculated Gibb energies (in kcal/mol) of the reaction between the studied compounds with $CH_3OO^•$ radical *via* RAF mechanism using the ROB3LYP/6-311G++G(2df,2p)//B3LYP/6-311G(d,p) methods in the gas phase. S3

Table S3: Cartesian coordinates and molecular enthalpies of all parent molecules, radicals and anions optimized at B3LYP/6-311G(d,p) level in the gas phase. S4

Table S4: Cartesian coordinates and molecular enthalpies of the TS optimized at M05-2X/6-311++G(d,p) level in the studied environments. S82

Table S1: The calculated thermal properties (in Kcal/mol) of the reaction between the studied compounds (for the lowest PAs(X−H)) with $CH_3OO^{\bullet}$ radical *via* the sequential proton, SET processes and HAT mechanism using the ROB3LYP/6-311G++G(2df,2p)//B3LYP/6-311G(d,p) methods in the gas phase.

comp.	PA mechanism		SET mechanism		HAT mechanism	
	ΔH	ΔG	ΔH	ΔG	ΔH	ΔG
**1**	167.4	164.6	141.0	139.9	4.0	1.1
**2**	169.4	166.3	139.1	137.8	5.4	2.6
**3**	170.3	169.4	155.2	155.4	3.1	2.6
**4**	161.1	158.7	134.3	133.2	-1.5	-3.7
**5**	148.4	149.2	134.2	135.4	-10.4	-9.9
**6**	162.2	159.7	131.3	129.9	-1.1	-3.4
**7**	161.7	161.1	130.7	131.6	2.1	1.7
**8**	162.6	162.9	130.5	132.1	-2.5	-1.8
**9**	168.8	168.5	129.0	130.8	3.0	3.3
**10**	165.1	164.8	129.6	130.7	-2.3	-2.2
**11**	152.2	152.7	133.0	134.4	-10.7	-10.1
**12**	158.6	159.1	129.8	131.9	-3.9	-4.3

Table S2: The calculated Gibb energies (in kcal/mol) of the reaction between the studied compounds with $CH_3OO^{\bullet}$ radical *via* RAF mechanism using the ROB3LYP/6-311G++G(2df,2p)//B3LYP/6-311G(d,p) methods in the gas phase.

comp.	C=C position	ΔG
**1**	C7	10.1
	C8	9.6
**2**	C7	9.8
	C8	10.0
**3**		
**4**	C7	9.6
	C8	9.6
**5**	C7	11.6
	C8	10.6
**6**	C7	13.9
	C8	11.5
**7**	C7	11.4
	C8	10.4
**8**	C7	11.8
	C8	12.8
**9**	C7	11.7
	C8	12.7
**10**	C7	10.6
	C8	10.9
**11**	C7	11.4
	C8	12.2
**12**	C7	9.5
	C8	8.8

Table S3: Cartesian coordinates and molecular enthalpies of all parent molecules, radicals and anions optimized at B3LYP/6-311G(d,p) level in the gas phase.

**Name of compound (1)**	Pinosylvin
Cartesian Coordinates optimized at B3LYP/6-311G(d,p)	Energies at B3LYP/6-311G(d,p) in gas phase
C 4.04960200 0.17936800 -0.00013600	Zero-point correction= 0.221958 (Hartree/Particle)
C 3.52080100 -1.11013100 -0.00090500	Thermal correction to Energy= 0.234890
C 2.14208100 -1.30923600 -0.00070300	Thermal correction to Enthalpy= 0.235835
C 1.26320300 -0.21224300 0.00022200	Thermal correction to Gibbs Free Energy= 0.182011
C 1.79666000 1.08288400 0.00104900	Sum of electronic and zero-point Energies= -691.106438
C 3.17658600 1.26807700 0.00084800	Sum of electronic and thermal Energies= -691.093505
H 5.12610700 0.31058300 -0.00027600	Sum of electronic and thermal Enthalpies= -691.092561
H 1.74219200 -2.31893900 -0.00129700	Sum of electronic and thermal Free Energies= -691.146385
H 1.16549700 1.96110600 0.00197700	
C -1.17038500 0.42251500 -0.00019500	
H -0.91075200 1.47770500 -0.00097400	
C -2.61155500 0.15827800 -0.00004600	
C -3.49713200 1.24922300 -0.00238400	
C -3.16677800 -1.13439100 0.00238800	
C -4.87571300 1.06282500 -0.00242900	
H -3.09189900 2.25591000 -0.00422600	
C -4.54230800 -1.32107400 0.00234400	
H -2.51819300 -2.00243000 0.00446500	
C -5.40616200 -0.22436800 -0.00007900	
H -5.53527800 1.92350700 -0.00428500	
H -4.94624100 -2.32751800 0.00427400	
H -6.47969400 -0.37461700 -0.00007800	
H -0.43318500 -1.54027600 0.00068400	
C -0.17765500 -0.48408900 0.00031700	
O 4.41303200 -2.14462600 -0.00182500	
H 3.92952300 -2.97702800 -0.00252300	
O 3.63084800 2.55622600 0.00169900	
H 4.59341300 2.55337300 0.00153100	
**Name of radical**	**1-O3**
Cartesian Coordinates optimized at B3LYP/6-311G(d,p)	Energies at B3LYP/6-311G(d,p) in gas phase
C -4.11388400 0.15174600 -0.00000200	Zero-point correction= 0.208868 (Hartree/Particle)
C -3.60593800 -1.20496500 0.00002700	Thermal correction to Energy= 0.222423
C -2.16527000 -1.37735000 0.00003500	Thermal correction to Enthalpy= 0.223367
C -1.29398200 -0.29922800 0.00001500	Thermal correction to Gibbs Free Energy= 0.165056
C -1.84674500 1.00483500 -0.00001000	Sum of electronic and zero-point Energies= -690.475106
C -3.24288600 1.21535900 -0.00002000	Sum of electronic and thermal Energies= -690.461551

H -5.19082400 0.28088100 -0.00000600<br>H -1.80181300 -2.39831300 0.00005600<br>H -1.21586400 1.88414500 -0.00001600<br>C 1.12454800 0.38473400 -0.00003800<br>H 0.84595900 1.43530000 -0.00009700<br>C 2.57091400 0.15122400 -0.00002600<br>C 3.43191400 1.26125400 0.00005000<br>C 3.15111200 -1.13010800 -0.00008700<br>C 4.81398600 1.10375000 0.00007800<br>H 3.00592800 2.25933100 0.00009100<br>C 4.53013400 -1.28767600 -0.00006000<br>H 2.52087300 -2.01141700 -0.00016800<br>C 5.37009600 -0.17252500 0.00002500<br>H 5.45580100 1.97756600 0.00014000<br>H 4.95530900 -2.28512300 -0.00011000<br>H 6.44645900 -0.30044500 0.00004500<br>H 0.42233100 -1.59454400 0.00008700<br>C 0.15140300 -0.54298700 0.00002400<br>O -4.37453100 -2.19011500 0.00003700<br>O -3.63754700 2.52027000 -0.00004100<br>H -4.59994800 2.56300400 -0.00006100	Sum of electronic and thermal Enthalpies= -690.460607<br>Sum of electronic and thermal Free Energies= -690.518919
**Name of radical**	**1-O5**
Cartesian Coordinates optimized at B3LYP/6-311G(d,p)	Energies at B3LYP/6-311G(d,p) in gas phase
C 4.12174200 0.23181300 0.02006500<br>C 3.59866900 -1.03788300 0.09773000<br>C 2.20124900 -1.23337800 0.06505500<br>C 1.29063500 -0.15479500 -0.04250000<br>C 1.81223600 1.12735200 -0.13315700<br>C 3.24025000 1.37153600 -0.09990000<br>H 5.19009300 0.40156300 0.04300100<br>H 1.80968500 -2.24527400 0.12981500<br>H 1.18482300 2.00237800 -0.24317900<br>C -1.14634300 0.43219800 0.03094800<br>H -0.89401800 1.48297600 0.14698900<br>C -2.58456700 0.15580600 0.01162700<br>C -3.47594800 1.21415400 0.25439100<br>C -3.12816300 -1.11649600 -0.24147800<br>C -4.85232500 1.01316000 0.25547500<br>H -3.07763000 2.20503300 0.44657900<br>C -4.50163700 -1.31741500 -0.24060400	Zero-point correction= 0.208582 (Hartree/Particle)<br>Thermal correction to Energy= 0.222206<br>Thermal correction to Enthalpy= 0.223151<br>Thermal correction to Gibbs Free Energy= 0.165318<br>Sum of electronic and zero-point Energies= -690.472389<br>Sum of electronic and thermal Energies= -690.458764<br>Sum of electronic and thermal Enthalpies= -690.457820<br>Sum of electronic and thermal Free Energies= -690.515653

H -2.47357500 -1.95440600 -0.45057400<br>C -5.37194600 -0.25474200 0.00907400<br>H -5.51825500 1.84688700 0.44727700<br>H -4.89905300 -2.30636700 -0.44010500<br>H -6.44384600 -0.41580100 0.00679600<br>H -0.38454000 -1.51673000 -0.13581300<br>C -0.14481500 -0.45972000 -0.05934000<br>O 4.45891000 -2.09078600 0.20579400<br>H 3.96203300 -2.91377400 0.25617900<br>O 3.68984600 2.53878100 -0.17720500	
**Name of anion**	1-O3
Cartesian Coordinates optimized at B3LYP/6-311G(d,p	Energies at B3LYP/6-311G(d,p) in gas phase
C 4.09026500 0.20614900 0.00003200<br>C 3.54599300 -1.06374600 -0.00003700<br>C 2.16380700 -1.27440900 -0.00003300<br>C 1.30801300 -0.14536800 0.00003100<br>C 1.84521900 1.13981300 0.00008800<br>C 3.26905200 1.40290000 0.00003500<br>H 5.16846800 0.32851800 0.00013600<br>H 1.75116200 -2.28068200 -0.00018200<br>H 1.20406600 2.01546500 0.00016400<br>C -1.14338600 0.47886700 -0.00001500<br>H -0.89783500 1.53701000 -0.00006600<br>C -2.57226600 0.17954900 -0.00000700<br>C -3.49761300 1.24340700 -0.00009000<br>C -3.10791600 -1.12630800 0.00008100<br>C -4.87046100 1.02205300 -0.00008800<br>H -3.11790100 2.26040000 -0.00015800<br>C -4.47856600 -1.34708300 0.00008200<br>H -2.43781500 -1.97807000 0.00015000<br>C -5.37604100 -0.27678800 -0.00000200<br>H -5.54943300 1.86934400 -0.00015400<br>H -4.85367700 -2.36614200 0.00015200<br>H -6.44620500 -0.45388900 0.00000100<br>H -0.38091100 -1.47217200 0.00006300<br>C -0.12825300 -0.41233900 0.00002500<br>O 4.42128500 -2.14713200 -0.00011700<br>H 3.87977100 -2.94202100 0.00013000<br>O 3.75286800 2.56239100 0.00001100	Zero-point correction= 0.207454 (Hartree/Particle)<br>Thermal correction to Energy= 0.221193<br>Thermal correction to Enthalpy= 0.222137<br>Thermal correction to Gibbs Free Energy= 0.165160<br>Sum of electronic and zero-point Energies= -690.555245<br>Sum of electronic and thermal Energies= -690.541506<br>Sum of electronic and thermal Enthalpies= -690.540562<br>Sum of electronic and thermal Free Energies= -690.597539
**Name of anion**	1-O5

Cartesian Coordinates optimized at B3LYP/6-311G(d,p	Energies at B3LYP/6-311G(d,p) in gas phase
C -4.08143300 0.09579100 0.00009600	Zero-point correction= 0.207822 (Hartree/Particle)
C -3.58426100 -1.26149900 0.00026800	Thermal correction to Energy= 0.221391
C -2.13696800 -1.33795700 0.00005100	Thermal correction to Enthalpy= 0.222335
C -1.30320100 -0.21774000 -0.00021300	Thermal correction to Gibbs Free Energy= 0.165712
C -1.85205200 1.08579500 -0.00028200	Sum of electronic and zero-point Energies= -690.557679
C -3.23965500 1.20044000 -0.00015400	Sum of electronic and thermal Energies= -690.544110
H -5.16331800 0.22649800 0.00005900	Sum of electronic and thermal Enthalpies= -690.543166
H -1.70694200 -2.33631300 0.00011200	Sum of electronic and thermal Free Energies= -690.599788
H -1.24232900 1.97943100 -0.00050000	
C 1.13412000 0.45446000 0.00008400	
H 0.87191200 1.50893500 0.00037900	
C 2.56902100 0.18226300 0.00004300	
C 3.47546200 1.26193200 0.00050700	
C 3.12672700 -1.11415700 -0.00042400	
C 4.85214500 1.06463400 0.00049100	
H 3.07870000 2.27256700 0.00088000	
C 4.50096200 -1.31090000 -0.00043000	
H 2.47119700 -1.97697200 -0.00079500	
C 5.37983100 -0.22520000 0.00002400	
H 5.51653400 1.92348700 0.00085000	
H 4.89333100 -2.32333300 -0.00080400	
H 6.45288900 -0.38390700 0.00000900	
H 0.40363500 -1.51200800 -0.00056800	
C 0.13670600 -0.45686200 -0.00024200	
O -4.32661200 -2.27634300 0.00052300	
O -3.76332500 2.48923600 -0.00034800	
H -4.72053600 2.39246200 0.00006600	

**Name of cationic radical**	Energies at B3LYP/6-311G(d,p) in gas phase
C 4.00115100 0.20262900 0.00001600	Zero-point correction= 0.222313 (Hartree/Particle)
C 3.50114200 -1.12014600 0.00000200	Thermal correction to Energy= 0.236010
C 2.13566400 -1.34724800 -0.00000600	Thermal correction to Enthalpy= 0.236954
C 1.24180400 -0.24851200 -0.00001800	Thermal correction to Gibbs Free Energy= 0.180243
C 1.75276600 1.07723600 -0.00001400	Sum of electronic and zero-point Energies= -690.844122
C 3.12499600 1.29518400 0.00001000	Sum of electronic and thermal Energies= -690.830425
H 5.07705400 0.34008500 0.00002800	Sum of electronic and thermal Enthalpies= -690.829481
H 1.74586300 -2.35860200 0.00002700	Sum of electronic and thermal Free Energies= -690.886192
H 1.10436700 1.94163300 -0.00002300	
C -1.17414100 0.41432700 -0.00001500	
H -0.90165800 1.46444600 -0.00002200	

C	-2.57666900	0.15179200	-0.00000700	
C	-3.46379500	1.26289300	-0.00001800	
C	-3.13240400	-1.15804900	0.00001500	
C	-4.83210800	1.07645000	-0.00000800	
H	-3.05197800	2.26567800	-0.00003400	
C	-4.49973100	-1.33553400	0.00002500	
H	-2.48640700	-2.02645200	0.00002500	
C	-5.35451900	-0.22240700	0.00001300	
H	-5.49903300	1.92946100	-0.00001700	
H	-4.91737200	-2.33462800	0.00004300	
H	-6.42800100	-0.37070200	0.00002200	
H	-0.42827100	-1.57445000	-0.00001000	
C	-0.15940600	-0.52360400	-0.00001500	
O	4.43500100	-2.08640700	0.00001700	
H	4.03343900	-2.96382300	-0.00022300	
O	3.54674200	2.57489000	0.00001700	
H	4.50955100	2.62942600	0.00003700	

**Name of compound (2)**	Pinosylvin monomethyl ether
Cartesian Coordinates optimized at B3LYP/6-311G(d,p)	Energies at B3LYP/6-311G(d,p) in gas phase
C -3.83232100 -0.38112300 -0.01021800	Zero-point correction= 0.250156 (Hartree/Particle)
C -3.20603100 -1.61967000 -0.02032100	Thermal correction to Energy= 0.264474
C -1.81160700 -1.70901900 -0.01358500	Thermal correction to Enthalpy= 0.265418
C -1.03056300 -0.55083700 0.00261400	Thermal correction to Gibbs Free Energy= 0.208311
C -1.66375300 0.70937700 0.01456900	Sum of electronic and zero-point Energies= -730.388250
C -3.05221400 0.78440700 0.00761500	Sum of electronic and thermal Energies= -730.373932
H -4.91250700 -0.28453500 -0.01433100	Sum of electronic and thermal Enthalpies= -730.372987
H -1.35345500 -2.69028900 -0.02233900	Sum of electronic and thermal Free Energies= -730.430095
H -1.06898300 1.61008900 0.03267800	
C 1.35016800 0.27133100 -0.00893900	
H 1.01659200 1.30548500 -0.03330300	
C 2.80708400 0.11346700 -0.00291800	
C 3.61119800 1.26423600 -0.06347900	
C 3.45542300 -1.13348300 0.06202100	
C 4.99970900 1.17943200 -0.06257100	
H 3.13388300 2.23769100 -0.11310100	
C 4.84097600 -1.21887700 0.06278900	
H 2.87244700 -2.04512800 0.11554700	
C 5.62277600 -0.06398900 0.00019100	
H 5.59456600 2.08479400 -0.11078900	

H	5.31713100	-2.19188400	0.11410800	
H	6.70438800	-0.13575800	0.00165600	
H	0.76112600	-1.74011600	0.02131600	
C	0.42723900	-0.70638800	0.00714400	
O	-3.90371500	-2.79388300	-0.03682300	
H	-4.84694300	-2.60135500	-0.03989900	
C	-3.05307700	3.17764800	0.03694800	
H	-3.81191900	3.95844300	0.04194400	
H	-2.42328600	3.29107300	-0.85236500	
H	-2.43334300	3.26985800	0.93572500	
O	-3.76375400	1.94795300	0.01832100	
**Name of radical**	**2-O3**			
Cartesian Coordinates optimized at B3LYP/6-311G(d,p)				Energies at B3LYP/6-311G(d,p) in gas phase
C	-3.89302400	-0.40700700	-0.00000700	Zero-point correction= 0.236849 (Hartree/Particle)
C	-3.28316000	-1.71504500	-0.00011500	Thermal correction to Energy= 0.251860
C	-1.83526600	-1.77777300	-0.00014200	Thermal correction to Enthalpy= 0.252804
C	-1.05929200	-0.63622900	-0.00005800	Thermal correction to Gibbs Free Energy= 0.191593
C	-1.71081000	0.63260500	0.00006100	Sum of electronic and zero-point Energies= -729.754606
C	-3.11125000	0.73713700	0.00007400	Sum of electronic and thermal Energies= -729.739595
H	-4.97299400	-0.33714500	0.00001500	Sum of electronic and thermal Enthalpies= -729.738651
H	-1.39071600	-2.76594200	-0.00022800	Sum of electronic and thermal Free Energies= -729.799861
H	-1.11016800	1.53028700	0.00016600	
C	1.30340200	0.23314400	-0.00004200	
H	0.95199000	1.26168200	-0.00006300	
C	2.76308900	0.10548600	-0.00001100	
C	3.54128300	1.27517800	-0.00035500	
C	3.43585000	-1.12984200	0.00038600	
C	4.93115000	1.21968400	-0.00034800	
H	3.04352900	2.23963000	-0.00065500	
C	4.82278100	-1.18573400	0.00038700	
H	2.87247300	-2.05524900	0.00076300	
C	5.57926900	-0.01241000	0.00001500	
H	5.50715800	2.13823000	-0.00063000	
H	5.31972700	-2.14945000	0.00071100	
H	6.66207600	-0.06146200	0.00002600	
H	0.75106900	-1.79115300	-0.00015500	
C	0.40042200	-0.76341900	-0.00007900	
O	-3.97953500	-2.75685200	-0.00018400	
C	-3.04028800	3.13651000	0.00016600	
H	-3.78193800	3.93309600	0.00017200	

H -2.41525100 3.22743300 -0.89514100<br>H -2.41523200 3.22744200 0.89545800<br>O -3.78129700 1.92296300 0.00018100		
**Name of anion**	2-O3	
Cartesian Coordinates optimized at B3LYP/6-311G(d,p		Energies at B3LYP/6-311G(d,p) in gas phase
C -3.86118100 -0.46418800 0.00004800<br>C -3.25753600 -1.77423000 0.00001700<br>C -1.80698300 -1.73740100 0.00009800<br>C -1.06420000 -0.55846600 0.00016600<br>C -1.71461100 0.70186600 0.00016700<br>C -3.11155600 0.70587100 0.00011100<br>H -4.94396100 -0.39572200 0.00001100<br>H -1.29977600 -2.69915400 0.00007900<br>H -1.14171100 1.61682000 0.00053300<br>C 1.31540500 0.30263100 -0.00015400<br>H 0.97668700 1.33523900 -0.00057100<br>C 2.76646900 0.14088100 -0.00010900<br>C 3.58756600 1.28704700 0.00019300<br>C 3.42275100 -1.10873800 -0.00035700<br>C 4.97522800 1.19693300 0.00028300<br>H 3.11453900 2.26436400 0.00037600<br>C 4.80807500 -1.19846800 -0.00025700<br>H 2.83637400 -2.01986700 -0.00064400<br>C 5.60084800 -0.04838200 0.00006800<br>H 5.57122500 2.10457000 0.00052400<br>H 5.27739900 -2.17759100 -0.00044800<br>H 6.68289800 -0.12411400 0.00013600<br>H 0.73619100 -1.71320600 0.00055600<br>C 0.38893600 -0.68176300 0.00019500<br>O -3.91064800 -2.84852800 -0.00007300<br>C -3.16227500 3.10457000 -0.00044000<br>H -3.92195800 3.88958700 -0.00091800<br>H -2.52928200 3.22141100 -0.89117000<br>H -2.52934700 3.22249300 0.89018900<br>O -3.85821200 1.88180300 0.00022000		Zero-point correction= 0.235743 (Hartree/Particle)<br>Thermal correction to Energy= 0.250745<br>Thermal correction to Enthalpy= 0.251689<br>Thermal correction to Gibbs Free Energy= 0.191333<br>Sum of electronic and zero-point Energies= -729.836856<br>Sum of electronic and thermal Energies= -729.821853<br>Sum of electronic and thermal Enthalpies= -729.820909<br>Sum of electronic and thermal Free Energies= -729.881266
**Name of cationic radical**		Energies at B3LYP/6-311G(d,p) in gas phase
C -3.78895800 -0.39867900 0.00010000<br>C -3.16993100 -1.65209000 -0.00026400<br>C -1.78125000 -1.72024400 -0.00040000<br>C -1.00207800 -0.53426100 -0.00012800		Zero-point correction= 0.250384 (Hartree/Particle)<br>Thermal correction to Energy= 0.265577<br>Thermal correction to Enthalpy= 0.266521<br>Thermal correction to Gibbs Free Energy= 0.206210

C -1.63730400 0.73627300 0.00020500<br>C -3.01949000 0.79615900 0.00031400<br>H -4.86909600 -0.29928500 0.00028000<br>H -1.30543200 -2.69250100 -0.00067800<br>H -1.05146100 1.64200200 0.00036400<br>C 1.36435700 0.29695900 -0.00028500<br>H 1.02308000 1.32721100 -0.00058600<br>C 2.78250400 0.12773100 -0.00007400<br>C 3.59564900 1.29312400 -0.00075000<br>C 3.42225200 -1.14254800 0.00076900<br>C 4.97352600 1.19644900 -0.00067000<br>H 3.11985400 2.26724800 -0.00136000<br>C 4.79865900 -1.23078300 0.00087900<br>H 2.83466600 -2.05139100 0.00139300<br>C 5.57960000 -0.06525200 0.00014400<br>H 5.58333000 2.09121500 -0.00122700<br>H 5.27969500 -2.20098400 0.00156100<br>H 6.66041700 -0.14362500 0.00022400<br>H 0.75541100 -1.73573200 -0.00010900<br>C 0.41566100 -0.70604800 -0.00018100<br>O -3.85074800 -2.81397300 -0.00040900<br>H -4.80351900 -2.66449200 -0.00046000<br>C -3.10785500 3.18962900 0.00011000<br>H -3.90924800 3.92350200 0.00017700<br>H -2.49650200 3.31212800 -0.89840300<br>H -2.49596300 3.31270200 0.89818200<br>O -3.75891300 1.91315900 0.00066200	Sum of electronic and zero-point Energies= -730.128791<br>Sum of electronic and thermal Energies= -730.113599<br>Sum of electronic and thermal Enthalpies= -730.112654<br>Sum of electronic and thermal Free Energies= -730.172965

**Name of compound (3)**	Dihyropinosylvin
Cartesian Coordinates optimized at B3LYP/6-311G(d,p)	Energies at B3LYP/6-311G(d,p) in gas phase
C -4.00453200 -0.00175700 -0.15301600<br>C -3.31237300 1.20049900 -0.00726300<br>C -1.94631700 1.20335100 0.27285300<br>C -1.25636700 -0.00652600 0.41271200<br>C -1.94430300 -1.20966200 0.26912900<br>C -3.31278500 -1.20240300 -0.01264900<br>H -5.06758500 0.02303000 -0.36685300<br>H -1.42015200 2.14661300 0.39150300<br>H -1.44024000 -2.16231900 0.37936600<br>C 1.07218800 -0.00545500 -0.62492700	Zero-point correction= 0.232301 (Hartree/Particle)<br>Thermal correction to Energy= 0.246243<br>Thermal correction to Enthalpy= 0.247187<br>Thermal correction to Gibbs Free Energy= 0.188544<br>Sum of electronic and zero-point Energies= -691.672723<br>Sum of electronic and thermal Energies= -691.658781<br>Sum of electronic and thermal Enthalpies= -691.657836<br>Sum of electronic and thermal Free Energies= -691.716480

H 0.79984500 0.87120200 -1.22064800<br>C 2.56083500 -0.00433100 -0.36055600<br>C 3.26712700 1.19658800 -0.23301000<br>C 3.26317400 -1.20445800 -0.20527800<br>C 4.63269600 1.20064000 0.04217600<br>H 2.74241100 2.13896200 -0.35815000<br>C 4.62860600 -1.20623600 0.07006300<br>H 2.73528600 -2.14763900 -0.30817500<br>C 5.31857200 -0.00228000 0.19556200<br>H 5.16204200 2.14302400 0.13168200<br>H 5.15477900 -2.14801100 0.18160900<br>H 6.38202500 -0.00162400 0.40650700<br>H 0.50445100 0.87077900 1.27373200<br>C 0.23246300 -0.00587100 0.67779000<br>O -4.03670500 2.35072700 -0.14760800<br>H -3.45311400 3.10568000 -0.02079700<br>O -3.92980200 -2.41437800 -0.13746800<br>H -4.86411300 -2.27606900 -0.32368600<br>H 0.50278700 -0.88386600 1.27167100<br>H 0.79974000 -0.88314500 -1.21866100	
**Name of radical**	**3-O3**
Cartesian Coordinates optimized at B3LYP/6-311G(d,p)	Energies at B3LYP/6-311G(d,p) in gas phase
C -4.07720000 -0.01777000 -0.15976900<br>C -3.36739100 1.15298100 -0.02157100<br>C -1.98309600 1.11478600 0.25731300<br>C -1.28706800 -0.10478200 0.40646300<br>C -1.98780600 -1.28363500 0.27381900<br>C -3.41215500 -1.29331500 -0.01666300<br>H -5.13838700 -0.00764900 -0.37142800<br>H -1.44141900 2.05094900 0.36950800<br>H -1.50878100 -2.24960900 0.38542700<br>C 1.04002800 0.00386000 -0.62785100<br>H 0.75560600 0.91089400 -1.17085700<br>C 2.52832900 0.01280800 -0.36055100<br>C 3.21860300 1.21537200 -0.17635100<br>C 3.24410200 -1.18507200 -0.26012200<br>C 4.58357900 1.22311200 0.10191600<br>H 2.68341600 2.15654500 -0.25965500<br>C 4.60883900 -1.18271200 0.01794100<br>H 2.72850400 -2.12888600 -0.40892300	Zero-point correction= 0.245714 (Hartree/Particle)<br>Thermal correction to Energy= 0.259852<br>Thermal correction to Enthalpy= 0.260796<br>Thermal correction to Gibbs Free Energy= 0.202623<br>Sum of electronic and zero-point Energies= -692.305922<br>Sum of electronic and thermal Energies= -692.291784<br>Sum of electronic and thermal Enthalpies= -692.290840<br>Sum of electronic and thermal Free Energies= -692.349013

C 5.28336300 0.02263700 0.20097700<br>H 5.10133500 2.16651600 0.23598100<br>H 5.14647000 -2.12197200 0.08632600<br>H 6.34627200 0.02640200 0.41413400<br>H 0.45904900 0.74723800 1.32395300<br>C 0.20167400 -0.09331800 0.67161000<br>O -4.03198600 2.33650600 -0.15762900<br>H -3.42010800 3.06926500 -0.03368800<br>O -4.03554900 -2.37231700 -0.13584200<br>H 0.48366500 -1.00282800 1.20864500<br>H 0.78186900 -0.84009500 -1.27461800	
**Name of radical**	**3-O5**
Cartesian Coordinates optimized at B3LYP/6-311G(d,p)	Energies at B3LYP/6-311G(d,p) in gas phase
C -4.07312900 -0.01693800 0.15711600<br>C -3.40738400 -1.29554600 0.01267600<br>C -1.98258300 -1.27826800 -0.27700100<br>C -1.28191800 -0.09514800 -0.40878100<br>C -1.97581300 1.11976800 -0.25931600<br>C -3.36480000 1.15044500 0.02123900<br>H -5.13675100 -0.03115300 0.36960100<br>H -1.50136300 -2.24316800 -0.38965600<br>H -1.46162300 2.06854300 -0.36455100<br>C 1.04378300 0.00645300 0.62654000<br>H 0.78509700 -0.83963800 1.27049500<br>C 2.53229900 0.01540400 0.36053900<br>C 3.25034300 -1.18180200 0.26955000<br>C 3.22038200 1.21802300 0.16803900<br>C 4.61539300 -1.17905200 -0.00733000<br>H 2.73623700 -2.12542800 0.42477800<br>C 4.58552000 1.22605200 -0.10916200<br>H 2.68309100 2.15858200 0.24353400<br>C 5.28774200 0.02616200 -0.19869900<br>H 5.15483100 -2.11781700 -0.06837400<br>H 5.10151200 2.16943900 -0.25000000<br>H 6.35083500 0.03039800 -0.41106500<br>H 0.48856400 -0.99248900 -1.21511300<br>C 0.20691200 -0.08502100 -0.67416900<br>O -4.02739700 -2.37398300 0.12992300<br>O -3.91136200 2.39360700 0.13652800<br>H -4.85327500 2.31156600 0.32179300	Zero-point correction= 0.232591 (Hartree/Particle)<br>Thermal correction to Energy= 0.246445<br>Thermal correction to Enthalpy= 0.247389<br>Thermal correction to Gibbs Free Energy= 0.188878<br>Sum of electronic and zero-point Energies= -691.674933<br>Sum of electronic and thermal Energies= -691.661079<br>Sum of electronic and thermal Enthalpies= -691.660135<br>Sum of electronic and thermal Free Energies= -691.718646

H	0.46373600	0.75888900	-1.32177000	
H	0.75869600	0.91208100	1.17126600	
**Name of anion**	**3-O3**			
Cartesian Coordinates optimized at B3LYP/6-311G(d,p)				Energies at B3LYP/6-311G(d,p) in gas phase
C	-4.04421200	-0.04454000	-0.17257100	Zero-point correction= 0.231116 (Hartree/Particle)
C	-3.35335000	1.13968400	-0.01604100	Thermal correction to Energy= 0.245193
C	-1.98204400	1.17325300	0.28189700	Thermal correction to Enthalpy= 0.246137
C	-1.30621000	-0.05378300	0.42667500	Thermal correction to Gibbs Free Energy= 0.187963
C	-1.98056100	-1.25308600	0.27529800	Sum of electronic and zero-point Energies= -691.749403
C	-3.39798600	-1.33424300	-0.03571100	Sum of electronic and thermal Energies= -691.735326
H	-5.10516900	-0.02463500	-0.40007800	Sum of electronic and thermal Enthalpies= -691.734382
H	-1.46281200	2.12069000	0.41736700	Sum of electronic and thermal Free Energies= -691.792556
H	-1.45775400	-2.20006800	0.39401800	
C	1.02725300	0.00413000	-0.60492300	
H	0.73998300	0.89811000	-1.16637100	
C	2.51643000	0.00826100	-0.35628000	
C	3.22612800	1.20768600	-0.21700900	
C	3.22900000	-1.19002100	-0.21787400	
C	4.59409300	1.21331000	0.04767500	
H	2.69444800	2.14864200	-0.32146500	
C	4.59659300	-1.19179700	0.04670800	
H	2.69908000	-2.13171400	-0.32236300	
C	5.28720600	0.01163400	0.18065200	
H	5.12025700	2.15757900	0.14721100	
H	5.12492300	-2.13480900	0.14565300	
H	6.35286500	0.01270400	0.38454500	
H	0.45681000	0.81180600	1.32171300	
C	0.18493600	-0.04846400	0.69721300	
O	-4.06161700	2.33355100	-0.15800700	
H	-3.42879500	3.04439000	-0.01959200	
O	-4.00531600	-2.42752400	-0.17095700	
H	0.46595000	-0.94658600	1.25797100	
H	0.75203200	-0.85648100	-1.22115600	
**Name of anion**	3-O5			
Cartesian Coordinates optimized at B3LYP/6-311G(d,p				Energies at B3LYP/6-311G(d,p) in gas phase
C	-4.02591200	-0.04024100	0.16623500	Zero-point correction= 0.231402 (Hartree/Particle)
C	-3.38833400	-1.33357300	0.02687500	Thermal correction to Energy= 0.245335
C	-1.97299500	-1.24984100	-0.28317500	Thermal correction to Enthalpy= 0.246280
C	-1.29974500	-0.04703200	-0.43002200	Thermal correction to Gibbs Free Energy= 0.188364
C	-1.96776500	1.18309000	-0.28345800	Sum of electronic and zero-point Energies= -691.751284

C	-3.33413800	1.14847100	0.01426100	Sum of electronic and thermal Energies= -691.737351
H	-5.09170900	-0.03691600	0.39520400	Sum of electronic and thermal Enthalpies= -691.736407
H	-1.44887600	-2.19559800	-0.40559700	Sum of electronic and thermal Free Energies= -691.794322
H	-1.46170600	2.13428500	-0.40744200	
C	1.03232100	0.01594400	0.60255500	
H	0.75584100	-0.83991700	1.22498200	
C	2.52184300	0.01636600	0.35585000	
C	3.23068800	1.21378800	0.19564500	
C	3.23560400	-1.18358600	0.24047700	
C	4.59901800	1.21551100	-0.06698700	
H	2.69780400	2.15587200	0.28150400	
C	4.60369100	-1.18917900	-0.02186500	
H	2.70618400	-2.12365000	0.36122600	
C	5.29346400	0.01216200	-0.17673800	
H	5.12445700	2.15826800	-0.18330700	
H	5.13293700	-2.13344400	-0.10276700	
H	6.35946100	0.01045600	-0.37902800	
H	0.47305000	-0.94721700	-1.25474900	
C	0.19202900	-0.04509400	-0.70032300	
O	-4.00325300	-2.42357100	0.15974600	
O	-3.98094800	2.37604300	0.14843400	
H	-4.90103800	2.17139000	0.34172900	
H	0.46365200	0.81140200	-1.32943300	
H	0.74493300	0.91457100	1.15626700	
**Name of cationic radical**				Energies at B3LYP/6-311G(d,p) in gas phase
C	-4.06680200	-0.02469200	-0.15840200	Zero-point correction= 0.245741 (Hartree/Particle)
C	-3.38194700	1.17653400	0.01633300	Thermal correction to Energy= 0.259804
C	-1.96637300	1.20633200	0.30118800	Thermal correction to Enthalpy= 0.260748
C	-1.22974300	0.03577000	0.41431800	Thermal correction to Gibbs Free Energy= 0.201834
C	-1.91961100	-1.17044800	0.23961400	Sum of electronic and zero-point Energies= -692.021416
C	-3.33713900	-1.19518200	-0.04642200	Sum of electronic and thermal Energies= -692.007354
H	-5.12881100	-0.01239800	-0.36872400	Sum of electronic and thermal Enthalpies= -692.006410
H	-1.48474700	2.17044700	0.43472100	Sum of electronic and thermal Free Energies= -692.065324
H	-1.41626400	-2.12697200	0.32239000	
C	1.08453100	-0.04231100	-0.63523000	
H	0.81321800	0.79236500	-1.28955700	
C	2.57202300	-0.02322800	-0.36232000	
C	3.27336100	1.18601900	-0.33446000	
C	3.26563600	-1.21024600	-0.10712700	
C	4.63729500	1.20995700	-0.05390600	

H	2.75460600	2.11602400	-0.54721600	
C	4.62948700	-1.18922200	0.17358000	
H	2.74150800	-2.16074500	-0.14090900	
C	5.31773800	0.02183400	0.20273400	
H	5.16945900	2.15403100	-0.04384500	
H	5.15572500	-2.11785000	0.36132000	
H	6.37986800	0.03859000	0.41635100	
H	0.52474300	0.96491700	1.20862500	
C	0.25402200	0.05094500	0.67470900	
O	-4.07704000	2.29449100	-0.08672600	
H	-3.53792000	3.08721600	0.04999200	
O	-3.83681000	-2.41278600	-0.18063500	
H	-4.78707600	-2.40644600	-0.37056500	
H	0.51837900	-0.78542800	1.32692400	
H	0.81325200	-0.95977700	-1.16826800	

**Name of compound (4)**	*trans*-Resveratrol			
Cartesian Coordinates optimized at B3LYP/6-311G(d,p)				Energies at B3LYP/6-311G(d,p) in gas phase
C	4.51009800	0.14910900	0.00005700	Zero-point correction= 0.226064 (Hartree/Particle)
C	3.96591700	-1.13397600	0.00043100	Thermal correction to Energy= 0.240201
C	2.58514700	-1.31738400	0.00028300	Thermal correction to Enthalpy= 0.241145
C	1.71762200	-0.21105400	-0.00012700	Thermal correction to Gibbs Free Energy= 0.184786
C	2.26706900	1.07784500	-0.00051200	Sum of electronic and zero-point Energies= -766.346047
C	3.64881400	1.24714000	-0.00043900	Sum of electronic and thermal Energies= -766.331910
H	5.58800300	0.26823800	0.00013300	Sum of electronic and thermal Enthalpies= -766.330966
H	2.17432900	-2.32274700	0.00038500	Sum of electronic and thermal Free Energies= -766.387325
H	1.64652900	1.96365200	-0.00097300	
C	-0.70793400	0.45265900	0.00019000	
H	-0.43243000	1.50407500	0.00071800	
C	-2.15054600	0.21222600	0.00007800	
C	-3.02444800	1.31030900	0.00121300	
C	-2.73224200	-1.07073300	-0.00114700	
C	-4.40530600	1.14862700	0.00119000	
H	-2.61205400	2.31385700	0.00215100	
C	-4.10489700	-1.24579300	-0.00118600	
H	-2.09997400	-1.95060500	-0.00214400	
C	-4.95399500	-0.13359600	-0.00000500	
H	-5.05514600	2.01912800	0.00209600	
H	-4.54467200	-2.23580700	-0.00215100	
H	0.00630000	-1.51977300	-0.00053200	

C 0.27415400 -0.46650700 -0.00020100	
O 4.84598200 -2.17945600 0.00087800	
H 4.35174100 -3.00551100 0.00222600	
O 4.11813100 2.53054700 -0.00086500	
H 5.08054500 2.51548300 -0.00083200	
O -6.29816800 -0.36807900 -0.00011000	
H -6.76746200 0.47266400 0.00075700	
**Name of radical**	**4-O3**
Cartesian Coordinates optimized at B3LYP/6-311G(d,p)	Energies at B3LYP/6-311G(d,p) in gas phase
C 4.58480800 0.19729100 0.01903900	Zero-point correction= 0.212719 (Hartree/Particle)
C 4.04515000 -1.06592700 0.08551400	Thermal correction to Energy= 0.227532
C 2.64532900 -1.24310500 0.05524000	Thermal correction to Enthalpy= 0.228476
C 1.74694200 -0.15238600 -0.03966100	Thermal correction to Gibbs Free Energy= 0.168231
C 2.28618800 1.12347500 -0.11892200	Sum of electronic and zero-point Energies= -765.712387
C 3.71697600 1.34918000 -0.08707400	Sum of electronic and thermal Energies= -765.697574
H 5.65526500 0.35324600 0.04068600	Sum of electronic and thermal Enthalpies= -765.696630
H 2.24153400 -2.25073900 0.11159700	Sum of electronic and thermal Free Energies= -765.756875
H 1.67054000 2.00811200 -0.21816800	
C -0.68236500 0.46534600 0.03273500	
H -0.41337600 1.51277200 0.14367000	
C -2.12174700 0.21459400 0.01331400	
C -3.00157900 1.28878200 0.21763600	
C -2.69326200 -1.05446900 -0.20365400	
C -4.38046000 1.11574100 0.21567200	
H -2.59530900 2.28096600 0.38356500	
C -4.06396000 -1.24046400 -0.20826000	
H -2.05540200 -1.91251700 -0.37955100	
C -4.91998100 -0.15325300 0.00340700	
H -5.03600800 1.96636500 0.37776600	
H -4.49801300 -2.21827200 -0.37795900	
H 0.05639000 -1.49419700 -0.13153300	
C 0.30868100 -0.44001100 -0.05556000	
O 4.89163500 -2.13173500 0.18070200	
H 4.38303700 -2.94794600 0.22344500	
O 4.18167500 2.51129300 -0.15396000	
O -6.26017900 -0.39830000 -0.01316500	
H -6.73803400 0.42338500 0.14131600	
**Name of radical**	**4-O5**
Cartesian Coordinates optimized at B3LYP/6-311G(d,p)	Energies at B3LYP/6-311G(d,p) in gas phase
C 4.57431300 0.12858700 -0.00007200	Zero-point correction= 0.213001 (Hartree/Particle)

C	4.05468600	-1.22411500	-0.00034000	Thermal correction to Energy= 0.227742
C	2.61306900	-1.38464000	-0.00020800	Thermal correction to Enthalpy= 0.228686
C	1.74941900	-0.30005100	0.00012600	Thermal correction to Gibbs Free Energy= 0.168247
C	2.31407400	0.99959500	0.00043300	Sum of electronic and zero-point Energies= -765.714986
C	3.71170400	1.19879300	0.00030300	Sum of electronic and thermal Energies= -765.700245
H	5.65229000	0.24887700	-0.00015100	Sum of electronic and thermal Enthalpies= -765.699301
H	2.24169500	-2.40275700	-0.00041200	Sum of electronic and thermal Free Energies= -765.759740
H	1.69087400	1.88438500	0.00085500	
C	-0.66231300	0.40697000	-0.00028900	
H	-0.37077100	1.45426000	-0.00087900	
C	-2.10835800	0.19346200	-0.00017700	
C	-2.96094300	1.30807900	-0.00084400	
C	-2.71185600	-1.07945800	0.00060400	
C	-4.34407400	1.17189300	-0.00073200	
H	-2.53047000	2.30401700	-0.00145700	
C	-4.08694100	-1.22903700	0.00071700	
H	-2.09588300	-1.97068800	0.00114600	
C	-4.91553600	-0.10082100	0.00004400	
H	-4.97807800	2.05378400	-0.00125600	
H	-4.54501400	-2.21057900	0.00132400	
H	0.02205000	-1.57984300	0.00067900	
C	0.30269600	-0.53073000	0.00021800	
O	4.81610400	-2.21500100	-0.00067000	
O	4.11724400	2.50101400	0.00061300	
H	5.07996300	2.53482200	0.00053500	
O	-6.26181400	-0.31147800	0.00021000	
H	-6.71857100	0.53627000	-0.00032100	
**Name of radical**	**4-O4'**			
Cartesian Coordinates optimized at B3LYP/6-311G(d,p)				Energies at B3LYP/6-311G(d,p) in gas phase
C	-4.43183700	0.15042400	0.00000000	Zero-point correction= 0.213311 (Hartree/Particle)
C	-3.89230900	-1.13665000	0.00000000	Thermal correction to Energy= 0.227976
C	-2.51332300	-1.32268900	0.00000100	Thermal correction to Enthalpy= 0.228920
C	-1.64488700	-0.21325800	0.00000000	Thermal correction to Gibbs Free Energy= 0.169561
C	-2.19105700	1.07947900	0.00000000	Sum of electronic and zero-point Energies= -765.722549
C	-3.57246300	1.25048800	0.00000100	Sum of electronic and thermal Energies= -765.707885
H	-5.50967200	0.26983400	0.00000000	Sum of electronic and thermal Enthalpies= -765.706941
H	-2.10196300	-2.32733900	0.00000300	Sum of electronic and thermal Free Energies= -765.766300
H	-1.56927700	1.96405200	0.00000000	
C	0.78223600	0.46453800	-0.00000100	
H	0.50163900	1.51383700	-0.00000200	

C	2.19198800	0.21846000	0.00000000	
C	3.08391800	1.33513000	-0.00000200	
C	2.76754400	-1.09308800	0.00000200	
C	4.43818600	1.17662300	-0.00000200	
H	2.65463200	2.33234700	-0.00000300	
C	4.11585500	-1.27686000	0.00000200	
H	2.11492100	-1.95838800	0.00000300	
C	5.04618900	-0.14946500	0.00000000	
H	5.11405000	2.02379100	-0.00000300	
H	4.55640300	-2.26719000	0.00000300	
H	0.06079300	-1.51884700	0.00000200	
C	-0.21250100	-0.46809500	0.00000100	
O	-4.77786100	-2.17366500	-0.00000100	
H	-4.29447100	-3.00632700	-0.00001700	
O	-4.03893000	2.53164700	0.00000100	
H	-5.00162700	2.52358400	0.00000200	
O	6.27545900	-0.31292900	0.00000000	

**Name of anion**	4-O3

Cartesian Coordinates optimized at B3LYP/6-311G(d,p				Energies at B3LYP/6-311G(d,p) in gas phase
C	4.55485800	0.16906700	0.00007900	Zero-point correction= 0.211456 (Hartree/Particle)
C	3.99275700	-1.09270000	-0.00019300	Thermal correction to Energy= 0.226519
C	2.60745300	-1.28472900	-0.00023900	Thermal correction to Enthalpy= 0.227463
C	1.76689400	-0.14492900	0.00000100	Thermal correction to Gibbs Free Energy= 0.167272
C	2.32206300	1.13225600	0.00028000	Sum of electronic and zero-point Energies= -765.792922
C	3.74936700	1.37619000	0.00032800	Sum of electronic and thermal Energies= -765.777859
H	5.63465100	0.27681100	0.00011400	Sum of electronic and thermal Enthalpies= -765.776915
H	2.18186700	-2.28572600	-0.00048500	Sum of electronic and thermal Free Energies= -765.837106
H	1.69357000	2.01719000	0.00048100	
C	-0.67477300	0.51456400	0.00005000	
H	-0.41084900	1.56849000	0.00018200	
C	-2.11082200	0.24284000	0.00000500	
C	-3.01783400	1.31783600	0.00012400	
C	-2.67541200	-1.05057700	-0.00015500	
C	-4.39651400	1.12431700	0.00008700	
H	-2.62718500	2.33030700	0.00024900	
C	-4.04699600	-1.25547400	-0.00019300	
H	-2.02383800	-1.91642900	-0.00025000	
C	-4.92044400	-0.16617700	-0.00007100	
H	-5.06499900	1.98255000	0.00018400	
H	-4.46196000	-2.25720900	-0.00031500	

H 0.05714200 -1.44811000 -0.00019700<br>C 0.32514900 -0.39175900 -0.00005300<br>O 4.85246100 -2.18910400 -0.00042600<br>H 4.29870500 -2.97549800 -0.00047900<br>O 4.24818200 2.52980300 0.00057000<br>O -6.27476100 -0.42279700 -0.00011400<br>H -6.73862300 0.42006000 -0.00001700	
**Name of anion**	4-O5
Cartesian Coordinates optimized at B3LYP/6-311G(d,p	Energies at B3LYP/6-311G(d,p) in gas phase
C 4.54447900 0.06460500 0.00008500<br>C 4.03081300 -1.28650200 0.00027000<br>C 2.58256600 -1.34619500 0.00024600<br>C 1.76173500 -0.21718600 0.00006300<br>C 2.32612900 1.07956200 -0.00011400<br>C 3.71514500 1.17827500 -0.00009700<br>H 5.62785000 0.18277200 0.00009200<br>H 2.14147000 -2.33980700 0.00038100<br>H 1.72732500 1.98067800 -0.00025600<br>C -0.66694700 0.48487800 -0.00008900<br>H -0.38945600 1.53565100 -0.00024000<br>C -2.10778200 0.23611800 -0.00008000<br>C -2.99922600 1.32359900 -0.00025300<br>C -2.69014000 -1.04939400 0.00009600<br>C -4.38081900 1.14972100 -0.00025300<br>H -2.59495000 2.33087200 -0.00039200<br>C -4.06432500 -1.23471300 0.00009800<br>H -2.05057500 -1.92395500 0.00023400<br>C -4.92252600 -0.13324500 -0.00007700<br>H -5.03741500 2.01714100 -0.00039000<br>H -4.49294800 -2.23061100 0.00023400<br>H 0.03745600 -1.49093200 0.00021700<br>C 0.31727700 -0.43886000 0.00006600<br>O 4.76124700 -2.31029700 0.00043600<br>O 4.25351500 2.46169800 -0.00027100<br>H 5.20947500 2.35329000 -0.00023400<br>O -6.28025600 -0.37104200 -0.00006700<br>H -6.73255700 0.47804000 -0.00019700	Zero-point correction= 0.211838 (Hartree/Particle)<br>Thermal correction to Energy= 0.226722<br>Thermal correction to Enthalpy= 0.227666<br>Thermal correction to Gibbs Free Energy= 0.167935<br>Sum of electronic and zero-point Energies= -765.795236<br>Sum of electronic and thermal Energies= -765.780353<br>Sum of electronic and thermal Enthalpies= -765.779408<br>Sum of electronic and thermal Free Energies= -765.839140
**Name of anion**	4-O4’
Cartesian Coordinates optimized at B3LYP/6-311G(d,p	Energies at B3LYP/6-311G(d,p) in gas phase
C -4.46934500 0.18138300 0.00001200	Zero-point correction= 0.212096 (Hartree/Particle)

C	-3.93637300	-1.10828000	0.00004500	Thermal correction to Energy= 0.226822
C	-2.56545300	-1.32002800	0.00000900	Thermal correction to Enthalpy= 0.227766
C	-1.65095600	-0.23711700	-0.00002000	Thermal correction to Gibbs Free Energy= 0.169216
C	-2.20007000	1.06628400	-0.00005700	Sum of electronic and zero-point Energies= -765.808758
C	-3.57339400	1.25574900	-0.00005800	Sum of electronic and thermal Energies= -765.794032
H	-5.54461300	0.32412800	0.00001700	Sum of electronic and thermal Enthalpies= -765.793088
H	-2.17972100	-2.33685800	-0.00005300	Sum of electronic and thermal Free Energies= -765.851638
H	-1.56600500	1.94289800	-0.00011900	
C	0.78235900	0.39788100	0.00002700	
H	0.49629500	1.44959400	0.00010900	
C	2.19481300	0.18663600	0.00000900	
C	3.07785400	1.30316300	0.00016600	
C	2.81469400	-1.09684200	-0.00016400	
C	4.44292800	1.17284100	0.00017000	
H	2.63685200	2.29945500	0.00029300	
C	4.17511600	-1.25147900	-0.00016600	
H	2.18505000	-1.98329800	-0.00031000	
C	5.10001500	-0.12396300	0.00000600	
H	5.09031900	2.04531200	0.00029400	
H	4.62529100	-2.24055000	-0.00030600	
H	0.02220900	-1.57587300	-0.00006700	
C	-0.23323200	-0.51930300	-0.00003100	
O	-4.83390100	-2.15472900	0.00011900	
H	-4.32657700	-2.97248500	0.00057500	
O	-4.03333000	2.55554300	-0.00012300	
H	-4.99468300	2.52924700	-0.00012200	
O	6.34246300	-0.26620300	0.00000400	
**Name of cationic radical**				Energies at B3LYP/6-311G(d,p) in gas phase
C	4.45789200	0.16338200	0.00001100	Zero-point correction= 0.227064 (Hartree/Particle)
C	3.93739900	-1.14613600	0.00000000	Thermal correction to Energy= 0.241799
C	2.56647500	-1.34842600	-0.00000500	Thermal correction to Enthalpy= 0.242743
C	1.69036800	-0.23528500	-0.00001200	Thermal correction to Gibbs Free Energy= 0.183660
C	2.22192500	1.07720700	-0.00000700	Sum of electronic and zero-point Energies= -766.094631
C	3.59833900	1.26871300	0.00000900	Sum of electronic and thermal Energies= -766.079896
H	5.53555200	0.28572000	0.00001600	Sum of electronic and thermal Enthalpies= -766.078952
H	2.15997400	-2.35343700	0.00001800	Sum of electronic and thermal Free Energies= -766.138034
H	1.58986500	1.95373600	-0.00001200	
C	-0.72059900	0.45700900	-0.00001200	
H	-0.43428700	1.50341000	-0.00001700	
C	-2.12029300	0.21138200	-0.00000700	

C	-3.00430500	1.32871300	-0.00001900	
C	-2.70074800	-1.09271500	0.00001200	
C	-4.36790600	1.16758100	-0.00001300	
H	-2.58614200	2.32864300	-0.00003300	
C	-4.05846000	-1.26423200	0.00001900	
H	-2.06682900	-1.96973500	0.00002300	
C	-4.91144400	-0.13469500	0.00000600	
H	-5.02585100	2.02993800	-0.00002300	
H	-4.50948000	-2.24825800	0.00003400	
H	0.00238400	-1.53982800	-0.00000700	
C	0.28283600	-0.49170400	-0.00001100	
O	4.85119100	-2.13624300	0.00001600	
H	4.42591900	-3.00178000	-0.00020500	
O	4.04432600	2.54243900	0.00001500	
H	5.00792000	2.57441000	0.00003400	
O	-6.21938600	-0.37709100	0.00001300	
H	-6.73694400	0.43958300	0.00000400	

**Name of compound (5)**	Astringin
Cartesian Coordinates optimized at B3LYP/6-311G(d,p)	Energies at B3LYP/6-311G(d,p) in gas phase
O -0.72741600 3.32295100 1.05886200	Zero-point correction= 0.403425 (Hartree/Particle)
C -0.36772200 2.18436500 0.39683200	Thermal correction to Energy= 0.431154
C 0.95228700 1.75423200 0.31748600	Thermal correction to Enthalpy= 0.432098
C -1.40309600 1.46968000 -0.21542600	Thermal correction to Gibbs Free Energy= 0.342785
C 1.27489200 0.58046500 -0.38536700	Sum of electronic and zero-point Energies= -1452.328102
H 1.73040300 2.34509200 0.78863700	Sum of electronic and thermal Energies= -1452.300372
C -1.08355300 0.30290900 -0.90062200	Sum of electronic and thermal Enthalpies= -1452.299428
H -2.41570800 1.84096600 -0.17722700	Sum of electronic and thermal Free Energies= -1452.388742
C 0.23629000 -0.14161100 -0.98880300	
H 0.44312800 -1.04260000 -1.55393500	
O -2.01382700 -0.45886100 -1.58497100	
C 3.74780900 0.56891500 0.06969300	
H 3.64688300 1.42511700 0.73158700	
C 2.64329300 0.07384800 -0.51668500	
H 2.73532200 -0.79933200 -1.15659600	
C 5.12003000 0.07651500 -0.06437000	
C 6.12128700 0.66536100 0.72825200	
C 5.49346000 -0.95032300 -0.94601500	
C 7.44185600 0.24812300 0.65702900	
H 5.87826900 1.46373800 1.42013200	

C	6.81560500	-1.37372600	-1.02320300	
H	4.75749900	-1.41983000	-1.58592100	
C	7.79022200	-0.78267000	-0.22737400	
O	9.12123600	-1.12766000	-0.23077600	
H	9.27846600	-1.83431600	-0.86423200	
C	-3.19112700	-1.35051500	0.38563900	
C	-4.60061300	-1.64956300	0.87962400	
C	-5.47786100	-0.41193700	0.76556500	
C	-5.44913500	0.12256200	-0.67205800	
C	-3.26912500	-0.75779000	-1.03036600	
H	-5.03005600	-2.44228500	0.24908400	
H	-2.72723300	-0.62307800	1.06110700	
H	-5.86775400	-0.63853100	-1.34653700	
H	-3.68465100	-1.50820100	-1.71434200	
H	-5.07487600	0.36334200	1.43273900	
O	-4.09033100	0.40065500	-1.03103500	
H	-7.82072000	0.66390600	-0.00300600	
C	-6.25397200	1.41017200	-0.85401700	
H	-6.02684000	1.82190200	-1.83900300	
H	-5.93592100	2.14590200	-0.09991700	
O	-7.64775600	1.17058700	-0.80747900	
O	-6.82653800	-0.70937100	1.11983500	
H	-6.80081700	-1.15636900	1.97451500	
O	-4.61428600	-2.05494500	2.24316800	
H	-4.02108000	-2.81123300	2.31811600	
O	-2.48114400	-2.58460300	0.40652500	
H	-1.55008500	-2.39886600	0.24424600	
H	0.06096400	3.74670700	1.41353900	
H	7.09399200	-2.16681200	-1.71119600	
O	8.38653500	0.83800200	1.44023400	
H	9.23408200	0.41667100	1.24842000	
**Name of radical**	**5-O3**			
Cartesian Coordinates optimized at B3LYP/6-311G(d,p)				Energies at B3LYP/6-311G(d,p) in gas phase
O	-0.61687300	3.01607800	1.40478500	Zero-point correction= 0.390163 (Hartree/Particle)
C	-0.33566700	1.98346700	0.75522500	Thermal correction to Energy= 0.417671
C	1.04010700	1.58455600	0.55529500	Thermal correction to Enthalpy= 0.418615
C	-1.38271800	1.16178400	0.17508000	Thermal correction to Gibbs Free Energy= 0.329070
C	1.37107000	0.44181000	-0.16371400	Sum of electronic and zero-point Energies= -1451.694915
H	1.78883300	2.23605900	0.98715900	Sum of electronic and thermal Energies= -1451.667406
C	-1.04501600	0.03020500	-0.52436200	Sum of electronic and thermal Enthalpies= -1451.666462

H	-2.40133600	1.49832600	0.28372400	Sum of electronic and thermal Free Energies= -1451.756007
C	0.31375600	-0.32562800	-0.69826900	
H	0.52558300	-1.21970100	-1.27349700	
O	-1.91294500	-0.84208200	-1.14421600	
C	3.85958900	0.51218800	0.14025800	
H	3.75637400	1.34509000	0.83083500	
C	2.74553400	-0.01350400	-0.39910300	
H	2.83150100	-0.86596600	-1.06701700	
C	5.24086200	0.08252000	-0.07989400	
C	6.25610200	0.69042800	0.67982600	
C	5.60784100	-0.90285500	-1.01008600	
C	7.58662700	0.32942600	0.53068700	
H	6.01634700	1.45843100	1.40621300	
C	6.93975900	-1.26934500	-1.16580600	
H	4.85916600	-1.38370900	-1.62646900	
C	7.92919400	-0.66153100	-0.40098800	
O	9.26960500	-0.95213600	-0.47997800	
H	9.42379300	-1.63293400	-1.14196800	
C	-3.52292800	-1.38130500	0.63795300	
C	-5.02341400	-1.43368800	0.90129900	
C	-5.68090400	-0.11514200	0.52032100	
C	-5.34790800	0.23632600	-0.93479000	
C	-3.28180000	-0.92706000	-0.81152900	
H	-5.45597900	-2.23116300	0.27919900	
H	-3.06635900	-0.67038800	1.33543400	
H	-5.75114400	-0.54368300	-1.59647500	
H	-3.65840100	-1.70254600	-1.49029300	
H	-5.28112400	0.67658900	1.16994300	
O	-3.92145500	0.30433700	-1.07310600	
H	-7.70316300	1.14320700	-0.76256100	
C	-5.92472000	1.58099800	-1.38040200	
H	-5.47774800	1.84625100	-2.34006600	
H	-5.64379800	2.35626400	-0.65208300	
O	-7.32450000	1.51181200	-1.57149000	
O	-7.09644400	-0.19448700	0.66010700	
H	-7.27402300	-0.53193700	1.54648400	
O	-5.31698000	-1.67320500	2.27161400	
H	-4.88692500	-2.50060900	2.51570000	
O	-3.02786400	-2.69743000	0.86110900	
H	-2.06823200	-2.67830400	0.78666800	

H	7.21435500	-2.03048600	-1.89030300	
O	8.54517400	0.93419100	1.28335700	
H	9.39832200	0.55270200	1.03991700	

**Name of radical**	**5-O3’**
Cartesian Coordinates optimized at B3LYP/6-311G(d,p)	Energies at B3LYP/6-311G(d,p) in gas phase

O	-0.70499000	3.29042200	1.11803300	Zero-point correction= 0.391763 (Hartree/Particle)
C	-0.32650400	2.15948800	0.45648000	Thermal correction to Energy= 0.418755
C	0.99900900	1.74681300	0.37892400	Thermal correction to Enthalpy= 0.419699
C	-1.35105400	1.43040100	-0.15830200	Thermal correction to Gibbs Free Energy= 0.331471
C	1.33393900	0.57817800	-0.32683200	Sum of electronic and zero-point Energies= -1451.711841
H	1.76891800	2.34454900	0.85432900	Sum of electronic and thermal Energies= -1451.684848
C	-1.01904600	0.26777900	-0.84474200	Sum of electronic and thermal Enthalpies= -1451.683904
H	-2.36772800	1.79027800	-0.12074800	Sum of electronic and thermal Free Energies= -1451.772132
C	0.30775800	-0.15751200	-0.93321700	
H	0.52722700	-1.05598400	-1.49733900	
O	-1.93400300	-0.50838800	-1.52846900	
C	3.80840800	0.60120700	0.12204300	
H	3.70485400	1.46869300	0.76747700	
C	2.70694500	0.08654500	-0.45669000	
H	2.80408600	-0.79073300	-1.08938300	
C	5.18026200	0.11638400	-0.00494000	
C	6.18637200	0.74801700	0.70136800	
C	5.51961200	-0.99887400	-0.84470200	
C	7.54917600	0.31457600	0.61812800	
H	5.97601300	1.59405500	1.34544000	
C	6.81552900	-1.46478200	-0.96809300	
H	4.73866500	-1.49443300	-1.40683800	
C	7.82946200	-0.83122200	-0.25439900	
O	9.09945600	-1.21180600	-0.31286600	
H	9.56326700	-0.58514100	0.28638700	
C	-3.18360000	-1.36700100	0.41208100	
C	-4.61147200	-1.63272300	0.87270500	
C	-5.46213200	-0.38009300	0.72272100	
C	-5.38538400	0.13766900	-0.71903400	
C	-3.21248600	-0.78076700	-1.00864000	
H	-5.03949800	-2.42374200	0.23921000	
H	-2.72055000	-0.64733800	1.09672400	
H	-5.79578400	-0.62418300	-1.39741000	
H	-3.61813200	-1.52951100	-1.70007700	
H	-5.06255300	0.39524400	1.39201500	

O	-4.01168200	0.39034300	-1.04183100	
H	-7.76923700	0.72366700	-0.12779000	
C	-6.16505700	1.43505600	-0.93803400	
H	-5.90099500	1.83387500	-1.91910200	
H	-5.86067400	2.17353600	-0.18097600	
O	-7.56233100	1.21485000	-0.93386300	
O	-6.82462800	-0.64848800	1.04328400	
H	-6.83135700	-1.08511800	1.90368600	
O	-4.66738700	-2.02231900	2.23933200	
H	-4.10172800	-2.79693800	2.33603900	
O	-2.50339700	-2.61696400	0.45568900	
H	-1.56655600	-2.45714200	0.30071700	
H	0.07405100	3.72897300	1.47557800	
H	7.05415100	-2.30620500	-1.60706100	
O	8.52210400	0.82344800	1.22171200	
**Name of radical**	**5-O4'**			
Cartesian Coordinates optimized at B3LYP/6-311G(d,p)				Energies at B3LYP/6-311G(d,p) in gas phase
O	-0.67450600	3.27203500	1.10226400	Zero-point correction= 0.391719 (Hartree/Particle)
C	-0.30326400	2.13715800	0.44451900	Thermal correction to Energy= 0.418678
C	1.01854500	1.71545000	0.36609300	Thermal correction to Enthalpy= 0.419622
C	-1.33432900	1.41192900	-0.16547100	Thermal correction to Gibbs Free Energy= 0.331603
C	1.34414800	0.54065500	-0.33793100	Sum of electronic and zero-point Energies= -1451.717372
H	1.79310700	2.30821900	0.83974400	Sum of electronic and thermal Energies= -1451.690413
C	-1.01350200	0.24331900	-0.84870200	Sum of electronic and thermal Enthalpies= -1451.689469
H	-2.34840300	1.77893900	-0.12619100	Sum of electronic and thermal Free Energies= -1451.777489
C	0.30928400	-0.19104000	-0.93946200	
H	0.52175300	-1.09304900	-1.50042400	
O	-1.93584600	-0.52811000	-1.52579000	
C	3.82509700	0.56476100	0.09371200	
H	3.72387200	1.44775400	0.71823200	
C	2.70602000	0.03962500	-0.46934000	
H	2.79737000	-0.85049200	-1.08378300	
C	5.17288600	0.07507900	-0.03279100	
C	6.20442700	0.75122400	0.65589200	
C	5.50890800	-1.07219900	-0.83039400	
C	7.50483300	0.31256500	0.56299900	
H	5.97864600	1.61968200	1.26413300	
C	6.78970100	-1.52422600	-0.93784200	
H	4.72218700	-1.59103500	-1.36369600	
C	7.86883000	-0.85924400	-0.24575300	

O	9.06845100	-1.19502200	-0.27938400	
C	-3.19356300	-1.36879300	0.41758800	
C	-4.62389100	-1.61551600	0.88170200	
C	-5.46016200	-0.35343700	0.72915400	
C	-5.37938600	0.15897800	-0.71422600	
C	-3.21774500	-0.78469600	-1.00411800	
H	-5.06233800	-2.40344400	0.25155700	
H	-2.72024000	-0.65392700	1.10042900	
H	-5.79848900	-0.60028800	-1.39004800	
H	-3.63177000	-1.53018900	-1.69396600	
H	-5.05062200	0.41928100	1.39551100	
O	-4.00285500	0.39532500	-1.03894700	
H	-7.75694800	0.77323000	-0.12392800	
C	-6.14504200	1.46412000	-0.93668100	
H	-5.87679400	1.85741200	-1.91885000	
H	-5.83282600	2.20137900	-0.18159300	
O	-7.54437900	1.25863000	-0.93202400	
O	-6.82508100	-0.60487400	1.05214500	
H	-6.83609600	-1.03882300	1.91386900	
O	-4.68169400	-1.99962800	2.24964900	
H	-4.12904500	-2.78343000	2.34748600	
O	-2.53000600	-2.62741100	0.46215600	
H	-1.59163800	-2.48165200	0.30301200	
H	0.10687100	3.70909700	1.45660100	
H	7.04398900	-2.38811500	-1.54007800	
O	8.51981700	0.90961200	1.19224000	
H	9.30543200	0.38072000	0.93983500	

**Name of anion**	5-O3

Cartesian Coordinates optimized at B3LYP/6-311G(d,p				Energies at B3LYP/6-311G(d,p) in gas phase
O	-0.63192400	3.60119800	1.16057200	Zero-point correction= 0.390221 (Hartree/Particle)
C	-0.35686700	2.50508700	0.38272500	Thermal correction to Energy= 0.416907
C	0.92133000	1.96815100	0.30159600	Thermal correction to Enthalpy= 0.417852
C	-1.43109100	1.95427900	-0.32630300	Thermal correction to Gibbs Free Energy= 0.330989
C	1.19457600	0.84603700	-0.51152200	Sum of electronic and zero-point Energies= -1451.802386
H	1.72272000	2.43440900	0.86559600	Sum of electronic and thermal Energies= -1451.775700
C	-1.16744000	0.84498300	-1.12158500	Sum of electronic and thermal Enthalpies= -1451.774756
H	-2.42013500	2.38414900	-0.26754100	Sum of electronic and thermal Free Energies= -1451.861618
C	0.10526200	0.29365800	-1.22328100	
H	0.26154900	-0.53857400	-1.90110600	
O	-2.17348100	0.25291100	-1.88867100	

C	3.63478900	0.60654800	0.02043700	
H	3.55667000	1.43648200	0.72156000	
C	2.50641100	0.23992200	-0.64202000	
H	2.55492700	-0.59408300	-1.33640400	
C	4.96467700	0.04306600	-0.05484300	
C	5.96994300	0.59723700	0.78064800	
C	5.28676400	-1.02408500	-0.91561900	
C	7.29450700	0.11899600	0.79052400	
H	5.72198700	1.42122100	1.44469900	
C	6.58947200	-1.53208700	-0.94200900	
H	4.53368300	-1.45535500	-1.56489700	
C	7.55767100	-0.98188500	-0.11886700	
O	8.84185000	-1.40104300	-0.07097100	
H	9.18428100	-0.76514400	0.61900100	
C	-2.81072000	-1.19063600	0.01021300	
C	-4.04179700	-1.86044100	0.60295600	
C	-5.11954200	-0.82738200	0.88385100	
C	-5.42664200	-0.03494400	-0.39375200	
C	-3.22442800	-0.39452000	-1.23907000	
H	-4.43301400	-2.58642300	-0.12635400	
H	-2.39617400	-0.49507500	0.74750400	
H	-5.83199600	-0.72548600	-1.14937800	
H	-3.64496500	-1.07494200	-1.99128100	
H	-4.74363200	-0.12882500	1.64420100	
O	-4.22113900	0.56434000	-0.86718900	
H	-7.67760700	-0.11589400	0.71397900	
C	-6.44875800	1.08080600	-0.17627100	
H	-6.48286700	1.69792100	-1.07606400	
H	-6.11469100	1.71852600	0.65586400	
O	-7.75489000	0.57136900	0.03862700	
O	-6.31847600	-1.45374200	1.34839200	
H	-6.05855400	-2.04984600	2.06099900	
O	-3.74745900	-2.52367000	1.82774500	
H	-2.95783800	-3.05369900	1.66331200	
O	-1.88151000	-2.22781200	-0.28163900	
H	-1.01441700	-1.81659000	-0.39553700	
H	0.18629000	3.88662400	1.58009200	
H	6.85594200	-2.35359500	-1.60076000	
O	8.28817100	0.52363000	1.48884400	

**Name of anion**	5-O3’

Cartesian Coordinates optimized at B3LYP/6-311G(d,p				Energies at B3LYP/6-311G(d,p) in gas phase
O	-0.64584800	3.72209700	1.27581900	Zero-point correction= 0.389552 (Hartree/Particle)
C	-0.43262100	2.72066500	0.55546600	Thermal correction to Energy= 0.416845
C	0.86180600	2.07391700	0.46165800	Thermal correction to Enthalpy= 0.417789
C	-1.46920100	2.10008000	-0.25782400	Thermal correction to Gibbs Free Energy= 0.329454
C	1.11140400	0.97088700	-0.34533800	Sum of electronic and zero-point Energies= -1451.790191
H	1.64767400	2.51555300	1.06569200	Sum of electronic and thermal Energies= -1451.762899
C	-1.20397200	0.99973700	-1.04071200	Sum of electronic and thermal Enthalpies= -1451.761955
H	-2.46288800	2.53090700	-0.23890900	Sum of electronic and thermal Free Energies= -1451.850290
C	0.05836200	0.39557800	-1.10523700	
H	0.24700000	-0.38666100	-1.83251000	
O	-2.21122100	0.41701500	-1.84666100	
C	3.58405200	0.72453300	0.11871300	
H	3.57571800	1.61045000	0.74707600	
C	2.43015700	0.34080300	-0.46113900	
H	2.44433500	-0.53877300	-1.10317500	
C	4.88934700	0.07300600	-0.00668100	
C	6.00193100	0.65626100	0.62960500	
C	5.10593600	-1.11534700	-0.72829800	
C	7.26721700	0.09235400	0.55209400	
H	5.88591900	1.57133900	1.19930900	
C	6.37249400	-1.68446400	-0.81013300	
H	4.28021100	-1.60534800	-1.22794500	
C	7.45464900	-1.08791800	-0.17502000	
O	8.75093300	-1.58000500	-0.19826200	
H	8.77043100	-2.39040800	-0.71471500	
C	-2.78948400	-1.03857900	0.06262700	
C	-3.97599700	-1.81476200	0.61067800	
C	-5.15196800	-0.88166200	0.83208600	
C	-5.47748700	-0.14281000	-0.47387800	
C	-3.21321800	-0.31416500	-1.22689500	
H	-4.26918700	-2.58055600	-0.12442800	
H	-2.48413700	-0.28237700	0.79317100	
H	-5.79479200	-0.88439300	-1.22522500	
H	-3.55119100	-1.04962200	-1.97217400	
H	-4.87290400	-0.13959100	1.59198700	
O	-4.31967800	0.55388100	-0.91667700	
H	-7.73862600	-0.40954900	0.56690300	
C	-6.60224200	0.88045800	-0.31409000	
H	-6.66561100	1.47014700	-1.23052300	

H	-6.34896900	1.56511500	0.50839900	
O	-7.86645700	0.26158500	-0.11711800	
O	-6.30823300	-1.61062300	1.26739200	
H	-6.01798300	-2.16165800	2.00384900	
O	-3.66700000	-2.44556300	1.85199500	
H	-2.78142800	-2.81354400	1.73752600	
O	-1.74341600	-1.98097800	-0.14663400	
H	-0.93277100	-1.45677700	-0.27780400	
H	6.52052800	-2.60360800	-1.37194600	
O	8.32578500	0.68818800	1.18391800	
H	9.10276000	0.14313100	1.00918000	

**Name of anion**	5-O4’

Cartesian Coordinates optimized at B3LYP/6-311G(d,p				Energies at B3LYP/6-311G(d,p) in gas phase
O	-0.61531300	3.60499500	1.15642100	Zero-point correction= 0.390682 (Hartree/Particle)
C	-0.34931200	2.50402700	0.37877200	Thermal correction to Energy= 0.417307
C	0.92346100	1.95667300	0.30321100	Thermal correction to Enthalpy= 0.418251
C	-1.42936400	1.96370100	-0.33082500	Thermal correction to Gibbs Free Energy= 0.332020
C	1.19563200	0.82839800	-0.50946500	Sum of electronic and zero-point Energies= -1451.811138
H	1.72455400	2.41833400	0.87190000	Sum of electronic and thermal Energies= -1451.784513
C	-1.17006300	0.85020400	-1.12331100	Sum of electronic and thermal Enthalpies= -1451.783569
H	-2.41461800	2.40223700	-0.27552500	Sum of electronic and thermal Free Energies= -1451.869801
C	0.09547900	0.28551900	-1.22137300	
H	0.24449100	-0.54600000	-1.90200100	
O	-2.18294000	0.26511000	-1.89099300	
C	3.64001700	0.59464600	0.01127200	
H	3.56422200	1.44344100	0.69057500	
C	2.49499900	0.21638800	-0.63776900	
H	2.53587000	-0.63108900	-1.31644800	
C	4.95269300	0.03553000	-0.05685700	
C	5.99487700	0.60520800	0.74254600	
C	5.29483400	-1.06865800	-0.87620500	
C	7.26240900	0.09740400	0.71228300	
H	5.77672100	1.45399200	1.38553400	
C	6.57259000	-1.58823300	-0.91388000	
H	4.52789800	-1.52196000	-1.49702800	
C	7.63043900	-1.03732900	-0.12242000	
O	8.83067000	-1.43506600	-0.08081100	
C	-2.80964000	-1.17057400	0.01712000	
C	-4.03533700	-1.84806100	0.61146800	
C	-5.12162700	-0.82261400	0.88585500	

C	-5.43417300	-0.04050800	-0.39699200	
C	-3.22755200	-0.38706600	-1.23876400	
H	-4.41985900	-2.58098200	-0.11450900	
H	-2.40404500	-0.46453700	0.74937700	
H	-5.83396300	-0.73957900	-1.14796500	
H	-3.64393900	-1.07599100	-1.98589100	
H	-4.75220300	-0.11638000	1.64219500	
O	-4.23443400	0.56597100	-0.87352000	
H	-7.68262300	-0.13437800	0.71195000	
C	-6.46546400	1.06810100	-0.18633400	
H	-6.50570900	1.67849900	-1.09045600	
H	-6.13529600	1.71442300	0.64068000	
O	-7.76756900	0.54995000	0.03450700	
O	-6.31585800	-1.45648300	1.35410500	
H	-6.05016100	-2.04761700	2.06868400	
O	-3.73595400	-2.50383200	1.83952000	
H	-2.93428400	-3.01699200	1.67929900	
O	-1.86871800	-2.20000300	-0.26367400	
H	-1.00805700	-1.77582200	-0.38579700	
H	0.20688500	3.88114800	1.57405700	
H	6.81473700	-2.43472600	-1.54902900	
O	8.29962800	0.59080400	1.44844200	
H	9.02389700	-0.01412300	1.16841700	
**Name of cationic radical**				Energies at B3LYP/6-311G(d,p) in gas phase
O	-0.72045300	2.78493700	1.64439700	Zero-point correction= 0.403876 (Hartree/Particle)
C	-0.28413600	1.69393400	0.97901200	Thermal correction to Energy= 0.431339
C	1.04929600	1.37170900	0.81008600	Thermal correction to Enthalpy= 0.432283
C	-1.30509800	0.87885600	0.44126500	Thermal correction to Gibbs Free Energy= 0.343849
C	1.39002400	0.20341300	0.08315500	Sum of electronic and zero-point Energies= -1452.076365
H	1.81452900	2.01573300	1.22457700	Sum of electronic and thermal Energies= -1452.048903
C	-0.97720900	-0.28430700	-0.26412400	Sum of electronic and thermal Enthalpies= -1452.047959
H	-2.32852900	1.19936300	0.55336700	Sum of electronic and thermal Free Energies= -1452.136392
C	0.36573700	-0.61526500	-0.44144000	
H	0.60759300	-1.50773400	-1.00432600	
O	-1.85593500	-1.14769700	-0.83181900	
C	3.88128800	0.44919500	0.24411500	
H	3.77285500	1.36313300	0.81847800	
C	2.74100900	-0.20381300	-0.16472400	
H	2.84392600	-1.11795400	-0.73947600	
C	5.22799700	0.05264400	-0.01247300	

C	6.27648900	0.85984000	0.48677300	
C	5.56353300	-1.12168600	-0.75407900	
C	7.59720800	0.53205300	0.26834300	
H	6.05912300	1.75724400	1.05271300	
C	6.87764500	-1.45998800	-0.97858800	
H	4.78633200	-1.76020400	-1.15045300	
C	7.90284000	-0.64595500	-0.47593300	
O	9.21265300	-0.88028600	-0.63585700	
H	9.38606500	-1.68295200	-1.14468900	
C	-3.76962500	-1.48050500	0.68719600	
C	-5.28807900	-1.31806700	0.74283000	
C	-5.70108800	0.07786900	0.29368800	
C	-5.12332800	0.38530800	-1.09294700	
C	-3.27577600	-1.05119200	-0.70301200	
H	-5.73704900	-2.05162600	0.05806700	
H	-3.32237800	-0.84388200	1.46153900	
H	-5.52310700	-0.32899500	-1.82481600	
H	-3.63438200	-1.77230800	-1.44645900	
H	-5.29460200	0.80794600	1.00984900	
O	-3.68722300	0.25156200	-1.02461200	
H	-7.33939500	1.61820900	-1.26891400	
C	-5.43984200	1.79843900	-1.58604700	
H	-4.81288300	2.00905700	-2.45451400	
H	-5.18367600	2.52466600	-0.79940300	
O	-6.78254800	1.91230000	-2.00170000	
O	-7.11399300	0.19452100	0.22414400	
H	-7.47124800	-0.10565000	1.06915000	
O	-5.79159700	-1.49627200	2.05681900	
H	-5.61561000	-2.40579600	2.32269300	
O	-3.50035000	-2.85150900	0.93652700	
H	-2.54927400	-2.97763500	1.01246400	
H	0.01923900	3.31072000	1.96962900	
H	7.12984300	-2.35156700	-1.54231600	
O	8.57674400	1.30985800	0.74885700	
H	9.44093400	0.94603400	0.51022800	

**Name of compound (6)**	Isorhapontin
Cartesian Coordinates optimized at B3LYP/6-311G(d,p)	Energies at B3LYP/6-311G(d,p) in gas phase
C -3.89490700 -1.83338600 -0.29088200	Zero-point correction= 0.431856 (Hartree/Particle)
C -5.38709700 -1.75994500 -0.57925300	Thermal correction to Energy= 0.459930

C	-6.06835100	-0.82180000	0.40577800	Thermal correction to Enthalpy= 0.460874
C	-5.38175100	0.55040000	0.39340400	Thermal correction to Gibbs Free Energy= 0.371960
C	-3.30887000	-0.42095500	-0.24840100	Sum of electronic and zero-point Energies= -1491.616740
H	-5.52860000	-1.36413400	-1.59579000	Sum of electronic and thermal Energies= -1491.588666
H	-3.73661800	-2.28688000	0.69616000	Sum of electronic and thermal Enthalpies= -1491.587722
H	-5.49305100	0.99933500	-0.60460000	Sum of electronic and thermal Free Energies= -1491.676636
H	-3.36668000	0.04792500	-1.24305400	
H	-5.97161900	-1.24718400	1.41473100	
O	-3.99413100	0.36998500	0.70632300	
H	-7.78011400	1.20890400	0.82608100	
C	-5.96693800	1.52229200	1.41935000	
H	-5.31164800	2.39279300	1.48568400	
H	-5.98128400	1.03744500	2.40686600	
O	-7.24662300	1.98736200	1.03405700	
O	-7.44503500	-0.65102000	0.07633300	
H	-7.82045300	-1.53387600	-0.02651000	
O	-6.02011200	-3.02880500	-0.46085600	
H	-5.56106200	-3.63207100	-1.05624200	
O	-3.30981900	-2.62335200	-1.31668500	
H	-2.36898500	-2.69798600	-1.12271700	
O	-1.97070500	-0.55767600	0.14216800	
C	-1.07578400	0.46523500	-0.10396400	
C	0.26700400	0.11352200	-0.00729900	
C	-1.46964400	1.76414200	-0.41591900	
C	1.26349600	1.07082800	-0.23700400	
H	0.50809600	-0.90861400	0.25136300	
C	-0.47696100	2.71591100	-0.65388100	
H	-2.50627100	2.06497800	-0.43420300	
C	0.87116200	2.37979400	-0.56684400	
H	1.62854400	3.13621300	-0.75173500	
O	-0.90044400	3.97778200	-0.95996500	
H	-0.13420700	4.54825700	-1.07834200	
C	3.25945300	-0.42503100	0.08254600	
H	2.60791600	-1.28240400	0.22953300	
C	2.69607500	0.77312800	-0.15818400	
H	3.33992000	1.63271700	-0.32527900	
C	4.68312300	-0.74306200	0.16537500	
C	5.07071500	-2.07715800	0.34309000	
C	5.69780000	0.23492400	0.07284400	
C	6.41232500	-2.44359500	0.42015600	

H 4.30802100 -2.84410300 0.41962100<br>C 7.03037200 -0.12599300 0.14991700<br>H 5.43108800 1.27509700 -0.05321400<br>C 7.40154400 -1.47738100 0.32383400<br>H 6.70814200 -3.47663400 0.55617500<br>O 8.71291500 -1.82029300 0.39920600<br>H 9.22707100 -1.00595800 0.31317200<br>C 7.86066700 2.11635300 -0.09303700<br>H 8.83939600 2.59187900 -0.12473100<br>H 7.28600600 2.52175500 0.74641300<br>H 7.32961500 2.31372100 -1.03027900<br>O 8.10472800 0.72595200 0.07581100	
**Name of radical**	**6-O3**
Cartesian Coordinates optimized at B3LYP/6-311G(d,p)	Energies at B3LYP/6-311G(d,p) in gas phase
C -3.91146900 -1.77042600 -0.53062500<br>C -5.42274000 -1.67477400 -0.68822300<br>C -6.02043700 -0.84749900 0.44062900<br>C -5.33564300 0.52356600 0.51512900<br>C -3.32439200 -0.36546100 -0.38538500<br>H -5.64465400 -1.17633200 -1.64329000<br>H -3.67646800 -2.32604700 0.38627800<br>H -5.52550400 1.07338000 -0.41809300<br>H -3.45306500 0.20018000 -1.32141900<br>H -5.84293800 -1.37427800 1.38900700<br>O -3.92603300 0.31858800 0.69362400<br>H -7.68912800 1.11707400 1.21390400<br>C -5.83144400 1.38026600 1.68134400<br>H -5.17137200 2.24365100 1.78265300<br>H -5.76487200 0.79561800 2.61099900<br>O -7.13705400 1.87341400 1.45231800<br>O -7.41795100 -0.65223800 0.24238400<br>H -7.80373400 -1.52183900 0.08122500<br>O -6.04630800 -2.95239300 -0.65071900<br>H -5.65084000 -3.48403400 -1.35075400<br>O -3.41445700 -2.44219100 -1.67956100<br>H -2.46623900 -2.56293600 -1.56113500<br>O -1.95567300 -0.52930300 -0.11144700<br>C -1.10417400 0.54317700 -0.25497100<br>C 0.26026800 0.20442000 -0.15585800	Zero-point correction= 0.418854 (Hartree/Particle)<br>Thermal correction to Energy= 0.447528<br>Thermal correction to Enthalpy= 0.448472<br>Thermal correction to Gibbs Free Energy= 0.356129<br>Sum of electronic and zero-point Energies= -1490.985156<br>Sum of electronic and thermal Energies= -1490.956482<br>Sum of electronic and thermal Enthalpies= -1490.955538<br>Sum of electronic and thermal Free Energies= -1491.047880

C	-1.49776800	1.84090600	-0.47561700
C	1.28196200	1.17748400	-0.29017100
H	0.49785900	-0.83570600	0.02518600
C	-0.49213100	2.87468700	-0.64216600
H	-2.53232100	2.14918600	-0.50568100
C	0.89915000	2.48573600	-0.54048900
H	1.63120700	3.27572200	-0.66124100
O	-0.81959900	4.06116100	-0.85978400
C	3.23041800	-0.35153700	0.13617900
H	2.55543800	-1.17749000	0.34521500
C	2.70414700	0.84601400	-0.18208500
H	3.36807500	1.68361100	-0.37512000
C	4.64270900	-0.70313400	0.25387800
C	4.98637200	-2.00433900	0.64144500
C	5.68729900	0.21154400	-0.00523400
C	6.31544100	-2.39818400	0.77436400
H	4.19948300	-2.72202900	0.84526200
C	7.00757300	-0.17649800	0.12607200
H	5.45409800	1.22200200	-0.31099700
C	7.33469800	-1.49354000	0.51987300
H	6.57864600	-3.40476300	1.07524400
O	8.63307900	-1.86374700	0.64468800
H	9.17431900	-1.09284200	0.42534900
C	7.91097800	1.96256600	-0.49429900
H	8.90529200	2.39061700	-0.60726800
H	7.35753000	2.52237600	0.26673200
H	7.37975900	2.01768000	-1.45014400
O	8.10831300	0.61138100	-0.09458700

**Name of radical**	**6-O4’**

Cartesian Coordinates optimized at B3LYP/6-311G(d,p)	Energies at B3LYP/6-311G(d,p) in gas phase
C -3.86726400 -1.80567400 -0.46292100<br>C -5.36720800 -1.69118300 -0.69509500<br>C -6.00280500 -0.82627900 0.38340100<br>C -5.29962700 0.53550000 0.45453200<br>C -3.26155000 -0.40727400 -0.32805700<br>H -5.53525300 -1.21366100 -1.67165300<br>H -3.68463400 -2.33901000 0.47880700<br>H -5.43850300 1.06261300 -0.50076600<br>H -3.33977100 0.13506900 -1.28326600<br>H -5.87788700 -1.33089800 1.35195300	Zero-point correction= 0.418994 (Hartree/Particle)<br>Thermal correction to Energy= 0.447665<br>Thermal correction to Enthalpy= 0.448609<br>Thermal correction to Gibbs Free Energy= 0.356498<br>Sum of electronic and zero-point Energies= -1490.991772<br>Sum of electronic and thermal Energies= -1490.963101<br>Sum of electronic and thermal Enthalpies= -1490.962157<br>Sum of electronic and thermal Free Energies= -1491.054269

O	-3.90367400	0.31660800	0.70385000	
H	-7.67295700	1.18160400	1.02428900	
C	-5.83623800	1.42987000	1.57344200	
H	-5.16794700	2.28590600	1.68413600	
H	-5.82269900	0.86941900	2.52005900	
O	-7.12168200	1.93627600	1.27021300	
O	-7.38669300	-0.61436000	0.11601000	
H	-7.77888100	-1.48185200	-0.04109900	
O	-6.01396800	-2.95722800	-0.65599500	
H	-5.58965100	-3.51518100	-1.31765200	
O	-3.32735100	-2.51655100	-1.56796600	
H	-2.38600000	-2.63895700	-1.40397000	
O	-1.91249700	-0.58805200	0.01275800	
C	-1.01851900	0.44766500	-0.14985600	
C	0.32384900	0.08982600	-0.06997700	
C	-1.40564200	1.76737000	-0.37285100	
C	1.32165200	1.06334100	-0.22373000	
H	0.56371800	-0.94982200	0.10547700	
C	-0.41242500	2.73523300	-0.54238500	
H	-2.44147600	2.07150900	-0.37953300	
C	0.93420100	2.39395000	-0.47310700	
H	1.69402800	3.15945900	-0.59876800	
O	-0.83917300	4.01128300	-0.76412000	
H	-0.07750600	4.59542400	-0.83833500	
C	3.30640200	-0.43288900	0.18333000	
H	2.64608800	-1.26215200	0.41982400	
C	2.74450800	0.76286800	-0.14270700	
H	3.39652900	1.60440500	-0.35760600	
C	4.70570000	-0.74137800	0.26981600	
C	5.08123700	-2.06079700	0.67038500	
C	5.73347600	0.19777700	-0.02138700	
C	6.38765600	-2.43059200	0.78085700	
H	4.29341300	-2.77371600	0.89115900	
C	7.06031100	-0.14500700	0.07995000	
H	5.45815000	1.19648400	-0.33061700	
C	7.47561100	-1.50912600	0.49668600	
H	6.67823600	-3.42890700	1.08570300	
O	8.66427800	-1.82431800	0.58988400	
C	7.83841400	2.00559400	-0.58165300	
H	8.81467300	2.46446600	-0.72272900	

H	7.28361300	2.55376800	0.18732000		
H	7.28198500	2.03104000	-1.52471300		
O	8.09729800	0.66761900	-0.17339200		
**Name of anion**	6-O3				
Cartesian Coordinates optimized at B3LYP/6-311G(d,p				Energies at B3LYP/6-311G(d,p) in gas phase	
C	3.76581300	-1.73391300	0.14574900	Zero-point correction=	0.417964 (Hartree/Particle)
C	5.17898000	-1.76497500	0.69623200	Thermal correction to Energy=	0.446634
C	6.09012100	-0.91094900	-0.16857500	Thermal correction to Enthalpy=	0.447578
C	5.51620200	0.50999900	-0.29502000	Thermal correction to Gibbs Free Energy=	0.354840
C	3.29134800	-0.28424400	0.01155700	Sum of electronic and zero-point Energies=	-1491.074335
H	5.16817300	-1.34946600	1.71422100	Sum of electronic and thermal Energies=	-1491.045665
H	3.75197800	-2.17739900	-0.85930300	Sum of electronic and thermal Enthalpies=	-1491.044721
H	5.49809300	0.97595900	0.70187600	Sum of electronic and thermal Free Energies=	-1491.137459
H	3.25226700	0.20839900	0.99409000		
H	6.13465900	-1.35447700	-1.17316700		
O	4.20155500	0.42531700	-0.83786500		
H	7.98797100	0.90082900	-0.35205100		
C	6.33757300	1.40200500	-1.22675200		
H	5.79605300	2.33794200	-1.37303300		
H	6.42313300	0.90683500	-2.20624500		
O	7.60988900	1.72516200	-0.68556300		
O	7.40684800	-0.85007500	0.39177600		
H	7.65533400	-1.75866100	0.59948200		
O	5.72662700	-3.08598100	0.72655700		
H	5.07808800	-3.63623300	1.18034900		
O	2.94103500	-2.47719200	1.03455900		
H	2.02996800	-2.28958700	0.77322600		
O	2.03688700	-0.33169200	-0.56336700		
C	1.07675300	0.64532800	-0.20026900		
C	-0.23987700	0.19682200	-0.24804900		
C	1.43998100	1.92974100	0.15909700		
C	-1.25394600	1.12064500	0.09370500		
H	-0.44950300	-0.81913600	-0.55513800		
C	0.44328200	2.91376500	0.53791500		
H	2.47281600	2.25484800	0.12848400		
C	-0.91721500	2.41880600	0.47407900		
H	-1.69835700	3.12769500	0.73650600		
O	0.73037800	4.08683400	0.87771700		
C	-3.23274800	-0.42609700	-0.20863300		
H	-2.58158500	-1.26106300	-0.45308900		

C -2.67288300 0.76659200 0.07861000<br>H -3.32742800 1.59428100 0.34607300<br>C -4.66003800 -0.74821000 -0.21741000<br>C -5.07411500 -2.07237900 -0.42463800<br>C -5.66909900 0.22251200 -0.02644200<br>C -6.42239400 -2.42968000 -0.43413700<br>H -4.32249700 -2.83935200 -0.57745500<br>C -7.00667800 -0.13203500 -0.03425000<br>H -5.38685000 1.25571300 0.12021700<br>C -7.40006700 -1.46803400 -0.23809700<br>H -6.73025800 -3.45687200 -0.59323900<br>O -8.72885000 -1.80124700 -0.24555600<br>H -9.21417800 -0.98006300 -0.09261200<br>C -7.78693900 2.10680100 0.35609300<br>H -8.75174300 2.59945700 0.47326100<br>H -7.25705600 2.54293100 -0.49760900<br>H -7.19027200 2.25417600 1.26273600<br>O -8.06976000 0.73240500 0.14147400	
**Name of anion**	6-O4’
Cartesian Coordinates optimized at B3LYP/6-311G(d,p	Energies at B3LYP/6-311G(d,p) in gas phase
C -3.68832200 -1.80056000 -0.19288700<br>C -5.11561300 -1.83175800 -0.71125200<br>C -6.00463200 -0.95566000 0.15522800<br>C -5.42313300 0.46338000 0.23972200<br>C -3.20705600 -0.35188300 -0.08206500<br>H -5.12412000 -1.43561000 -1.73727900<br>H -3.65400600 -2.23527400 0.81503400<br>H -5.41947800 0.90727700 -0.76728900<br>H -3.16449700 0.12100500 -1.07488600<br>H -6.03058000 -1.37905200 1.16902400<br>O -4.09539400 0.38390800 0.75977800<br>H -7.89438600 0.89645300 0.32104600<br>C -6.22394900 1.38194700 1.16380900<br>H -5.66617000 2.31052700 1.29731800<br>H -6.31700400 0.90249700 2.14996300<br>O -7.48763800 1.72148900 0.61732200<br>O -7.32959700 -0.89261300 -0.37914200<br>H -7.59836800 -1.80244700 -0.55413100<br>O -5.66858600 -3.14689600 -0.70025800<br>H -5.04252200 -3.71251500 -1.16688700	Zero-point correction= 0.417796 (Hartree/Particle)<br>Thermal correction to Energy= 0.446335<br>Thermal correction to Enthalpy= 0.447279<br>Thermal correction to Gibbs Free Energy= 0.356238<br>Sum of electronic and zero-point Energies= -1491.077617<br>Sum of electronic and thermal Energies= -1491.049078<br>Sum of electronic and thermal Enthalpies= -1491.048134<br>Sum of electronic and thermal Free Energies= -1491.139176

O -2.89678000 -2.55603400 -1.09897800<br>H -1.98028700 -2.47205000 -0.80911600<br>O -1.94380800 -0.39781400 0.49183300<br>C -1.02998500 0.62422300 0.21053300<br>C 0.30217300 0.25035200 0.22989100<br>C -1.44802900 1.92813200 -0.05526600<br>C 1.32140000 1.19892400 -0.02886200<br>H 0.54021300 -0.78327400 0.44313500<br>C -0.44756100 2.86699600 -0.32530000<br>H -2.48350900 2.22972900 -0.00517400<br>C 0.89508100 2.52035100 -0.31659100<br>H 1.64448400 3.27940400 -0.52909600<br>O -0.86348800 4.15276300 -0.58832100<br>H -0.07920400 4.69364300 -0.72426500<br>C 3.30018100 -0.32386000 0.24995800<br>H 2.62500100 -1.13871200 0.50956600<br>C 2.72960300 0.89184400 -0.03115800<br>H 3.37961100 1.72590000 -0.28225800<br>C 4.67313800 -0.69838500 0.25908000<br>C 5.03788100 -2.02635400 0.59391400<br>C 5.74283900 0.19904100 -0.05697600<br>C 6.34524700 -2.44678000 0.61897300<br>H 4.24344500 -2.72932500 0.83879200<br>C 7.05253600 -0.20142500 -0.03768300<br>H 5.49404300 1.22017600 -0.31903000<br>C 7.45870700 -1.57809600 0.30763200<br>H 6.60269500 -3.46907100 0.87860300<br>O 8.65040500 -1.93626700 0.32283500<br>C 7.84321600 1.95164300 -0.67276200<br>H 8.80792900 2.42455300 -0.86524500<br>H 7.33635500 2.48934900 0.14165900<br>H 7.21984400 2.02857300 -1.57545500<br>O 8.11965800 0.61594100 -0.33277600		
**Name of cationic radical**		Energies at B3LYP/6-311G(d,p) in gas phase
C -3.98665100 -1.83812600 -0.51131500<br>C -5.50215100 -1.66866600 -0.56743600<br>C -5.98009300 -0.76480000 0.56178700<br>C -5.23154800 0.57177800 0.52779100<br>C -3.32633900 -0.45964300 -0.45223800<br>H -5.76728800 -1.20057400 -1.52682000		Zero-point correction= 0.432430 (Hartree/Particle)<br>Thermal correction to Energy= 0.461306<br>Thermal correction to Enthalpy= 0.462250<br>Thermal correction to Gibbs Free Energy= 0.370048<br>Sum of electronic and zero-point Energies= -1491.370397<br>Sum of electronic and thermal Energies= -1491.341521

H	-3.71997100	-2.38051800	0.40409600	Sum of electronic and thermal Enthalpies= -1491.340577
H	-5.45440000	1.08970200	-0.41578300	Sum of electronic and thermal Free Energies= -1491.432779
H	-3.46622500	0.06383600	-1.41129200	
H	-5.76208000	-1.26071700	1.51859200	
O	-3.81647700	0.30509200	0.61947200	
H	-7.50576100	1.37250700	1.33326500	
C	-5.60824400	1.50567200	1.68010100	
H	-4.88079600	2.31911900	1.72145100	
H	-5.54851500	0.95368300	2.62959900	
O	-6.87582300	2.09115900	1.47672700	
O	-7.37084500	-0.49728500	0.44943100	
H	-7.82283200	-1.34688400	0.37587000	
O	-6.17702800	-2.90729600	-0.42184300	
H	-5.92150800	-3.47201300	-1.15991600	
O	-3.60483200	-2.55848700	-1.67308500	
H	-2.69783200	-2.85993100	-1.56137400	
O	-1.93925700	-0.67466900	-0.23508100	
C	-1.06808000	0.36032800	-0.35951000	
C	0.27873300	0.03755000	-0.23504100	
C	-1.45237000	1.68540200	-0.59335800	
C	1.26553000	1.04267800	-0.35809800	
H	0.53435200	-0.99477200	-0.04276700	
C	-0.47318900	2.68846300	-0.72968300	
H	-2.49058200	1.97766100	-0.64216600	
C	0.87219400	2.37723900	-0.61591200	
H	1.62500700	3.15175700	-0.71256900	
O	-0.94474200	3.93143300	-0.96161300	
H	-0.22369300	4.56803400	-1.02616700	
C	3.23917500	-0.45262800	0.05354600	
H	2.58378400	-1.30432600	0.19903700	
C	2.66624800	0.76687300	-0.22939700	
H	3.31727800	1.62481500	-0.36522900	
C	4.62832400	-0.73355100	0.19056900	
C	5.00277100	-2.07280700	0.50933800	
C	5.65405300	0.24077200	0.03021800	
C	6.31842300	-2.43704800	0.66522400	
H	4.22467100	-2.81639200	0.63224400	
C	6.97156800	-0.11451500	0.18340100	
H	5.39954100	1.26180900	-0.21395900	
C	7.31997500	-1.46986800	0.50622200	

H	6.61084000	-3.45032600	0.90831200	
O	8.59093600	-1.80665800	0.65368500	
H	9.14885400	-1.02346900	0.51081200	
C	7.88934300	2.06875800	-0.25044700	
H	8.89311800	2.48350100	-0.28449800	
H	7.30756700	2.56947600	0.52752700	
H	7.40814300	2.18255200	-1.22506700	
O	8.05585700	0.67774800	0.06633500	

**Name of compound (7)**	Rhapontin
Cartesian Coordinates optimized at B3LYP/6-311G(d,p)	Energies at B3LYP/6-311G(d,p) in gas phase

O	-1.19928600	3.44389400	0.86145600	Zero-point correction= 0.431955 (Hartree/Particle)
C	-0.79353900	2.28226200	0.26956600	Thermal correction to Energy= 0.460923
C	0.54316100	1.90195600	0.21371500	Thermal correction to Enthalpy= 0.461868
C	-1.79808400	1.49153800	-0.29859000	Thermal correction to Gibbs Free Energy= 0.369411
C	0.91300900	0.70254500	-0.41877500	Sum of electronic and zero-point Energies= -1491.610790
H	1.29875600	2.55372100	0.63924100	Sum of electronic and thermal Energies= -1491.581821
C	-1.43051400	0.30158200	-0.91715500	Sum of electronic and thermal Enthalpies= -1491.580877
H	-2.82472200	1.82403500	-0.28244000	Sum of electronic and thermal Free Energies= -1491.673333
C	-0.09370600	-0.09292400	-0.98230500	
H	0.15052500	-1.01639200	-1.49360300	
O	-2.32810300	-0.53629400	-1.55463100	
C	3.36354000	0.75168800	0.13590600	
H	3.20348900	1.56559400	0.83846800	
C	2.30159700	0.24477600	-0.51549700	
H	2.44840100	-0.60624000	-1.17487500	
C	4.75577900	0.30985200	0.03281500	
C	5.68780300	0.80557100	0.96351600	
C	5.21346500	-0.57784700	-0.95054400	
C	7.01756100	0.42139000	0.92627700	
H	5.37973300	1.49849200	1.73822200	
C	6.54927500	-0.97055600	-0.99787900	
H	4.53384500	-0.95823800	-1.70253900	
C	7.45489300	-0.47842100	-0.06334900	
O	8.79209900	-0.77527800	0.00625600	
C	-3.48829000	-1.33770700	0.46513700	
C	-4.89020800	-1.65652600	0.96861900	
C	-5.81334800	-0.46468700	0.76478900	
C	-5.79032700	-0.02730500	-0.70551700	
C	-3.57546200	-0.84486400	-0.98812500	

H	-5.28311400	-2.50578900	0.39003800	
H	-3.05789000	-0.54973200	1.09309000	
H	-6.17210100	-0.84800700	-1.33018700	
H	-3.95728900	-1.65454900	-1.62229500	
H	-5.44810800	0.36918600	1.38103600	
O	-4.43966800	0.27886500	-1.07176900	
H	-8.18995600	0.46277100	-0.09606500	
C	-6.64270100	1.21253200	-0.97928300	
H	-6.41970200	1.56833200	-1.98674400	
H	-6.36331100	2.00761600	-0.27156200	
O	-8.02656500	0.92102000	-0.93099500	
O	-7.15321700	-0.79042300	1.12772800	
H	-7.11923800	-1.17747900	2.01089000	
O	-4.90100200	-1.96975100	2.35624100	
H	-4.27922100	-2.69512900	2.48559000	
O	-2.73316000	-2.54003100	0.57414800	
H	-1.80810200	-2.33067600	0.40629000	
H	-0.42881800	3.91870600	1.18984100	
H	6.87617500	-1.65319600	-1.77139600	
O	7.89662600	0.91271500	1.84129200	
H	8.76084000	0.52837000	1.64272800	
C	9.33992200	-1.66973300	-0.95447100	
H	10.39739800	-1.76020400	-0.71259300	
H	8.86644900	-2.65516000	-0.89215700	
H	9.23120600	-1.27447700	-1.96988700	
**Name of radical**	**7-O3**			
Cartesian Coordinates optimized at B3LYP/6-311G(d,p)				Energies at B3LYP/6-311G(d,p) in gas phase
O	-1.08867600	3.25145400	1.02077000	Zero-point correction= 0.418661 (Hartree/Particle)
C	-0.76612900	2.16155000	0.49625200	Thermal correction to Energy= 0.447433
C	0.62446000	1.79015100	0.35042400	Thermal correction to Enthalpy= 0.448377
C	-1.77924700	1.24315200	0.00745500	Thermal correction to Gibbs Free Energy= 0.355563
C	0.99962000	0.58400700	-0.22892300	Sum of electronic and zero-point Energies= -1490.977679
H	1.34856300	2.51449900	0.70086100	Sum of electronic and thermal Energies= -1490.948907
C	-1.39684000	0.05238300	-0.55826200	Sum of electronic and thermal Enthalpies= -1490.947963
H	-2.81014400	1.55304500	0.07212300	Sum of electronic and thermal Free Energies= -1491.040777
C	-0.02571300	-0.27373200	-0.68334700	
H	0.22214800	-1.21915300	-1.15223000	
O	-2.23020500	-0.91308900	-1.08006300	
C	3.46906700	0.70872900	0.18247200	
H	3.31685800	1.54242900	0.86319400	

C	2.39116100	0.15099200	-0.39673700	
H	2.52331500	-0.71834800	-1.03486300	
C	4.86618000	0.30205700	0.02655900	
C	5.82566100	0.86137300	0.89089100	
C	5.30067400	-0.61379900	-0.94130600	
C	7.16218500	0.51002900	0.80586400	
H	5.53446100	1.57853300	1.64974700	
C	6.64303000	-0.97290800	-1.03717100	
H	4.59729000	-1.04364200	-1.64320900	
C	7.57700200	-0.41923600	-0.16671300	
O	8.92215200	-0.67969600	-0.14788200	
C	-3.80475000	-1.31643900	0.76879800	
C	-5.29978200	-1.41067600	1.04876100	
C	-6.02036200	-0.17553000	0.52887900	
C	-5.71047500	0.02456600	-0.95997100	
C	-3.59135000	-1.02162700	-0.72550400	
H	-5.69545100	-2.29215700	0.52280300	
H	-3.38198600	-0.50814700	1.37568900	
H	-6.07977200	-0.84331500	-1.52495700	
H	-3.93576500	-1.88576900	-1.30733500	
H	-5.65560600	0.70231000	1.08112900	
O	-4.28927000	0.14188500	-1.11940800	
H	-8.10532300	0.83386000	-0.86787400	
C	-6.35249400	1.28155300	-1.54875000	
H	-5.92295900	1.45723200	-2.53659000	
H	-6.10595500	2.14683700	-0.91559200	
O	-7.74825500	1.12538400	-1.71709200	
O	-7.42997000	-0.30427400	0.69050500	
H	-7.58702500	-0.54997800	1.61031200	
O	-5.57740200	-1.50944400	2.43961600	
H	-5.10448000	-2.28113600	2.77122900	
O	-3.24494200	-2.57233000	1.13816700	
H	-2.28747100	-2.51470100	1.05608200	
H	6.95286100	-1.67817800	-1.79725300	
O	8.06825400	1.06053900	1.65721100	
H	8.93311300	0.69063500	1.43538500	
C	9.45081300	-1.59907300	-1.09645100	
H	10.51945600	-1.65480700	-0.89738500	
H	9.00524800	-2.59178800	-0.97473800	
H	9.28886500	-1.24683200	-2.12043900	

Name of radical	7-O3’
Cartesian Coordinates optimized at B3LYP/6-311G(d,p)	Energies at B3LYP/6-311G(d,p) in gas phase

O	-1.18217000	3.45019200	0.82863800	Zero-point correction=	0.418970 (Hartree/Particle)
C	-0.76041800	2.28044800	0.26753400	Thermal correction to Energy=	0.447744
C	0.57962600	1.90995300	0.23134700	Thermal correction to Enthalpy=	0.448688
C	-1.75391400	1.46685400	-0.28881100	Thermal correction to Gibbs Free Energy=	0.356108
C	0.96142300	0.69930400	-0.37152500	Sum of electronic and zero-point Energies=	-1490.979951
H	1.32572500	2.57503700	0.65251500	Sum of electronic and thermal Energies=	-1490.951178
C	-1.37448100	0.26491300	-0.87578500	Sum of electronic and thermal Enthalpies=	-1490.950234
H	-2.78299300	1.79193600	-0.28687900	Sum of electronic and thermal Free Energies=	-1491.042813
C	-0.03308200	-0.11867300	-0.92247000		
H	0.22242500	-1.05243800	-1.40882100		
O	-2.25712700	-0.59721900	-1.49803200		
C	3.41599900	0.80271300	0.16159200		
H	3.26230000	1.66846200	0.79950800		
C	2.35343000	0.24791600	-0.44855600		
H	2.49893000	-0.63781100	-1.06040400		
C	4.80831400	0.35897400	0.08740300		
C	5.76549000	0.99396900	0.85320300		
C	5.23080400	-0.71251300	-0.75435700		
C	7.16164600	0.62234800	0.85007000		
H	5.50422300	1.81722500	1.50885500		
C	6.55627900	-1.12774000	-0.81250600		
H	4.50745800	-1.22155300	-1.37882500		
C	7.52866200	-0.49766300	-0.03855800		
O	8.82541600	-0.81469800	-0.01693400		
C	-3.47260300	-1.34024800	0.51125300		
C	-4.88876500	-1.63236900	0.99095500		
C	-5.79475300	-0.43703200	0.73662300		
C	-5.73509800	-0.03737800	-0.74324400		
C	-3.52228200	-0.88069200	-0.95465300		
H	-5.27725300	-2.49205000	0.42504700		
H	-3.04701800	-0.54304100	1.13099700		
H	-6.11010200	-0.87003900	-1.35585100		
H	-3.89430200	-1.70282300	-1.57838000		
H	-5.43432400	0.40851200	1.33973000		
O	-4.37296000	0.24726200	-1.08669800		
H	-8.14557800	0.48969300	-0.20429500		
C	-6.56975600	1.20235600	-1.06769700		
H	-6.31888300	1.53190600	-2.07753800		

H	-6.30164900	2.01230600	-0.37256400	
O	-7.95633200	0.92228200	-1.04740000	
O	-7.14537900	-0.73992400	1.07711100	
H	-7.13593500	-1.10369400	1.97073100	
O	-4.93328800	-1.90968600	2.38521200	
H	-4.32835200	-2.64278800	2.54653800	
O	-2.73429900	-2.54825000	0.66373300	
H	-1.80414300	-2.35533900	0.50584700	
H	-0.42079300	3.94203300	1.15337000	
H	6.81936400	-1.94492300	-1.47150900	
O	8.01175400	1.20047300	1.54706100	
C	9.29566400	-1.88319400	-0.83701400	
H	10.36399000	-1.95012300	-0.64474400	
H	8.81337500	-2.82760100	-0.56696500	
H	9.12550600	-1.66981400	-1.89671400	
**Name of anion**	7-O3			
Cartesian Coordinates optimized at B3LYP/6-311G(d,p				Energies at B3LYP/6-311G(d,p) in gas phase
O	-1.13856700	3.86902600	1.03903300	Zero-point correction= 0.418275 (Hartree/Particle)
C	-0.87683100	2.83088800	0.39011100	Thermal correction to Energy= 0.446663
C	0.44526900	2.23675100	0.34617200	Thermal correction to Enthalpy= 0.447608
C	-1.87919100	2.11278600	-0.38519600	Thermal correction to Gibbs Free Energy= 0.355964
C	0.74736600	1.09096600	-0.37929500	Sum of electronic and zero-point Energies= -1491.072138
H	1.20844700	2.75641300	0.91624800	Sum of electronic and thermal Energies= -1491.043750
C	-1.56109400	0.97292800	-1.08793400	Sum of electronic and thermal Enthalpies= -1491.042805
H	-2.88957100	2.50304700	-0.40376400	Sum of electronic and thermal Free Energies= -1491.134449
C	-0.27423100	0.41994700	-1.10234500	
H	-0.04751300	-0.40365300	-1.77054400	
O	-2.53428600	0.29368700	-1.85943800	
C	3.21819900	0.96025100	0.14164300	
H	3.15813800	1.85334400	0.75707400	
C	2.09259000	0.51213100	-0.44760100	
H	2.15407200	-0.39078500	-1.05338400	
C	4.55227900	0.36322200	0.05282100	
C	5.59649200	0.92125100	0.81712500	
C	4.86514600	-0.74083200	-0.75777600	
C	6.88095000	0.40398900	0.78125600	
H	5.40797700	1.77577300	1.45714600	
C	6.15398100	-1.26892100	-0.80036800	
H	4.09900200	-1.19553200	-1.37269900	
C	7.16661500	-0.70274300	-0.03349100	

O	8.48528500	-1.12007900	0.02000600	
C	-3.08371700	-1.03921600	0.14618200	
C	-4.24620800	-1.82241900	0.73376000	
C	-5.46529900	-0.92863700	0.86524400	
C	-5.79760500	-0.30645600	-0.49820400	
C	-3.51437300	-0.43241500	-1.20041500	
H	-4.49206800	-2.65430100	0.05510500	
H	-2.82307500	-0.21815600	0.82198500	
H	-6.06644300	-1.11638500	-1.19611200	
H	-3.80751000	-1.23599700	-1.89255300	
H	-5.23250100	-0.11952800	1.57041300	
O	-4.66285200	0.40671400	-0.97349900	
H	-8.06310000	-0.59495200	0.52603400	
C	-6.97006500	0.67275800	-0.43841800	
H	-7.04280400	1.18341500	-1.40049400	
H	-6.76460300	1.43293600	0.32939600	
O	-8.20843900	0.01311100	-0.21139400	
O	-6.59635800	-1.67370300	1.33805600	
H	-6.29501200	-2.15406600	2.11825100	
O	-3.93433100	-2.34315600	2.02452400	
H	-3.03257600	-2.68133900	1.95234300	
O	-1.99616000	-1.94942100	0.02386700	
H	-1.20547500	-1.40386500	-0.13779500	
H	6.35803600	-2.11999200	-1.43832600	
O	7.87173700	0.96846600	1.53716300	
H	8.67421000	0.46018800	1.36285400	
C	8.86254800	-2.22971000	-0.77402000	
H	9.92348500	-2.39338000	-0.58599300	
H	8.30263500	-3.13041500	-0.49599600	
H	8.71107800	-2.02903300	-1.84124000	
**Name of anion**	7-O3’			
Cartesian Coordinates optimized at B3LYP/6-311G(d,p				Energies at B3LYP/6-311G(d,p) in gas phase
O	-1.12991700	3.73328800	0.93625800	Zero-point correction= 0.417450 (Hartree/Particle)
C	-0.80172400	2.60190000	0.23402700	Thermal correction to Energy= 0.445965
C	0.49741200	2.11147100	0.20141500	Thermal correction to Enthalpy= 0.446909
C	-1.84311400	1.96463600	-0.45067000	Thermal correction to Gibbs Free Energy= 0.355902
C	0.82335600	0.95200100	-0.53417500	Sum of electronic and zero-point Energies= -1491.060223
H	1.27326900	2.64352800	0.74223400	Sum of electronic and thermal Energies= -1491.031707
C	-1.52661000	0.81867700	-1.17045000	Sum of electronic and thermal Enthalpies= -1491.030763
H	-2.84912900	2.35697200	-0.42952800	Sum of electronic and thermal Free Energies= -1491.121770

C	-0.23145700	0.31352000	-1.22291200	
H	-0.03280700	-0.55312800	-1.84412500	
O	-2.49856200	0.14037200	-1.90918600	
C	3.25957900	0.83671000	0.05190800	
H	3.14188300	1.70021200	0.70490900	
C	2.16196900	0.39049000	-0.60775000	
H	2.25725400	-0.47921700	-1.25171200	
C	4.61189100	0.31340600	0.02806700	
C	5.57389200	0.94168500	0.84119900	
C	4.98980900	-0.78706400	-0.76352500	
C	6.94865000	0.54633200	0.94134700	
H	5.28541400	1.79267700	1.45376100	
C	6.31788400	-1.22555500	-0.71620900	
H	4.28015700	-1.29234400	-1.40746300	
C	7.26391200	-0.60376100	0.09038700	
O	8.58067600	-1.00203200	0.16778800	
C	-3.09834300	-1.22028700	0.06088800	
C	-4.30979600	-1.89658800	0.68547100	
C	-5.41905100	-0.88333800	0.91055100	
C	-5.74829200	-0.17084600	-0.40805000	
C	-3.53218600	-0.50537600	-1.23007200	
H	-4.67680900	-2.67067300	-0.00582700	
H	-2.70891600	-0.47425700	0.76161700	
H	-6.13166400	-0.91300800	-1.12513800	
H	-3.92500300	-1.23860200	-1.94675400	
H	-5.06579700	-0.13365200	1.63207100	
O	-4.56107100	0.43892500	-0.91398900	
H	-7.99773500	-0.25761000	0.70440500	
C	-6.80303200	0.92452200	-0.24944500	
H	-6.85423600	1.49226000	-1.18037900	
H	-6.48775900	1.61401500	0.54791100	
O	-8.09390100	0.38941900	-0.00727100	
O	-6.59900900	-1.52000600	1.40804000	
H	-6.32221200	-2.06860100	2.15179300	
O	-3.99960500	-2.48591500	1.94357500	
H	-3.19297500	-2.99846500	1.80924100	
O	-2.13542900	-2.24093200	-0.17455300	
H	-1.28090300	-1.80927200	-0.30471400	
H	-0.32985400	4.07728000	1.34687900	
H	6.60237900	-2.07332400	-1.33104400	

O	7.80183300	1.10768800	1.66855800	
C	8.98857400	-2.09482500	-0.61344300	
H	10.05073800	-2.24050100	-0.40629500	
H	8.44643600	-3.01732100	-0.35676100	
H	8.85821600	-1.90967600	-1.69020100	
**Name of cationic radical**				Energies at B3LYP/6-311G(d,p) in gas phase
C	-3.98669100	-1.83806000	-0.51175700	Zero-point correction= 0.432431 (Hartree/Particle)
C	-5.50220500	-1.66857400	-0.56743800	Thermal correction to Energy= 0.461307
C	-5.97992100	-0.76480100	0.56196200	Thermal correction to Enthalpy= 0.462251
C	-5.23128800	0.57174600	0.52808300	Thermal correction to Gibbs Free Energy= 0.370041
C	-3.32634200	-0.45961400	-0.45255900	Sum of electronic and zero-point Energies= -1491.370395
H	-5.76761200	-1.20041700	-1.52671500	Sum of electronic and thermal Energies= -1491.341520
H	-3.71978000	-2.38064100	0.40347500	Sum of electronic and thermal Enthalpies= -1491.340576
H	-5.45425200	1.08991800	-0.41532500	Sum of electronic and thermal Free Energies= -1491.432785
H	-3.46642300	0.06405600	-1.41148000	
H	-5.76176600	-1.26084600	1.51867200	
O	-3.81622000	0.30490600	0.61941500	
H	-7.50525100	1.37251500	1.33447700	
C	-5.60758700	1.50546900	1.68065400	
H	-4.88007300	2.31886900	1.72188400	
H	-5.54761100	0.95337500	2.63006000	
O	-6.87518300	2.09107700	1.47773800	
O	-7.37068800	-0.49728000	0.44990400	
H	-7.82263100	-1.34690300	0.37632600	
O	-6.17702800	-2.90723000	-0.42176200	
H	-5.92180400	-3.47184400	-1.16001400	
O	-3.60519800	-2.55817600	-1.67378600	
H	-2.69826400	-2.85989300	-1.56228000	
O	-1.93921300	-0.67471100	-0.23574000	
C	-1.06806400	0.36032400	-0.35992000	
C	0.27875100	0.03757400	-0.23537600	
C	-1.45236300	1.68541400	-0.59368500	
C	1.26553100	1.04273000	-0.35830500	
H	0.53438200	-0.99476200	-0.04319500	
C	-0.47319600	2.68850700	-0.72985800	
H	-2.49057400	1.97766200	-0.64258200	
C	0.87218800	2.37730100	-0.61605800	
H	1.62498500	3.15184500	-0.71261000	
O	-0.94476000	3.93148900	-0.96170000	
H	-0.22372200	4.56811900	-1.02610100	

C	3.23912500	-0.45256400	0.05355200	
H	2.58370400	-1.30424200	0.19902700	
C	2.66624400	0.76693100	-0.22951000	
H	3.31729900	1.62484600	-0.36539800	
C	4.62824900	-0.73350800	0.19066800	
C	5.00267600	-2.07284400	0.50914800	
C	5.65400400	0.24085900	0.03078500	
C	6.31830300	-2.43713700	0.66515000	
H	4.22456200	-2.81646400	0.63174800	
C	6.97148600	-0.11451900	0.18401300	
H	5.39961900	1.26203900	-0.21297100	
C	7.31988600	-1.46991700	0.50654900	
H	6.61065600	-3.45048400	0.90802600	
O	8.59085600	-1.80668000	0.65406900	
H	9.14875700	-1.02342700	0.51146800	
C	7.88858000	2.06835700	-0.25172200	
H	8.89223800	2.48321300	-0.28767700	
H	7.30761000	2.57043700	0.52597600	
H	7.40615900	2.18018500	-1.22598700	
O	8.05563200	0.67795600	0.06747800	

**Name of compound (8)**	*trans*-Pterostilbene			
Cartesian Coordinates optimized at B3LYP/6-311G(d,p)				Energies at B3LYP/6-311G(d,p) in gas phase
C	3.81296400	0.07716100	0.00179200	Zero-point correction= 0.282380 (Hartree/Particle)
C	3.25210200	-1.20367500	0.01219700	Thermal correction to Energy= 0.300268
C	1.86835900	-1.37393800	0.00578400	Thermal correction to Enthalpy= 0.301212
C	1.01220900	-0.26518400	-0.00995600	Thermal correction to Gibbs Free Energy= 0.233408
C	1.57187800	1.02073600	-0.02205000	Sum of electronic and zero-point Energies= -844.908747
C	2.95277400	1.18465300	-0.01560900	Sum of electronic and thermal Energies= -844.890858
H	4.88297700	0.21154800	0.00586700	Sum of electronic and thermal Enthalpies= -844.889914
H	1.47459100	-2.38282100	0.01479700	Sum of electronic and thermal Free Energies= -844.957719
H	0.95974300	1.91228800	-0.03940500	
C	-1.40755500	0.41953400	0.00717300	
H	-1.12295700	1.46827000	0.03412000	
C	-2.85262800	0.19150300	0.00256900	
C	-3.71746000	1.29482000	0.06525800	
C	-3.44500400	-1.08481000	-0.06328800	
C	-5.09978000	1.14439000	0.06592700	
H	-3.29672300	2.29367600	0.11566400	
C	-4.81921000	-1.24862000	-0.06346600	

H -2.81989800 -1.96802300 -0.11822800<br>C -5.65902300 -0.13150700 0.00186500<br>H -5.74242000 2.01886000 0.11602400<br>H -5.26697400 -2.23371700 -0.11521000<br>H -0.70906400 -1.55925800 -0.03038400<br>C -0.43409200 -0.50813200 -0.01341300<br>O 3.98748200 -2.35414200 0.02893000<br>O 3.39210000 2.47864500 -0.02862100<br>O -7.00573800 -0.35470500 -0.00188000<br>H -7.46711000 0.48898000 0.04627900<br>C 4.78824000 2.72936100 -0.02525400<br>H 5.27674800 2.31065900 -0.91270700<br>H 4.89518700 3.81300100 -0.03741100<br>H 5.26868300 2.33151200 0.87608300<br>C 5.40293600 -2.26035200 0.03901800<br>H 5.78093000 -1.75635200 -0.85797300<br>H 5.76666100 -1.73670700 0.93063700<br>H 5.76862100 -3.28593500 0.05313000	
**Name of radical**	**8-O4’**
Cartesian Coordinates optimized at B3LYP/6-311G(d,p)	Energies at B3LYP/6-311G(d,p) in gas phase
C 3.73886600 0.07721500 0.00006700<br>C 3.18244000 -1.20676900 0.00007100<br>C 1.79944100 -1.37813800 0.00008300<br>C 0.94324900 -0.26597800 0.00007300<br>C 1.50019100 1.02355000 0.00005100<br>C 2.88096000 1.18761500 0.00005200<br>H 4.80890000 0.21227000 0.00012600<br>H 1.40483500 -2.38648900 0.00008900<br>H 0.88749800 1.91450800 0.00003900<br>C -1.47844700 0.43171600 -0.00002700<br>H -1.18882500 1.47864200 -0.00011700<br>C -2.89080100 0.19774600 -0.00002300<br>C -3.77324900 1.32168900 -0.00004900<br>C -3.47755600 -1.10864000 0.00000300<br>C -5.12896900 1.17474100 -0.00004800<br>H -3.33548300 2.31525900 -0.00006900<br>C -4.82761800 -1.28074700 0.00000600<br>H -2.83214400 -1.97928200 0.00001900<br>C -5.74830000 -0.14585500 -0.00002100	Zero-point correction= 0.269635 (Hartree/Particle)<br>Thermal correction to Energy= 0.287137<br>Thermal correction to Enthalpy= 0.288082<br>Thermal correction to Gibbs Free Energy= 0.221819<br>Sum of electronic and zero-point Energies= -844.285757<br>Sum of electronic and thermal Energies= -844.268254<br>Sum of electronic and thermal Enthalpies= -844.267310<br>Sum of electronic and thermal Free Energies= -844.333573

H -5.79759200 2.02768800 -0.00006700<br>H -5.27632300 -2.26743100 0.00002600<br>H -0.77192300 -1.55814800 0.00015900<br>C -0.49220800 -0.50921200 0.00007300<br>O 3.91864000 -2.35301200 0.00009400<br>O 3.32019600 2.47808400 0.00004800<br>O -6.97935000 -0.29852700 -0.00002200<br>C 4.71773300 2.73155000 -0.00006900<br>H 5.20117500 2.32452300 -0.89524400<br>H 4.82130800 3.81521500 0.00018800<br>H 5.20141800 2.32407700 0.89477600<br>C 5.33597100 -2.25959400 -0.00023300<br>H 5.70534700 -1.74675700 -0.89537200<br>H 5.70575200 -1.74627400 0.89446500<br>H 5.69995700 -3.28550600 -0.00002600	
**Name of anion**	8-O4’
Cartesian Coordinates optimized at B3LYP/6-311G(d,p	Energies at B3LYP/6-311G(d,p) in gas phase
C 3.77264300 0.10064200 0.00000400<br>C 3.22579100 -1.19000000 -0.00005400<br>C 1.85397900 -1.39486400 -0.00003500<br>C 0.94695900 -0.31073100 0.00003800<br>C 1.50238700 0.98997900 0.00009800<br>C 2.87457800 1.18013500 0.00008600<br>H 4.83883800 0.25911800 -0.00001700<br>H 1.48722300 -2.41463500 -0.00008400<br>H 0.87411700 1.87079000 0.00017000<br>C -1.47908900 0.34736000 -0.00006000<br>H -1.18109000 1.39585400 -0.00019500<br>C -2.89489900 0.15493100 -0.00003500<br>C -3.76197900 1.28353400 -0.00027400<br>C -3.53262700 -1.11950100 0.00022800<br>C -5.12903600 1.17267200 -0.00026200<br>H -3.30695700 2.27360700 -0.00047400<br>C -4.89540000 -1.25464500 0.00024800<br>H -2.91542800 -2.01461800 0.00043000<br>C -5.80438000 -0.11449600 0.00000400<br>H -5.76394700 2.05435200 -0.00044900<br>H -5.35933500 -2.23736400 0.00045600<br>H -0.73814900 -1.63510500 0.00014800<br>C -0.47428200 -0.58089700 0.00005100	Zero-point correction= 0.268332 (Hartree/Particle)<br>Thermal correction to Energy= 0.285824<br>Thermal correction to Enthalpy= 0.286768<br>Thermal correction to Gibbs Free Energy= 0.221265<br>Sum of electronic and zero-point Energies= -844.368730<br>Sum of electronic and thermal Energies= -844.351237<br>Sum of electronic and thermal Enthalpies= -844.350293<br>Sum of electronic and thermal Free Energies= -844.415797

O 3.99865800 -2.33319400 -0.00014400<br>O 3.29032100 2.49621000 0.00014100<br>O -7.04906900 -0.23896500 0.00002400<br>C 4.67520400 2.76571100 0.00008400<br>H 5.17242600 2.36423200 -0.89261000<br>H 4.77101800 3.85210900 -0.00002400<br>H 5.17247000 2.36439500 0.89283000<br>C 5.40277100 -2.19402600 -0.00012000<br>H 5.76355000 -1.66636300 -0.89266500<br>H 5.76353100 -1.66648900 0.89250800<br>H 5.80672600 -3.20711300 -0.00019000	
**Name of cationic radical**	Energies at B3LYP/6-311G(d,p) in gas phase
C 3.76531500 0.08016100 0.00002300<br>C 3.22466900 -1.22253500 0.00001800<br>C 1.84572100 -1.39897400 -0.00001600<br>C 0.98773800 -0.27904300 -0.00003100<br>C 1.53564600 1.02822200 -0.00003100<br>C 2.91387800 1.20191800 0.00002600<br>H 4.83575800 0.21797100 -0.00003200<br>H 1.45056500 -2.40640400 -0.00001700<br>H 0.91746000 1.91458200 -0.00006800<br>C -1.41792200 0.43500700 0.00002500<br>H -1.12298500 1.47902400 0.00012700<br>C -2.81914900 0.19751000 0.00000700<br>C -3.69771300 1.31898700 0.00015300<br>C -3.40635500 -1.10363600 -0.00015000<br>C -5.06221400 1.16441600 0.00015300<br>H -3.27444300 2.31675600 0.00027000<br>C -4.76509800 -1.26831500 -0.00015500<br>H -2.77672700 -1.98369200 -0.00027500<br>C -5.61225700 -0.13493700 -0.00000100<br>H -5.71598100 2.02998800 0.00026900<br>H -5.22065100 -2.25025800 -0.00027700<br>H -0.70954100 -1.56843600 -0.00010500<br>C -0.42205400 -0.52224400 -0.00005100<br>O 3.96756600 -2.34316000 0.00005500<br>O 3.34918900 2.47725000 0.00012800<br>O -6.92231500 -0.37033800 -0.00001400<br>H -7.43432300 0.44966000 0.00009800<br>C 4.75364200 2.75013800 -0.00022500	Zero-point correction= 0.282889 (Hartree/Particle)<br>Thermal correction to Energy= 0.300643<br>Thermal correction to Enthalpy= 0.301587<br>Thermal correction to Gibbs Free Energy= 0.235293<br>Sum of electronic and zero-point Energies= -844.661960<br>Sum of electronic and thermal Energies= -844.644207<br>Sum of electronic and thermal Enthalpies= -844.643262<br>Sum of electronic and thermal Free Energies= -844.709557

H	5.23361800	2.35047000	-0.89846100	
H	4.83707400	3.83404600	0.00036400	
H	5.23432600	2.34935300	0.89715600	
C	5.39647800	-2.25654800	0.00012200	
H	5.76072400	-1.74884700	-0.89765500	
H	5.76064900	-1.74950300	0.89829600	
H	5.74702000	-3.28548900	-0.00024900	

**Name of compound (9)**	Rhapontigenin
Cartesian Coordinates optimized at B3LYP/6-311G(d,p)	Energies at B3LYP/6-311G(d,p) in gas phase

C	-5.24484700	0.13272000	0.00027300	Zero-point correction= 0.281723 (Hartree/Particle)
C	-4.71135900	-1.15476900	0.00786500	Thermal correction to Energy= 0.299933
C	-3.33230200	-1.35000700	0.00721600	Thermal correction to Enthalpy= 0.300877
C	-2.45493200	-0.25129800	-0.00091400	Thermal correction to Gibbs Free Energy= 0.232659
C	-2.99377200	1.04217300	-0.00889800	Sum of electronic and zero-point Energies= -844.928728
C	-4.37396600	1.22320900	-0.00808800	Sum of electronic and thermal Energies= -844.910518
H	-6.32169100	0.26108000	0.00076000	Sum of electronic and thermal Enthalpies= -844.909574
H	-2.93013700	-2.35890900	0.01334200	Sum of electronic and thermal Free Energies= -844.977792
H	-2.36558300	1.92254000	-0.01654000	
C	-0.02372100	0.39186900	-0.00018100	
H	-0.29075000	1.44549000	0.00188000	
C	1.41726600	0.13965000	-0.00089600	
C	2.29942100	1.23695200	0.00856900	
C	1.98859300	-1.13930100	-0.01096600	
C	3.68102100	1.09990500	0.00886000	
H	1.88194300	2.23953900	0.01619500	
C	3.36775500	-1.31274700	-0.01095100	
H	1.35739700	-2.01990500	-0.01970200	
C	4.21643800	-0.20228600	-0.00105600	
H	3.77242200	-2.31601000	-0.01908200	
H	-0.75458100	-1.57439900	-0.00063600	
C	-1.01377800	-0.51887100	-0.00090300	
O	-5.60020300	-2.19319200	0.01573700	
O	-4.83248200	2.51064700	-0.01615900	
O	5.57928200	-0.27623200	-0.00045600	
H	-5.79496800	2.50347800	-0.01508000	
H	-5.11221800	-3.02291100	0.02075800	
C	4.60274100	2.29101500	0.01925600	
H	5.25607600	2.27707500	0.89647900	
H	5.25797000	2.29089200	-0.85665800	

H 4.03399000 3.22235200 0.02600600	
C 6.19464100 -1.55630200 -0.00986900	
H 5.92645800 -2.13766600 0.87928800	
H 5.92827000 -2.12368200 -0.90855400	
H 7.26742100 -1.37021000 -0.00733600	
**Name of radical**	**9-O3**
Cartesian Coordinates optimized at B3LYP/6-311G(d,p)	Energies at B3LYP/6-311G(d,p) in gas phase
C -5.32288300 0.17563200 -0.00806300	Zero-point correction= 0.268326 (Hartree/Particle)
C -4.78986500 -1.08797500 -0.10784100	Thermal correction to Energy= 0.286304
C -3.39079600 -1.27276000 -0.08923600	Thermal correction to Enthalpy= 0.287249
C -2.48563500 -0.18977900 0.02828100	Thermal correction to Gibbs Free Energy= 0.218771
C -3.01853300 1.08681300 0.13847300	Sum of electronic and zero-point Energies= -844.295262
C -4.44818400 1.32022900 0.12017900	Sum of electronic and thermal Energies= -844.277284
H -6.39258800 0.33758800 -0.02035500	Sum of electronic and thermal Enthalpies= -844.276340
H -2.99265600 -2.28093000 -0.17140600	Sum of electronic and thermal Free Energies= -844.344818
H -2.39825700 1.96659300 0.25081700	
C -0.05056400 0.41385500 0.00363500	
H -0.31049300 1.46850700 -0.04094600	
C 1.38698100 0.15068200 0.01285100	
C 2.27590100 1.23547800 -0.11296400	
C 1.94739200 -1.12644500 0.14346200	
C 3.65578400 1.08699000 -0.11875400	
H 1.86515100 2.23589400 -0.21192900	
C 3.32458900 -1.31108300 0.14171300	
H 1.30987000 -1.99535400 0.25577500	
C 4.18109000 -0.21370100 0.01053800	
H 3.72272700 -2.31139400 0.24660500	
H -0.80501100 -1.54564900 0.04701500	
C -1.04955100 -0.48692500 0.03198100	
O -5.64196100 -2.14764200 -0.22411600	
O -4.90675500 2.48278300 0.21643500	
O 5.54111600 -0.29929100 -0.00031200	
H -5.13677200 -2.96448800 -0.28878000	
C 4.58645300 2.26287300 -0.25632200	
H 5.25569200 2.33902000 0.60541800	
H 5.22489300 2.15962300 -1.13843200	
H 4.02525500 3.19471000 -0.34252300	
C 6.14976700 -1.57733100 0.12779600	
H 5.89174500 -2.05038100 1.08151200	
H 5.86640100 -2.24070300 -0.69659700	

H 7.22315600 -1.39913700 0.09340100		
**Name of radical**	**9-O5**	
Cartesian Coordinates optimized at B3LYP/6-311G(d,p)		Energies at B3LYP/6-311G(d,p) in gas phase
C -5.30834800 0.11493700 0.00000000<br>C -4.80221200 -1.24301000 -0.00000200<br>C -3.36240400 -1.41780100 -0.00000100<br>C -2.48751800 -0.34216200 -0.00000100<br>C -3.03939200 0.96322900 -0.00000100<br>C -4.43498500 1.17633700 0.00000100<br>H -6.38507200 0.24600500 0.00000100<br>H -3.00116600 -2.43958100 -0.00000100<br>H -2.40714300 1.84157800 -0.00000600<br>C -0.06865000 0.34094100 0.00000300<br>H -0.34992100 1.39107400 0.00000700<br>C 1.37542700 0.11287500 0.00000300<br>C 2.23901800 1.22489000 0.00000000<br>C 1.96543600 -1.15764100 0.00000600<br>C 3.62220700 1.10984900 -0.00000100<br>H 1.80584300 2.22083800 -0.00000200<br>C 3.34657700 -1.30898000 0.00000500<br>H 1.34832000 -2.04807100 0.00001000<br>C 4.17766300 -0.18461400 0.00000100<br>H 3.76709700 -2.30559500 0.00000700<br>H -0.77291100 -1.63878200 -0.00000400<br>C -1.04339100 -0.58692100 0.00000000<br>O -5.57381800 -2.22609900 -0.00000300<br>O -4.82768900 2.48263700 0.00000200<br>O 5.53951500 -0.23719200 0.00000000<br>H -5.79002100 2.52566300 0.00000300<br>C 4.52566300 2.31468200 -0.00000400<br>H 5.17987000 2.31719800 0.87657900<br>H 5.17986900 2.31719500 -0.87658700<br>H 3.94312000 3.23744700 -0.00000500<br>C 6.17708800 -1.50762100 -0.00000400<br>H 5.91937700 -2.08556200 0.89415800<br>H 5.91937100 -2.08556100 -0.89416500<br>H 7.24623700 -1.30254400 -0.00000700		Zero-point correction= 0.268627 (Hartree/Particle)<br>Thermal correction to Energy= 0.286516<br>Thermal correction to Enthalpy= 0.287461<br>Thermal correction to Gibbs Free Energy= 0.220063<br>Sum of electronic and zero-point Energies= -844.297998<br>Sum of electronic and thermal Energies= -844.280109<br>Sum of electronic and thermal Enthalpies= -844.279165<br>Sum of electronic and thermal Free Energies= -844.346563
**Name of anion**	9-O3	
Cartesian Coordinates optimized at B3LYP/6-311G(d,p		Energies at B3LYP/6-311G(d,p) in gas phase
C -5.29216200 0.14381600 -0.00003400		Zero-point correction= 0.267153 (Hartree/Particle)

C	-4.73445500	-1.11984900	0.00077400	Thermal correction to Energy= 0.285294
C	-3.34990700	-1.31671500	0.00079400	Thermal correction to Enthalpy= 0.286238
C	-2.50516100	-0.17986600	-0.00002800	Thermal correction to Gibbs Free Energy= 0.218784
C	-3.05578900	1.09920800	-0.00087000	Sum of electronic and zero-point Energies= -844.374528
C	-4.48233500	1.34813900	-0.00091600	Sum of electronic and thermal Energies= -844.356387
H	-6.37156000	0.25545900	-0.00003800	Sum of electronic and thermal Enthalpies= -844.355443
H	-2.92786200	-2.31925300	0.00145000	Sum of electronic and thermal Free Energies= -844.422898
H	-2.42394400	1.98172500	-0.00158800	
C	-0.05933800	0.46838600	-0.00017700	
H	-0.31635300	1.52397200	-0.00028000	
C	1.37501800	0.18542000	-0.00017700	
C	2.28968900	1.25920000	0.00080100	
C	1.93053400	-1.10394100	-0.00117500	
C	3.66968000	1.09158600	0.00088600	
H	1.89392500	2.27090100	0.00154900	
C	3.30813400	-1.30592900	-0.00110100	
H	1.28120400	-1.97138400	-0.00212900	
C	4.18057900	-0.21713800	-0.00007900	
H	3.68901300	-2.31950500	-0.00191000	
H	-0.80105200	-1.48987100	0.00042700	
C	-1.06440400	-0.43226700	0.00005100	
O	-5.59799900	-2.21344000	0.00156300	
O	-4.97720000	2.50340300	-0.00166800	
O	5.55622200	-0.32095600	0.00002200	
H	-5.04669700	-3.00154900	0.00213700	
C	4.61272700	2.26790200	0.00197700	
H	5.26752800	2.25212200	0.87906700	
H	5.26788900	2.25345700	-0.87486400	
H	4.05751100	3.20792200	0.00258400	
C	6.12612300	-1.61400200	-0.00103200	
H	5.84090600	-2.18646700	0.89067600	
H	5.84104400	-2.18494700	-0.89375700	
H	7.20666700	-1.46833100	-0.00082100	
**Name of anion**	9-O5			
Cartesian Coordinates optimized at B3LYP/6-311G(d,p				Energies at B3LYP/6-311G(d,p) in gas phase
C	-5.28127800	0.04168300	0.00003500	Zero-point correction= 0.267542 (Hartree/Particle)
C	-4.77386900	-1.31174400	0.00026400	Thermal correction to Energy= 0.285502
C	-3.32606400	-1.37794700	0.00021400	Thermal correction to Enthalpy= 0.286447
C	-2.49986700	-0.25272400	-0.00002800	Thermal correction to Gibbs Free Energy= 0.219554
C	-3.05823300	1.04655700	-0.00025800	Sum of electronic and zero-point Energies= -844.377049

C	-4.44679300	1.15159900	-0.00021700	Sum of electronic and thermal Energies= -844.359088
H	-6.36410400	0.16488500	0.00005800	Sum of electronic and thermal Enthalpies= -844.358144
H	-2.88950300	-2.37359600	0.00038600	Sum of electronic and thermal Free Energies= -844.425037
H	-2.45495400	1.94468500	-0.00049900	
C	-0.06576500	0.43504600	-0.00001200	
H	-0.33455100	1.48803600	0.00005900	
C	1.37301200	0.17204200	-0.00003600	
C	2.27488100	1.25618200	0.00023100	
C	1.94308800	-1.11088000	-0.00032100	
C	3.65698700	1.10453900	0.00024000	
H	1.86817000	2.26371800	0.00044800	
C	3.32271600	-1.29676300	-0.00031600	
H	1.30360100	-1.98538400	-0.00056600	
C	4.18276400	-0.19806600	-0.00003300	
H	3.71465500	-2.30604700	-0.00054400	
H	-0.78281400	-1.53534500	-0.00003300	
C	-1.05660900	-0.48162700	-0.00003400	
O	-5.50905600	-2.33233100	0.00048100	
O	-4.97944700	2.43739000	-0.00045000	
O	5.55946500	-0.28632300	-0.00000700	
H	-5.93587400	2.33322500	-0.00040000	
C	4.58680800	2.29138300	0.00053500	
H	5.24199700	2.28349400	0.87746800	
H	5.24204600	2.28389100	-0.87636500	
H	4.02115600	3.22529900	0.00073100	
C	6.14344500	-1.57340000	-0.00025800	
H	5.86435300	-2.14823800	0.89175700	
H	5.86445100	-2.14784700	-0.89255600	
H	7.22233500	-1.41594000	-0.00016500	
**Name of cationic radical**				Energies at B3LYP/6-311G(d,p) in gas phase
C	-5.19192600	0.14086600	-0.00002400	Zero-point correction= 0.282573 (Hartree/Particle)
C	-4.67810700	-1.16855000	0.00000700	Thermal correction to Energy= 0.300593
C	-3.30706600	-1.37704900	0.00000800	Thermal correction to Enthalpy= 0.301538
C	-2.42547900	-0.26914300	0.00001200	Thermal correction to Gibbs Free Energy= 0.235160
C	-2.94988600	1.04351200	-0.00000100	Sum of electronic and zero-point Energies= -844.684185
C	-4.32608900	1.24046200	-0.00002600	Sum of electronic and thermal Energies= -844.666164
H	-6.26877500	0.26968800	-0.00004600	Sum of electronic and thermal Enthalpies= -844.665220
H	-2.90600300	-2.38443000	-0.00004400	Sum of electronic and thermal Free Energies= -844.731598
H	-2.31404400	1.91735600	-0.00000500	
C	-0.00842400	0.40642500	0.00002000	

H	-0.28776700	1.45473500	0.00005100	
C	1.39054100	0.15277200	0.00000400	
C	2.28497900	1.26164200	0.00002800	
C	1.95614200	-1.15456200	-0.00003400	
C	3.65127700	1.11908700	0.00002300	
H	1.86513800	2.26163900	0.00005200	
C	3.31596700	-1.33447400	-0.00003900	
H	1.31615600	-2.02713500	-0.00006500	
C	4.17740300	-0.21137400	-0.00000900	
H	3.72506600	-2.33503500	-0.00007000	
H	-0.74440000	-1.58451900	0.00002400	
C	-1.01676500	-0.53409400	0.00001400	
O	-5.59444600	-2.15914500	0.00003100	
O	-4.76639300	2.51752100	-0.00005200	
O	5.49999100	-0.29287500	-0.00000900	
H	-5.72978000	2.55132500	-0.00005600	
H	-5.16759500	-3.02355700	0.00030300	
C	4.59173100	2.28950500	0.00004900	
H	5.24396400	2.26598500	0.87703700	
H	5.24396800	2.26602000	-0.87693600	
H	4.04144400	3.23007000	0.00006700	
C	6.17043300	-1.56935800	-0.00002800	
H	5.91533300	-2.13423700	0.89876200	
H	5.91536100	-2.13419500	-0.89885200	
H	7.23034000	-1.33172200	-0.00000600	

**Name of compound (10)**	Isorhapontigenin
Cartesian Coordinates optimized at B3LYP/6-311G(d,p)	Energies at B3LYP/6-311G(d,p) in gas phase
C -5.09185000 0.41898800 0.00001500	Zero-point correction= 0.258875 (Hartree/Particle)
C -4.71101000 -0.92170800 0.00010900	Thermal correction to Energy= 0.276466
C -3.36356200 -1.27465100 0.00004100	Thermal correction to Enthalpy= 0.277410
C -2.36504600 -0.28477200 -0.00002600	Thermal correction to Gibbs Free Energy= 0.211851
C -2.75069700 1.06244700 -0.00009000	Sum of electronic and zero-point Energies= -880.868436
C -4.10073700 1.40144500 -0.00010300	Sum of electronic and thermal Energies= -880.850845
H -6.14656900 0.67149800 0.00000800	Sum of electronic and thermal Enthalpies= -880.849901
H -3.08010100 -2.32311700 -0.00012700	Sum of electronic and thermal Free Energies= -880.915460
H -2.02568000 1.86495000 -0.00018200	
C 0.12372500 0.07244300 -0.00003800	
H -0.02173000 1.14964600 0.00002300	
C 1.52596800 -0.34371000 -0.00006200	

C	2.51895900	0.65836300	0.00003200
C	1.94437200	-1.68218500	-0.00016000
C	3.86536200	0.32947400	0.00003500
H	2.21214300	1.69679400	0.00010700
C	3.29408400	-2.01421900	-0.00015900
H	1.21358000	-2.48116500	-0.00023900
C	4.26483300	-1.01948600	-0.00006000
H	3.61784300	-3.04810800	-0.00023600
H	-0.82945000	-1.79604900	-0.00011100
C	-0.96493300	-0.71790200	-0.00006600
O	-5.71373700	-1.85026000	0.00025600
O	-4.40773600	2.73337000	-0.00021900
O	5.58390200	-1.34562500	-0.00005600
H	-5.36467500	2.83681100	-0.00024200
H	-5.32506500	-2.73091100	0.00097000
H	6.08204700	-0.51701300	0.00002300
O	4.91595400	1.21494800	0.00013000
C	4.62932500	2.60747600	0.00024500
H	4.06723600	2.89608600	-0.89422600
H	4.06717400	2.89592500	0.89472900
H	5.59342700	3.11316800	0.00032300

**Name of radical**	**10-O3**

Cartesian Coordinates optimized at B3LYP/6-311G(d,p)				Energies at B3LYP/6-311G(d,p) in gas phase	
C	-5.16815400	0.46971900	0.00003100	Zero-point correction=	0.245405 (Hartree/Particle)
C	-4.78617300	-0.85124000	0.00000600	Thermal correction to Energy=	0.262795
C	-3.41719100	-1.19492900	-0.00001100	Thermal correction to Enthalpy=	0.263739
C	-2.39082000	-0.21862600	0.00000300	Thermal correction to Gibbs Free Energy=	0.197615
C	-2.77158200	1.11594600	0.00002600	Sum of electronic and zero-point Energies=	-880.235065
C	-4.16496700	1.51139900	0.00004000	Sum of electronic and thermal Energies=	-880.217675
H	-6.21221400	0.75359400	0.00004300	Sum of electronic and thermal Enthalpies=	-880.216731
H	-3.13926600	-2.24581200	-0.00004200	Sum of electronic and thermal Free Energies=	-880.282856
H	-2.05330000	1.92545900	0.00003500		
C	0.09874000	0.09610200	0.00000800		
H	-0.03655300	1.17476800	0.00003700		
C	1.49541400	-0.33317900	-0.00000800		
C	2.49697800	0.66066800	0.00000600		
C	1.89958200	-1.67603900	-0.00003700		
C	3.83980100	0.31921000	-0.00000800		
H	2.19998200	1.70187000	0.00002900		
C	3.24553800	-2.02095300	-0.00005100		

H 1.16120200 -2.46804900 -0.00004800<br>C 4.22544100 -1.03467300 -0.00003700<br>H 3.56000600 -3.05758700 -0.00007300<br>H -0.88056400 -1.76125700 -0.00004100<br>C -0.99951600 -0.68133200 -0.00001100<br>O -5.75646000 -1.81047600 -0.00001300<br>O -4.48422900 2.72355200 0.00006100<br>O 5.53926600 -1.37347200 -0.00005100<br>H -5.35023500 -2.68318800 0.00001700<br>H 6.04750100 -0.55074000 -0.00003700<br>O 4.89864200 1.19224300 0.00000300<br>C 4.62819400 2.58901300 0.00003400<br>H 4.06986600 2.88343800 -0.89470000<br>H 4.06987700 2.88340000 0.89478700<br>H 5.59824000 3.08281400 0.00003900		
**Name of radical**	**10-O5**	
Cartesian Coordinates optimized at B3LYP/6-311G(d,p)		Energies at B3LYP/6-311G(d,p) in gas phase
C -5.15173900 0.40438800 -0.00000900<br>C -4.80534800 -1.00291300 0.00001400<br>C -3.39500100 -1.34142900 -0.00001100<br>C -2.40205700 -0.37371100 -0.00004300<br>C -2.80047900 0.98658000 -0.00006400<br>C -4.16238400 1.35852000 -0.00004700<br>H -6.20626300 0.65856200 0.00000600<br>H -3.15389400 -2.39790300 -0.00000900<br>H -2.07215900 1.78701100 -0.00009900<br>C 0.07882200 0.02713900 -0.00002200<br>H -0.08183700 1.10236200 0.00002100<br>C 1.48721600 -0.36439500 -0.00002800<br>C 2.46282700 0.65479200 0.00004900<br>C 1.92567700 -1.69635800 -0.00010300<br>C 3.81404300 0.34776100 0.00005300<br>H 2.13952400 1.68826600 0.00010600<br>C 3.28009000 -2.00650700 -0.00009900<br>H 1.20785800 -2.50691000 -0.00016700<br>C 4.23444400 -0.99557200 -0.00002100<br>H 3.62055300 -3.03486700 -0.00015700<br>H -0.84759300 -1.85869400 -0.00008800<br>C -0.99587800 -0.78304400 -0.00005600<br>O -5.68451800 -1.89061900 0.00005600		Zero-point correction= 0.245730 (Hartree/Particle)<br>Thermal correction to Energy= 0.263020<br>Thermal correction to Enthalpy= 0.263964<br>Thermal correction to Gibbs Free Energy= 0.198373<br>Sum of electronic and zero-point Energies= -880.237419<br>Sum of electronic and thermal Energies= -880.220130<br>Sum of electronic and thermal Enthalpies= -880.219186<br>Sum of electronic and thermal Free Energies= -880.284776

O	-4.40236600	2.70145100	-0.00007100		
O	5.55672000	-1.30049400	-0.00001700		
H	-5.35343700	2.85446700	-0.00006800		
H	6.04362700	-0.46506600	0.00004400		
O	4.85037800	1.24830200	0.00012600		
C	4.54393500	2.63698400	0.00021200		
H	3.97809500	2.91714600	-0.89452500		
H	3.97805700	2.91702600	0.89496300		
H	5.50075100	3.15606600	0.00026700		

**Name of radical**	**10-O4’**

Cartesian Coordinates optimized at B3LYP/6-311G(d,p)				Energies at B3LYP/6-311G(d,p) in gas phase	
C	-5.02194900	0.42158800	0.00000600	Zero-point correction=	0.246035 (Hartree/Particle)
C	-4.64471100	-0.92155600	-0.00000800	Thermal correction to Energy=	0.263276
C	-3.29873100	-1.27599700	-0.00001100	Thermal correction to Enthalpy=	0.264220
C	-2.29959900	-0.28352500	-0.00000100	Thermal correction to Gibbs Free Energy=	0.199297
C	-2.68259900	1.06612500	0.00001400	Sum of electronic and zero-point Energies=	-880.244376
C	-4.03238500	1.40599100	0.00001700	Sum of electronic and thermal Energies=	-880.227134
H	-6.07664100	0.67406600	0.00000900	Sum of electronic and thermal Enthalpies=	-880.226190
H	-3.01460700	-2.32393400	-0.00002000	Sum of electronic and thermal Free Energies=	-880.291113
H	-1.95701000	1.86778500	0.00002200		
C	0.19187100	0.08550700	0.00000000		
H	0.04241700	1.16146000	0.00000900		
C	1.56669400	-0.33139400	-0.00000400		
C	2.57612100	0.66653100	0.00000400		
C	1.96741000	-1.70561500	-0.00001700		
C	3.91501700	0.34227200	0.00000100		
H	2.26256300	1.70315000	0.00001300		
C	3.28177000	-2.05641000	-0.00002000		
H	1.21070700	-2.48067300	-0.00002300		
C	4.35466900	-1.07191700	-0.00001200		
H	3.59600800	-3.09359700	-0.00003000		
H	-0.76876400	-1.79152200	-0.00001400		
C	-0.90771400	-0.71479700	-0.00000500		
O	-5.65050200	-1.84340300	-0.00001800		
O	-4.33772200	2.73585500	0.00003100		
O	5.54885300	-1.38414700	-0.00001500		
H	-5.29424400	2.84442800	0.00003300		
H	-5.27058400	-2.72801200	-0.00003500		
O	4.93034000	1.21963700	0.00000800		
C	4.63451800	2.61193900	0.00002100		

H	4.07108600	2.89679100	-0.89476800	
H	4.07108700	2.89677500	0.89481600	
H	5.59793600	3.11730300	0.00002600	
**Name of anion**	10-O3			
Cartesian Coordinates optimized at B3LYP/6-311G(d,p				Energies at B3LYP/6-311G(d,p) in gas phase
C	-5.13743000	0.44958400	0.00002500	Zero-point correction= 0.244394 (Hartree/Particle)
C	-4.74125400	-0.87369000	0.00000300	Thermal correction to Energy= 0.261932
C	-3.39162800	-1.24099700	-0.00000700	Thermal correction to Enthalpy= 0.262876
C	-2.41236900	-0.21801700	0.00000600	Thermal correction to Gibbs Free Energy= 0.197395
C	-2.80014000	1.11970800	0.00002700	Sum of electronic and zero-point Energies= -880.315426
C	-4.18435200	1.54380800	0.00003500	Sum of electronic and thermal Energies= -880.297889
H	-6.19460000	0.69449300	0.00003600	Sum of electronic and thermal Enthalpies= -880.296945
H	-3.09647000	-2.28797200	-0.00003800	Sum of electronic and thermal Free Energies= -880.362425
H	-2.06475800	1.91808400	0.00003900	
C	0.09088300	0.12845000	-0.00000200	
H	-0.04369300	1.20661300	0.00001300	
C	1.48347200	-0.31562400	-0.00001400	
C	2.50168400	0.66361400	0.00000300	
C	1.89119700	-1.66118500	-0.00004100	
C	3.84298000	0.31082400	-0.00000700	
H	2.21073600	1.70664300	0.00002400	
C	3.23757000	-2.01384100	-0.00005100	
H	1.14728600	-2.44816600	-0.00005500	
C	4.22622700	-1.03854800	-0.00003400	
H	3.54207900	-3.05460200	-0.00007200	
H	-0.88033600	-1.72796300	-0.00002100	
C	-1.01374000	-0.64652600	-0.00000700	
O	-5.73385500	-1.85123800	-0.00000900	
O	-4.53143800	2.75193100	0.00005200	
O	5.55439500	-1.38348200	-0.00004300	
H	-5.28506300	-2.70188200	0.00003300	
H	6.04744500	-0.55301000	-0.00002800	
O	4.90950200	1.19227000	0.00000800	
C	4.62592700	2.58156900	0.00003900	
H	4.06175300	2.87442300	-0.89238500	
H	4.06175700	2.87438400	0.89247900	
H	5.59088700	3.08831500	0.00004800	
**Name of anion**	10-O5			
Cartesian Coordinates optimized at B3LYP/6-311G(d,p				Energies at B3LYP/6-311G(d,p) in gas phase
C	-5.13467600	0.34661200	-0.00003700	Zero-point correction= 0.244730 (Hartree/Particle)

C	-4.80157000	-1.05994300	-0.00006800	Thermal correction to Energy=	0.262111
C	-3.37322200	-1.30825200	-0.00006400	Thermal correction to Enthalpy=	0.263055
C	-2.41220800	-0.29596600	-0.00003500	Thermal correction to Gibbs Free Energy=	0.198045
C	-2.80293900	1.06327100	-0.00000500	Sum of electronic and zero-point Energies=	-880.317556
C	-4.16712400	1.34245800	-0.00000700	Sum of electronic and thermal Energies=	-880.300175
H	-6.19331300	0.60561700	-0.00003800	Sum of electronic and thermal Enthalpies=	-880.299231
H	-3.06468400	-2.35072400	-0.00008700	Sum of electronic and thermal Free Energies=	-880.364241
H	-2.09250800	1.87926000	0.00001800		
C	0.08359700	0.09144100	-0.00000700		
H	-0.06407900	1.16818200	0.00002100		
C	1.48371400	-0.33028600	-0.00000600		
C	2.48740000	0.66356300	0.00004600		
C	1.91054400	-1.66976800	-0.00005400		
C	3.83386800	0.33016400	0.00005100		
H	2.18185200	1.70256500	0.00008300		
C	3.26180400	-2.00268000	-0.00004900		
H	1.17783300	-2.46702600	-0.00009700		
C	4.23648300	-1.01325200	0.00000400		
H	3.58090400	-3.03899000	-0.00008700		
H	-0.86363800	-1.78093200	-0.00006000		
C	-1.00821800	-0.70209300	-0.00003500		
O	-5.65923500	-1.97967000	-0.00009400		
O	-4.53325700	2.68530300	0.00002200		
O	5.56946300	-1.33923500	0.00000900		
H	-5.49519800	2.70234800	0.00001600		
H	6.05090300	-0.50207400	0.00005000		
O	4.88784000	1.22759800	0.00010200		
C	4.58424700	2.61191000	0.00015900		
H	4.01583000	2.89690800	-0.89231600		
H	4.01580300	2.89682900	0.89264300		
H	5.54161200	3.13300000	0.00019600		
**Name of anion**	10-O4’				
Cartesian Coordinates optimized at B3LYP/6-311G(d,p				Energies at B3LYP/6-311G(d,p) in gas phase	
C	-5.04595500	0.44953200	0.00001200	Zero-point correction=	0.244450 (Hartree/Particle)
C	-4.67792800	-0.89675900	-0.00000600	Thermal correction to Energy=	0.261753
C	-3.34437000	-1.27774900	-0.00001200	Thermal correction to Enthalpy=	0.262698
C	-2.30079000	-0.31768400	0.00000000	Thermal correction to Gibbs Free Energy=	0.198529
C	-2.68353900	1.04461000	0.00001900	Sum of electronic and zero-point Energies=	-880.323442
C	-4.02242200	1.40340300	0.00002400	Sum of electronic and thermal Energies=	-880.306139
H	-6.09483600	0.72612200	0.00001600	Sum of electronic and thermal Enthalpies=	-880.305195

H -3.08872100 -2.33480900 -0.00002600<br>H -1.94576200 1.83588400 0.00002900<br>C 0.19292200 0.00776700 0.00000100<br>H 0.03919900 1.08673500 0.00001200<br>C 1.56715300 -0.37883400 -0.00000600<br>C 2.57900400 0.63256800 0.00000300<br>C 2.01882900 -1.72225600 -0.00002100<br>C 3.91848400 0.33587100 -0.00000200<br>H 2.24681000 1.66610700 0.00001400<br>C 3.35529400 -2.03705200 -0.00002600<br>H 1.28803200 -2.52644000 -0.00002700<br>C 4.41365900 -1.04693400 -0.00001800<br>H 3.68584200 -3.07175800 -0.00003700<br>H -0.80957400 -1.85524800 -0.00001900<br>C -0.93084400 -0.77506600 -0.00000600<br>O -5.69904900 -1.82323400 -0.00001800<br>O -4.31716600 2.75064500 0.00004200<br>O 5.62733700 -1.33073600 -0.00002300<br>H -5.27436900 2.84350400 0.00004300<br>H -5.29712500 -2.69758900 -0.00003100<br>O 4.92732300 1.27876200 0.00000600<br>C 4.55054500 2.63135400 0.00002200<br>H 3.96272500 2.89929400 -0.89058700<br>H 3.96272800 2.89927400 0.89063800<br>H 5.47723900 3.20880900 0.00002600	Sum of electronic and thermal Free Energies= -880.369363
**Name of cationic radical**	Energies at B3LYP/6-311G(d,p) in gas phase
C -5.04718400 0.42285600 -0.00001200<br>C -4.68130000 -0.93414700 0.00000300<br>C -3.34043400 -1.29161200 0.00000500<br>C -2.34298800 -0.28782300 0.00000900<br>C -2.71867000 1.07342800 0.00000300<br>C -4.06515900 1.41992000 -0.00001100<br>H -6.10310200 0.67054700 -0.00002700<br>H -3.05223900 -2.33699100 -0.00002500<br>H -1.99101800 1.87243100 0.00000300<br>C 0.13562300 0.10862300 0.00000700<br>H -0.02224100 1.18184600 0.00001500<br>C 1.49848800 -0.30653500 -0.00000100<br>C 2.50939200 0.69361100 0.00000500<br>C 1.89482400 -1.67919200 -0.00001500	Zero-point correction= 0.259760 (Hartree/Particle)<br>Thermal correction to Energy= 0.277093<br>Thermal correction to Enthalpy= 0.278037<br>Thermal correction to Gibbs Free Energy= 0.213250<br>Sum of electronic and zero-point Energies= -880.624086<br>Sum of electronic and thermal Energies= -880.606753<br>Sum of electronic and thermal Enthalpies= -880.605809<br>Sum of electronic and thermal Free Energies= -880.670596

C	3.83973700	0.34478000	0.00000200	
H	2.21531500	1.73471900	0.00001400	
C	3.21943700	-2.03658700	-0.00001900	
H	1.14626900	-2.45948300	-0.00002500	
C	4.20958000	-1.04016500	-0.00000900	
H	3.53391400	-3.07231100	-0.00003000	
H	-0.82133900	-1.78391500	0.00001200	
C	-0.97093300	-0.70933300	0.00000800	
O	-5.69990500	-1.81950700	0.00001500	
O	-4.36299100	2.73806900	-0.00002600	
O	5.48653800	-1.38486300	-0.00001300	
H	-5.31703300	2.87591000	-0.00002800	
H	-5.36918600	-2.72503800	0.00015000	
H	6.03599300	-0.58269200	-0.00000500	
O	4.90600200	1.16589500	0.00000800	
C	4.70551800	2.59021900	0.00002300	
H	4.16538400	2.89739800	-0.89866300	
H	4.16540000	2.89738000	0.89872500	
H	5.70114700	3.02518800	0.00001800	

**Name of compound (11)**	Piceatannol
Cartesian Coordinates optimized at B3LYP/6-311G(d,p)	Energies at B3LYP/6-311G(d,p) in gas phase
C 4.81254300 -0.03128000 0.00000500	Zero-point correction= 0.230348 (Hartree/Particle)
C 4.19209200 1.21674200 -0.00001800	Thermal correction to Energy= 0.246669
C 2.80280300 1.31620700 -0.00003500	Thermal correction to Enthalpy= 0.247613
C 2.00383000 0.15950500 -0.00000700	Thermal correction to Gibbs Free Energy= 0.184965
C 2.62972100 -1.09402900 0.00001900	Sum of electronic and zero-point Energies= -841.586014
C 4.01915600 -1.17933600 0.00001600	Sum of electronic and thermal Energies= -841.569693
H 5.89570700 -0.08559200 0.00001200	Sum of electronic and thermal Enthalpies= -841.568749
H 2.33156700 2.29466500 -0.00009700	Sum of electronic and thermal Free Energies= -841.631397
H 2.06376500 -2.01558100 0.00003700	
C -0.37861800 -0.64628000 -0.00000700	
H -0.04567300 -1.68068300 -0.00000300	
C -1.83311100 -0.48644500 -0.00000700	
C -2.63777300 -1.63488500 -0.00002700	
C -2.47034700 0.76894600 0.00001600	
C -4.02820100 -1.54091000 -0.00002700	
H -2.17183400 -2.61350100 -0.00004400	
C -3.85005500 0.87099400 0.00001700	
H -1.90337200 1.69112900 0.00003600	

C	-4.63644700	-0.29516200	-0.00000500	
H	-4.63781000	-2.43973400	-0.00004300	
H	0.21754600	1.36378500	-0.00002500	
C	0.54777300	0.32849900	-0.00001400	
O	5.00729300	2.31339300	-0.00003600	
O	4.56561200	-2.43197400	0.00003600	
O	-5.99510000	-0.08144500	-0.00000200	
H	-6.46141400	-0.92281800	-0.00001500	
H	5.52529900	-2.35817400	0.00002600	
H	4.46384100	3.10797200	0.00021100	
O	-4.44265900	2.09751500	0.00004000	
H	-5.39897800	1.96322100	0.00003800	

**Name of radical**	**11-O3**
Cartesian Coordinates optimized at B3LYP/6-311G(d,p)	Energies at B3LYP/6-311G(d,p) in gas phase

C	4.89107300	-0.07301800	0.00000600	Zero-point correction= 0.216851 (Hartree/Particle)
C	4.27382100	1.15589800	-0.00003700	Thermal correction to Energy= 0.232989
C	2.86507700	1.24316400	-0.00004400	Thermal correction to Enthalpy= 0.233933
C	2.03583800	0.09524700	0.00000300	Thermal correction to Gibbs Free Energy= 0.169618
C	2.65408600	-1.14718200	0.00004900	Sum of electronic and zero-point Energies= -840.952568
C	4.09604200	-1.28065700	0.00004700	Sum of electronic and thermal Energies= -840.936430
H	5.96949100	-0.16040300	0.00001200	Sum of electronic and thermal Enthalpies= -840.935486
H	2.39925500	2.22522900	-0.00009700	Sum of electronic and thermal Free Energies= -840.999801
H	2.09528500	-2.07393300	0.00009000	
C	-0.35549600	-0.66837800	-0.00000600	
H	-0.03320000	-1.70629500	-0.00001400	
C	-1.80699900	-0.49315400	-0.00000800	
C	-2.62193000	-1.63413600	-0.00006900	
C	-2.43003500	0.76933900	0.00004900	
C	-4.01100800	-1.52595100	-0.00007700	
H	-2.16584900	-2.61727600	-0.00011200	
C	-3.80831700	0.88561700	0.00004100	
H	-1.85380500	1.68579100	0.00010500	
C	-4.60618500	-0.27368300	-0.00002300	
H	-4.62973100	-2.41830300	-0.00012600	
H	0.26870800	1.33513900	-0.00000700	
C	0.58288100	0.29502700	-0.00000200	
O	5.05230900	2.27625400	-0.00009300	
O	4.63190000	-2.41415600	0.00008700	
O	-5.96054100	-0.04578700	-0.00002500	
H	-6.43802200	-0.88106900	-0.00006400	

H	4.49358400	3.06019100	0.00004200		
O	-4.38779500	2.11713400	0.00009700		
H	-5.34579400	1.99457000	0.00008300		
**Name of radical**	**11-O5**				
Cartesian Coordinates optimized at B3LYP/6-311G(d,p)				Energies at B3LYP/6-311G(d,p) in gas phase	
C	-4.87836000	-0.00795900	0.00000400	Zero-point correction=	0.217274 (Hartree/Particle)
C	-4.27953200	1.31162700	0.00002700	Thermal correction to Energy=	0.233259
C	-2.83080800	1.38542800	0.00002700	Thermal correction to Enthalpy=	0.234203
C	-2.03319200	0.25149200	0.00000400	Thermal correction to Gibbs Free Energy=	0.171798
C	-2.67407300	-1.01232500	-0.00001900	Sum of electronic and zero-point Energies=	-840.954859
C	-4.08131100	-1.12772800	-0.00001800	Sum of electronic and thermal Energies=	-840.938875
H	-5.96162100	-0.06404400	0.00000300	Sum of electronic and thermal Enthalpies=	-840.937931
H	-2.39961000	2.37958000	0.00004700	Sum of electronic and thermal Free Energies=	-841.000335
H	-2.10473500	-1.93262300	-0.00004000		
C	0.33301400	-0.59585300	0.00000000		
H	-0.01543000	-1.62529300	-0.00000200		
C	1.78963100	-0.46325300	0.00000000		
C	2.57133000	-1.62720200	0.00001900		
C	2.44836800	0.78081000	-0.00001900		
C	3.96303800	-1.55926600	0.00002100		
H	2.08720800	-2.59699400	0.00003300		
C	3.82962400	0.85708900	-0.00001700		
H	1.89863900	1.71333700	-0.00003700		
C	4.59388700	-0.32461300	0.00000400		
H	4.55591700	-2.46902100	0.00003700		
H	-0.23278000	1.42675300	0.00001100		
C	-0.57527900	0.39631400	0.00000500		
O	-4.98025200	2.34597700	0.00004600		
O	-4.56387700	-2.40345200	-0.00004100		
O	5.95443200	-0.13568900	0.00000400		
H	6.40764900	-0.98433300	0.00001600		
H	-5.52688500	-2.37933700	-0.00003600		
O	4.44412300	2.07106300	-0.00003500		
H	5.39822600	1.92141000	-0.00003100		
**Name of radical**	**11-O3’**				
Cartesian Coordinates optimized at B3LYP/6-311G(d,p)				Energies at B3LYP/6-311G(d,p) in gas phase	
C	-4.76952700	-0.04880100	-0.00244500	Zero-point correction=	0.218588 (Hartree/Particle)
C	-4.16371300	1.20226800	-0.10973900	Thermal correction to Energy=	0.234189
C	-2.77546800	1.31684700	-0.10440700	Thermal correction to Enthalpy=	0.235133
C	-1.96777500	0.17156000	0.00609200	Thermal correction to Gibbs Free Energy=	0.173036

C -2.57819700 -1.08414900 0.11816100<br>C -3.96697200 -1.18479600 0.11154400<br>H -5.85206700 -0.11404800 -0.00634900<br>H -2.31281000 2.29535700 -0.19007100<br>H -2.00214200 -1.99356600 0.22110100<br>C 0.42238000 -0.61161700 -0.01010400<br>H 0.10359200 -1.64964700 -0.03694400<br>C 1.87265600 -0.41729900 -0.01098000<br>C 2.69122000 -1.57817900 -0.21646500<br>C 2.49074000 0.80536900 0.17561200<br>C 4.07464600 -1.52678000 -0.25163400<br>H 2.19728700 -2.53383700 -0.35363100<br>C 3.91527200 0.92942300 0.14857100<br>H 1.93065700 1.71364300 0.35820900<br>C 4.69565100 -0.29621500 -0.07538900<br>H 4.67137400 -2.41646200 -0.41056700<br>H -0.19163600 1.39141400 -0.01194600<br>C -0.51517400 0.35403100 -0.00015600<br>O -4.99139200 2.28193500 -0.21673400<br>O -4.49960600 -2.43621600 0.22476000<br>O 6.01346600 -0.13922900 -0.09071600<br>H 6.13798800 0.82428900 0.06308300<br>H -5.46033100 -2.37650700 0.21255600<br>H -4.46093600 3.08247700 -0.28485600<br>O 4.56310500 1.99187400 0.30362000		Sum of electronic and zero-point Energies= -840.969029<br>Sum of electronic and thermal Energies= -840.953428<br>Sum of electronic and thermal Enthalpies= -840.952484<br>Sum of electronic and thermal Free Energies= -841.014580
**Name of radical**	**11-O4'**	
Cartesian Coordinates optimized at B3LYP/6-311G(d,p)		Energies at B3LYP/6-311G(d,p) in gas phase
C 4.74597600 -0.03654100 0.00000400<br>C 4.13462400 1.21778400 0.00000300<br>C 2.74701900 1.32498000 0.00000100<br>C 1.94307300 0.16863900 -0.00000200<br>C 2.56104200 -1.09083900 0.00000100<br>C 3.94993000 -1.18319600 0.00000500<br>H 5.82885700 -0.09522200 0.00000300<br>H 2.27885600 2.30446700 0.00000400<br>H 1.99026800 -2.00912500 0.00000000<br>C -0.44350100 -0.63847100 -0.00000400<br>H -0.11141100 -1.67228600 -0.00000600<br>C -1.87023000 -0.46277100 -0.00000400<br>C -2.68754100 -1.64274900 -0.00000900		Zero-point correction= 0.218614 (Hartree/Particle)<br>Thermal correction to Energy= 0.234133<br>Thermal correction to Enthalpy= 0.235077<br>Thermal correction to Gibbs Free Energy= 0.173885<br>Sum of electronic and zero-point Energies= -840.975790<br>Sum of electronic and thermal Energies= -840.960271<br>Sum of electronic and thermal Enthalpies= -840.959326<br>Sum of electronic and thermal Free Energies= -841.020519

C	-2.50215600	0.80483500	0.00000300	
C	-4.05081300	-1.58678600	-0.00000700	
H	-2.18767700	-2.60563800	-0.00001400	
C	-3.87090100	0.88642900	0.00000500	
H	-1.92714900	1.72188100	0.00000700	
C	-4.72692500	-0.31295600	-0.00000100	
H	-4.66478300	-2.47919200	-0.00001100	
H	0.16650400	1.37777100	0.00000000	
C	0.49727000	0.34346400	-0.00000200	
O	4.96002500	2.30360900	0.00001500	
O	4.48871500	-2.43608000	0.00000500	
O	-5.96183800	-0.14482300	0.00000100	
H	5.44931100	-2.37240100	0.00000900	
H	4.42959700	3.10714500	-0.00021100	
O	-4.52924000	2.04977400	0.00001100	
H	-5.47485600	1.79182300	0.00001100	
**Name of anion**	11-O3			
Cartesian Coordinates optimized at B3LYP/6-311G(d,p				Energies at B3LYP/6-311G(d,p) in gas phase
C	4.85584400	-0.05505700	0.00000700	Zero-point correction= 0.215560 (Hartree/Particle)
C	4.22224000	1.17252500	0.00001600	Thermal correction to Energy= 0.232063
C	2.82833600	1.28440600	0.00001500	Thermal correction to Enthalpy= 0.233007
C	2.05481400	0.09798000	0.00000400	Thermal correction to Gibbs Free Energy= 0.169671
C	2.68258400	-1.14538300	-0.00000600	Sum of electronic and zero-point Energies= -841.032452
C	4.12141800	-1.30664000	-0.00000600	Sum of electronic and thermal Energies= -841.015949
H	5.94005000	-0.10058700	0.00001000	Sum of electronic and thermal Enthalpies= -841.015005
H	2.34507100	2.25881100	0.00001700	Sum of electronic and thermal Free Energies= -841.078342
H	2.10630200	-2.06508300	-0.00001500	
C	-0.34439600	-0.70026500	-0.00000500	
H	-0.02168800	-1.73749100	-0.00001100	
C	-1.79308100	-0.51172600	-0.00000600	
C	-2.62983900	-1.64286700	-0.00001300	
C	-2.41906300	0.75299700	0.00000100	
C	-4.01819800	-1.52297000	-0.00001400	
H	-2.18128100	-2.62983100	-0.00001800	
C	-3.79772600	0.87767200	-0.00000100	
H	-1.83471700	1.66450100	0.00000600	
C	-4.60668400	-0.26783600	-0.00000800	
H	-4.64411500	-2.41230400	-0.00002000	
H	0.27334300	1.30113600	0.00000900	
C	0.60185400	0.26216900	0.00000300	

O	5.01763900	2.31624400	0.00002700	
O	4.68610200	-2.42955000	-0.00001600	
O	-5.97601900	-0.03588900	-0.00000900	
H	-6.43461100	-0.88047900	-0.00001500	
H	4.41971300	3.06960200	0.00005400	
O	-4.37587800	2.12105000	0.00000600	
H	-5.33144500	1.98685100	0.00000300	

**Name of anion**	11-O5
Cartesian Coordinates optimized at B3LYP/6-311G(d,p	Energies at B3LYP/6-311G(d,p) in gas phase

C	-4.84246200	0.04381300	-0.00000100	Zero-point correction= 0.215939 (Hartree/Particle)
C	-4.25675100	1.36532400	0.00000100	Thermal correction to Energy= 0.232262
C	-2.80729400	1.34700900	0.00000100	Thermal correction to Enthalpy= 0.233206
C	-2.04860900	0.17516700	0.00000000	Thermal correction to Gibbs Free Energy= 0.170496
C	-2.68228100	-1.08933400	-0.00000200	Sum of electronic and zero-point Energies= -841.034538
C	-4.07442100	-1.11303300	-0.00000200	Sum of electronic and thermal Energies= -841.018215
H	-5.93066800	-0.01599500	-0.00000100	Sum of electronic and thermal Enthalpies= -841.017271
H	-2.31253000	2.31497900	0.00000200	Sum of electronic and thermal Free Energies= -841.079981
H	-2.13332500	-2.02153400	-0.00000300	
C	0.33578400	-0.66066100	-0.00000100	
H	-0.00019900	-1.69395000	-0.00000300	
C	1.78807900	-0.49686100	0.00000000	
C	2.60614200	-1.64138000	-0.00000300	
C	2.43377200	0.75764500	0.00000300	
C	3.99647100	-1.54428100	-0.00000300	
H	2.14198200	-2.62127500	-0.00000600	
C	3.81441000	0.85977500	0.00000300	
H	1.86371400	1.67807800	0.00000500	
C	4.60484500	-0.29885300	0.00000000	
H	4.60810800	-2.44354200	-0.00000500	
H	-0.25598200	1.35218300	0.00000200	
C	-0.59449600	0.31762400	0.00000000	
O	-4.93118700	2.42676200	0.00000200	
O	-4.68124600	-2.36558300	-0.00000400	
O	5.97771900	-0.08897600	0.00000100	
H	6.42278200	-0.94074100	-0.00000300	
H	-5.62997000	-2.20577800	-0.00000300	
O	4.41236700	2.09299800	0.00000600	
H	5.36573500	1.94424900	0.00000500	

**Name of anion**	11-O3’
Cartesian Coordinates optimized at B3LYP/6-311G(d,p	Energies at B3LYP/6-311G(d,p) in gas phase

C	-4.78282300	-0.04911200	-0.00001300
C	-4.16973700	1.20348600	0.00006400
C	-2.78611400	1.32250600	0.00008400
C	-1.95344500	0.18132500	0.00002800
C	-2.57943000	-1.08172700	-0.00004900
C	-3.96376600	-1.18173400	-0.00006800
H	-5.86532300	-0.12049900	-0.00002900
H	-2.33066500	2.30942400	0.00014300
H	-2.00141000	-1.99602400	-0.00009700
C	0.43707100	-0.59733700	-0.00000200
H	0.10335200	-1.63396400	-0.00005500
C	1.87764700	-0.44294500	0.00001200
C	2.64969000	-1.61953600	0.00004000
C	2.52412500	0.81867700	0.00000500
C	4.05014000	-1.55608500	0.00007700
H	2.15087100	-2.58405700	0.00000000
C	3.92545700	0.93615400	-0.00010700
H	1.94758700	1.73760600	-0.00006900
C	4.66528500	-0.31964300	0.00013800
H	4.65513500	-2.45818600	0.00012100
H	-0.18741100	1.40925100	0.00010800
C	-0.51158700	0.37262600	0.00004900
O	-4.99611200	2.30383300	0.00011700
O	-4.50652400	-2.44691100	-0.00014600
O	6.00325100	-0.12919300	0.00019300
H	6.01074000	0.87147000	0.00013400
H	-5.46455800	-2.36158700	-0.00015400
H	-4.43986300	3.08928800	0.00016200
O	4.64019400	1.99944000	-0.00039100

Zero-point correction= 0.216977 (Hartree/Particle)
Thermal correction to Energy= 0.232490
Thermal correction to Enthalpy= 0.233435
Thermal correction to Gibbs Free Energy= 0.172969
Sum of electronic and zero-point Energies= -841.054372
Sum of electronic and thermal Energies= -841.038859
Sum of electronic and thermal Enthalpies= -841.037915
Sum of electronic and thermal Free Energies= -841.098381

**Name of anion**	11-O4’

Cartesian Coordinates optimized at B3LYP/6-311G(d,p

C	4.78512800	-0.05507900	0.00000000
C	4.17427300	1.19946800	0.00000100
C	2.79254000	1.32530600	0.00000000
C	1.94892500	0.18773800	-0.00000100
C	2.57568300	-1.07894800	-0.00000100
C	3.95848500	-1.18322400	-0.00000100
H	5.86721100	-0.13120300	0.00000100
H	2.34374200	2.31567300	-0.00000100
H	1.99654100	-1.99265500	-0.00000300

Energies at B3LYP/6-311G(d,p) in gas phase

Zero-point correction= 0.217313 (Hartree/Particle)
Thermal correction to Energy= 0.232836
Thermal correction to Enthalpy= 0.233780
Thermal correction to Gibbs Free Energy= 0.173568
Sum of electronic and zero-point Energies= -841.061967
Sum of electronic and thermal Energies= -841.046445
Sum of electronic and thermal Enthalpies= -841.045501
Sum of electronic and thermal Free Energies= -841.105712

C -0.44187500 -0.59257600 0.00000000<br>H -0.10110100 -1.62723400 0.00000200<br>C -1.86690400 -0.45412800 0.00000000<br>C -2.66930300 -1.61782000 0.00000300<br>C -2.54146700 0.80740900 -0.00000200<br>C -4.05332100 -1.57427900 0.00000300<br>H -2.16819100 -2.58368000 0.00000400<br>C -3.90623600 0.86088400 -0.00000100<br>H -1.98095300 1.73637400 -0.00000400<br>C -4.75021700 -0.33121900 0.00000100<br>H -4.64479200 -2.48468600 0.00000500<br>H 0.19403700 1.42073200 -0.00000200<br>C 0.51467600 0.38240600 -0.00000100<br>O 5.00522900 2.29864000 0.00000300<br>O 4.49776400 -2.45166200 -0.00000200<br>O -6.00564600 -0.15385600 0.00000100<br>H 5.45574600 -2.36671000 -0.00000300<br>H 4.44921100 3.08414700 0.00000800<br>O -4.62156500 2.02097400 -0.00000300<br>H -5.54002700 1.66084400 -0.00000200	
**Name of cationic radical**	Energies at B3LYP/6-311G(d,p) in gas phase
C 4.76298300 -0.04814500 0.00001300<br>C 4.16554300 1.22677500 -0.00000100<br>C 2.78428500 1.34587200 0.00000200<br>C 1.97713400 0.18268600 -0.00001000<br>C 2.58516000 -1.09464400 -0.00000800<br>C 3.97072900 -1.20259500 0.00001200<br>H 5.84601200 -0.10673800 0.00001900<br>H 2.31787900 2.32457100 0.00004700<br>H 2.00647400 -2.00728400 -0.00001600<br>C -0.39236500 -0.64324800 -0.00000900<br>H -0.05443800 -1.67370700 -0.00001400<br>C -1.80734300 -0.46696200 -0.00000600<br>C -2.61856800 -1.64236400 -0.00002000<br>C -2.44554500 0.79682900 0.00001000<br>C -3.99272500 -1.56079500 -0.00001800<br>H -2.13960800 -2.61372200 -0.00003200<br>C -3.81764300 0.89277300 0.00001200<br>H -1.88260400 1.72021100 0.00002200<br>C -4.60445500 -0.30160700 -0.00000200	Zero-point correction= 0.231235 (Hartree/Particle)<br>Thermal correction to Energy= 0.247129<br>Thermal correction to Enthalpy= 0.248074<br>Thermal correction to Gibbs Free Energy= 0.186570<br>Sum of electronic and zero-point Energies= -841.336234<br>Sum of electronic and thermal Energies= -841.320340<br>Sum of electronic and thermal Enthalpies= -841.319396<br>Sum of electronic and thermal Free Energies= -841.380899

H	-4.60260700	-2.45744300	-0.00002900	
H	0.21530100	1.38719800	0.00000000	
C	0.55504400	0.35655100	-0.00000600	
O	5.01691100	2.27193400	0.00000700	
O	4.49338800	-2.44748200	0.00002000	
O	-5.92734400	-0.09259900	0.00000100	
H	-6.43291900	-0.91602200	-0.00000800	
H	5.45702800	-2.41941800	0.00003900	
H	4.53795600	3.10888000	-0.00030700	
O	-4.41148900	2.09485100	0.00002700	
H	-5.37360800	1.99308500	0.00002600	

**Name of compound (12)**	Oxyresveratrol
Cartesian Coordinates optimized at B3LYP/6-311G(d,p)	Energies at B3LYP/6-311G(d,p) in gas phase
C 4.66801300 0.13774300 -0.00001100	Zero-point correction= 0.230250 (Hartree/Particle)
C 4.16479300 -1.16181800 0.00007300	Thermal correction to Energy= 0.246626
C 2.79073300 -1.38916700 0.00008100	Thermal correction to Enthalpy= 0.247571
C 1.88774700 -0.31120300 0.00000500	Thermal correction to Gibbs Free Energy= 0.184017
C 2.39606300 0.99472900 -0.00007900	Sum of electronic and zero-point Energies= -841.584261
C 3.77157900 1.20755900 -0.00008600	Sum of electronic and thermal Energies= -841.567885
H 5.74157500 0.29130600 -0.00001600	Sum of electronic and thermal Enthalpies= -841.566941
H 2.41212000 -2.40725800 0.00014700	Sum of electronic and thermal Free Energies= -841.630494
H 1.74631200 1.85932100 -0.00014200	
C -0.55327100 0.28128300 -0.00003000	
H -0.31218300 1.33754400 -0.00006100	
C -1.98505100 -0.00970000 -0.00003200	
C -2.91090600 1.05546700 0.00008800	
C -2.52470500 -1.30799500 -0.00015800	
C -4.28712700 0.82354700 0.00009100	
C -3.88616000 -1.55702700 -0.00015200	
H -1.84874600 -2.15444800 -0.00027700	
C -4.77757900 -0.48117100 -0.00002400	
H -4.97527300 1.66598500 0.00017800	
H -4.27832800 -2.56586200 -0.00025400	
H 0.22090800 -1.67383400 0.00008100	
C 0.45327100 -0.61214100 0.00001800	
O 5.07766900 -2.17965400 0.00014500	
H 4.60851500 -3.02016100 0.00019800	
O 4.20086900 2.50541400 -0.00017000	
H 5.16325500 2.51934100 -0.00016700	

O -6.11152400 -0.76430600 -0.00002600<br>H -6.61508600 0.05611300 0.00006300<br>O -2.41292000 2.32776700 0.00019500<br>H -3.14020700 2.95754900 0.00038900	
**Name of radical**	**12-O3**
Cartesian Coordinates optimized at B3LYP/6-311G(d,p)	Energies at B3LYP/6-311G(d,p) in gas phase
C 4.74480500 0.18463800 0.00000100<br>C 4.24405000 -1.09566700 0.00022900<br>C 2.84931500 -1.31367700 0.00019600<br>C 1.91609100 -0.24862900 -0.00006500<br>C 2.41651600 1.04636300 -0.00032300<br>C 3.84015700 1.31323700 -0.00028700<br>H 5.81036500 0.37226000 0.00002400<br>H 2.47743200 -2.33523400 0.00038200<br>H 1.77268100 1.91629000 -0.00057500<br>C -0.53025200 0.29860300 -0.00003800<br>H -0.30045500 1.35766000 0.00002100<br>C -1.95730400 -0.00649800 -0.00005900<br>C -2.89202400 1.05146700 0.00027900<br>C -2.48360100 -1.31060000 -0.00041500<br>C -4.26579400 0.80665100 0.00027600<br>C -3.84183200 -1.57174600 -0.00042300<br>H -1.79986100 -2.15083900 -0.00072300<br>C -4.74323100 -0.50296500 -0.00007100<br>H -4.96190000 1.64222800 0.00055100<br>H -4.22563900 -2.58368000 -0.00071000<br>H 0.27361000 -1.64700400 -0.00003300<br>C 0.48804800 -0.58174300 -0.00005700<br>O 5.12271300 -2.14025300 0.00048000<br>H 4.63752700 -2.97165000 0.00066300<br>O 4.26910600 2.49122400 -0.00050700<br>O -6.07223800 -0.79974700 -0.00009300<br>H -6.58665600 0.01409500 0.00015700<br>O -2.40374800 2.32580800 0.00062100<br>H -3.13343700 2.95300300 0.00078400	Zero-point correction= 0.216812 (Hartree/Particle)<br>Thermal correction to Energy= 0.232967<br>Thermal correction to Enthalpy= 0.233911<br>Thermal correction to Gibbs Free Energy= 0.170452<br>Sum of electronic and zero-point Energies= -840.951103<br>Sum of electronic and thermal Energies= -840.934948<br>Sum of electronic and thermal Enthalpies= -840.934004<br>Sum of electronic and thermal Free Energies= -840.997463
**Name of radical**	**12-O5**
Cartesian Coordinates optimized at B3LYP/6-311G(d,p)	Energies at B3LYP/6-311G(d,p) in gas phase
C -4.72719600 0.11583600 0.00000500<br>C -4.25156800 -1.25312500 -0.00002700<br>C -2.81609900 -1.45967400 -0.00003000	Zero-point correction= 0.217190 (Hartree/Particle)<br>Thermal correction to Energy= 0.233218<br>Thermal correction to Enthalpy= 0.234163

C	-1.91746600	-0.40385900	-0.00000600	Thermal correction to Gibbs Free Energy= 0.171293
C	-2.43988900	0.91403400	0.00002600	Sum of electronic and zero-point Energies= -840.953764
C	-3.83045600	1.15765600	0.00002900	Sum of electronic and thermal Energies= -840.937736
H	-5.80077500	0.27073400	0.00000800	Sum of electronic and thermal Enthalpies= -840.936792
H	-2.47762200	-2.48926200	-0.00005500	Sum of electronic and thermal Free Energies= -840.999661
H	-1.78708500	1.77721000	0.00005200	
C	0.50990500	0.23410800	0.00001300	
H	0.25158600	1.28649100	0.00003300	
C	1.94608700	-0.02759600	0.00001200	
C	2.84901900	1.05698100	-0.00003000	
C	2.51045400	-1.31576800	0.00005500	
C	4.22936700	0.85319800	-0.00003200	
C	3.87582300	-1.53654200	0.00005300	
H	1.85168100	-2.17555500	0.00009800	
C	4.74530800	-0.44184500	0.00000800	
H	4.90070800	1.70889600	-0.00006700	
H	4.28887000	-2.53687400	0.00008900	
H	-0.23270600	-1.73721600	-0.00004700	
C	-0.47901600	-0.67949700	-0.00001300	
O	-5.04549100	-2.21867000	-0.00005200	
O	-4.19519300	2.47231900	0.00005800	
H	-5.15641200	2.53509400	0.00005900	
O	6.08269400	-0.69896300	0.00000800	
H	6.57257200	0.12982600	-0.00002300	
O	2.32382200	2.31819200	-0.00007000	
H	3.03688900	2.96419300	-0.00009500	

**Name of radical**	**12-O2’**

Cartesian Coordinates optimized at B3LYP/6-311G(d,p)				Energies at B3LYP/6-311G(d,p) in gas phase
C	-4.63025400	0.15243100	0.00002400	Zero-point correction= 0.217746 (Hartree/Particle)
C	-4.13832100	-1.15369000	0.00013500	Thermal correction to Energy= 0.233530
C	-2.76773500	-1.39002200	0.00015300	Thermal correction to Enthalpy= 0.234474
C	-1.85917500	-0.31226500	0.00006200	Thermal correction to Gibbs Free Energy= 0.172939
C	-2.35692300	1.00027700	-0.00004800	Sum of electronic and zero-point Energies= -840.964367
C	-3.73128200	1.22085500	-0.00006800	Sum of electronic and thermal Energies= -840.948583
H	-5.70307200	0.31107200	0.00001200	Sum of electronic and thermal Enthalpies= -840.947639
H	-2.39258600	-2.40895400	0.00023600	Sum of electronic and thermal Free Energies= -841.009174
H	-1.69977500	1.85882000	-0.00012000	
C	0.58479300	0.28854400	-0.00001600	
H	0.36398400	1.35070700	-0.00014600	
C	1.97927700	-0.00085900	0.00001100	

C 2.90533000 1.16685700 -0.00027400<br>C 2.53400500 -1.30064900 0.00029300<br>C 4.32807400 0.88612100 -0.00025400<br>C 3.89019300 -1.50694200 0.00030000<br>H 1.87908600 -2.16413200 0.00051900<br>C 4.79480500 -0.39509600 0.00002100<br>H 4.99272400 1.74354300 -0.00046700<br>H 4.31275200 -2.50403000 0.00051900<br>H -0.20112900 -1.67637800 0.00018900<br>C -0.43743800 -0.61572500 0.00008500<br>O -5.06203200 -2.15838800 0.00022000<br>H -4.60770200 -3.00715000 0.00031500<br>O -4.15260100 2.51751300 -0.00017700<br>H -5.11492400 2.54271800 -0.00018300<br>O 6.11200900 -0.73197700 0.00005800<br>H 6.64494900 0.07131100 -0.00014000<br>O 2.47182400 2.32828200 -0.00051200		
**Name of radical**	**12-O4'**	
Cartesian Coordinates optimized at B3LYP/6-311G(d,p)		Energies at B3LYP/6-311G(d,p) in gas phase
C 4.58989100 0.14797300 0.00000100<br>C 4.09785100 -1.15826000 -0.00000100<br>C 2.72708400 -1.39466000 -0.00000300<br>C 1.81796700 -0.31723800 -0.00000300<br>C 2.31668600 0.99524500 -0.00000100<br>C 3.69081200 1.21611200 0.00000100<br>H 5.66267400 0.30655200 0.00000200<br>H 2.35224600 -2.41359700 -0.00000400<br>H 1.66167300 1.85543400 -0.00000200<br>C -0.62732500 0.28232600 0.00000500<br>H -0.38435200 1.33751200 0.00001300<br>C -2.02056300 -0.01558100 0.00000200<br>C -2.97327800 1.07029800 -0.00000100<br>C -2.55183600 -1.34478400 0.00000400<br>C -4.31878800 0.84163300 -0.00000300<br>C -3.88758800 -1.59444500 0.00000300<br>H -1.85838800 -2.17698100 0.00000700<br>C -4.86627800 -0.50515400 0.00000000<br>H -5.03273200 1.65928400 -0.00000600<br>H -4.28311700 -2.60301500 0.00000400<br>H 0.16290400 -1.68303700 -0.00001300		Zero-point correction= 0.217782 (Hartree/Particle)<br>Thermal correction to Energy= 0.233595<br>Thermal correction to Enthalpy= 0.234539<br>Thermal correction to Gibbs Free Energy= 0.172957<br>Sum of electronic and zero-point Energies= -840.963473<br>Sum of electronic and thermal Energies= -840.947660<br>Sum of electronic and thermal Enthalpies= -840.946716<br>Sum of electronic and thermal Free Energies= -841.008298

C	0.39749500	-0.62314800	-0.00000400
O	5.02133200	-2.16212500	0.00000000
H	4.56846000	-3.01174100	-0.00000400
O	4.11121800	2.51326500	0.00000200
H	5.07357000	2.53929000	0.00000400
O	-6.08493700	-0.72374900	-0.00000100
O	-2.43803700	2.32478700	-0.00000400
H	-3.15233100	2.97098500	0.00000200

**Name of anion**	12-O3

Cartesian Coordinates optimized at B3LYP/6-311G(d,p	Energies at B3LYP/6-311G(d,p) in gas phase

C	4.71753300	0.14356700	-0.01099900
C	4.18237400	-1.12391400	0.11136100
C	2.80178800	-1.34590300	0.11623500
C	1.93679100	-0.23090500	-0.00738200
C	2.46440600	1.05154400	-0.13390900
C	3.88637800	1.32601200	-0.14214900
H	5.79474400	0.27509900	-0.01207100
H	2.39741100	-2.35091400	0.21531200
H	1.81576500	1.91561300	-0.23642300
C	-0.52021200	0.37144100	-0.04787300
H	-0.29180500	1.42958000	-0.07639100
C	-1.94396600	0.03794900	-0.04953200
C	-2.90966600	1.05357200	0.13349600
C	-2.45428600	-1.25928300	-0.23621300
C	-4.27773000	0.77614800	0.14119100
C	-3.81214200	-1.55528300	-0.22693400
H	-1.75327900	-2.06696000	-0.40944400
C	-4.73355300	-0.52957600	-0.03609300
H	-4.98885300	1.58733900	0.29216800
H	-4.16955000	-2.56679600	-0.37747500
H	0.25865400	-1.57039800	0.08132300
C	0.50070400	-0.51027700	0.00587200
O	5.06520400	-2.19590700	0.23071300
H	4.52722300	-2.98941600	0.30791400
O	4.36148300	2.48442400	-0.25446500
O	-6.07405200	-0.84617500	-0.03599500
H	-6.57707700	-0.03722700	0.09682900
O	-2.45659800	2.33519800	0.31158900
H	-3.21204000	2.92319000	0.40110000

Zero-point correction=	0.215642 (Hartree/Particle)
Thermal correction to Energy=	0.232002
Thermal correction to Enthalpy=	0.232947
Thermal correction to Gibbs Free Energy=	0.170144
Sum of electronic and zero-point Energies=	-841.027778
Sum of electronic and thermal Energies=	-841.011418
Sum of electronic and thermal Enthalpies=	-841.010474
Sum of electronic and thermal Free Energies=	-841.073276

**Name of anion**	12-O5

Cartesian Coordinates optimized at B3LYP/6-311G(d,p	Energies at B3LYP/6-311G(d,p) in gas phase
C -4.70709600 0.04352300 0.00403400<br>C -4.22836600 -1.31693400 -0.08937600<br>C -2.78228800 -1.41371800 -0.08913200<br>C -1.93248600 -0.30913900 -0.00640900<br>C -2.46337300 0.99846600 0.08421000<br>C -3.84926400 1.13262200 0.08608400<br>H -5.78715600 0.18961100 0.00921200<br>H -2.36665300 -2.41599600 -0.15791900<br>H -1.84012500 1.87991500 0.15500800<br>C 0.51258500 0.33116100 0.03256000<br>H 0.26967200 1.38605000 0.06196900<br>C 1.94259700 0.02377700 0.03422900<br>C 2.89142500 1.06312300 -0.09066700<br>C 2.47453800 -1.27195300 0.16392200<br>C 4.26451900 0.81117200 -0.09500000<br>C 3.83735300 -1.54245800 0.15827500<br>H 1.78710900 -2.10052500 0.28362500<br>C 4.74229200 -0.49281000 0.02716900<br>H 4.96230700 1.64129200 -0.19837700<br>H 4.21090100 -2.55392500 0.26242800<br>H -0.24311200 -1.62530700 -0.08031600<br>C -0.49385800 -0.56775700 -0.01671300<br>O -4.98525100 -2.31922900 -0.16339400<br>O -4.35602200 2.42613700 0.17629800<br>H -5.31425100 2.34082000 0.16596500<br>O 6.08819100 -0.78534100 0.02818100<br>H 6.57784500 0.03699500 -0.06690300<br>O 2.41871200 2.34594900 -0.21337200<br>H 3.16695400 2.94649700 -0.27549800	Zero-point correction= 0.215972 (Hartree/Particle)<br>Thermal correction to Energy= 0.232204<br>Thermal correction to Enthalpy= 0.233148<br>Thermal correction to Gibbs Free Energy= 0.170567<br>Sum of electronic and zero-point Energies= -841.030386<br>Sum of electronic and thermal Energies= -841.014154<br>Sum of electronic and thermal Enthalpies= -841.013210<br>Sum of electronic and thermal Free Energies= -841.075791
**Name of anion**	12-O2’
Cartesian Coordinates optimized at B3LYP/6-311G(d,p	Energies at B3LYP/6-311G(d,p) in gas phase
C -4.65482000 0.16961100 0.00001200<br>C -4.16062500 -1.13420000 0.00018200<br>C -2.79551000 -1.38399200 0.00020500<br>C -1.85358600 -0.32675800 0.00005900<br>C -2.36129600 0.99132600 -0.00011200<br>C -3.72905300 1.21928000 -0.00013400<br>H -5.72569200 0.34356600 -0.00000400<br>H -2.43740800 -2.41111400 0.00033700	Zero-point correction= 0.216354 (Hartree/Particle)<br>Thermal correction to Energy= 0.232299<br>Thermal correction to Enthalpy= 0.233243<br>Thermal correction to Gibbs Free Energy= 0.172219<br>Sum of electronic and zero-point Energies= -841.049389<br>Sum of electronic and thermal Energies= -841.033445<br>Sum of electronic and thermal Enthalpies= -841.032501<br>Sum of electronic and thermal Free Energies= -841.093524

H	-1.69648200	1.84463500	-0.00023000
C	0.59426900	0.23989700	-0.00003800
H	0.37423300	1.30567600	-0.00018600
C	2.00542000	-0.00979300	-0.00001200
C	2.88887600	1.17183100	-0.00020500
C	2.57079600	-1.29927400	0.00018900
C	4.30782000	0.87377700	-0.00017000
C	3.93338800	-1.53080100	0.00021400
H	1.90523400	-2.15893800	0.00033300
C	4.79456900	-0.41123900	0.00002900
H	4.98205000	1.72853400	-0.00031200
H	4.35369000	-2.52920300	0.00037100
H	-0.22156400	-1.71439900	0.00022900
C	-0.44026500	-0.64819300	0.00009000
O	-5.08732400	-2.15614900	0.00032400
H	-4.60139300	-2.98671900	0.00044500
O	-4.15447000	2.53009300	-0.00030500
H	-5.11613700	2.52937900	-0.00030200
O	6.15338100	-0.67891700	0.00006000
H	6.60276000	0.17239700	-0.00007800
O	2.46101400	2.34839400	-0.00038700

**Name of anion**	12-O4'

Cartesian Coordinates optimized at B3LYP/6-311G(d,p

C	4.63016200	0.16924900	-0.00000100
C	4.13706300	-1.13583800	-0.00000500
C	2.77303100	-1.38899200	-0.00000300
C	1.82636100	-0.33435900	0.00000300
C	2.33474300	0.98515500	0.00000600
C	3.70167800	1.21598300	0.00000400
H	5.70059900	0.34504400	-0.00000100
H	2.41806800	-2.41708800	-0.00000600
H	1.67260800	1.84069500	0.00001100
C	-0.62157300	0.23056500	0.00000100
H	-0.37183200	1.28737500	-0.00000300
C	-2.02483100	-0.03287300	0.00000100
C	-2.96569900	1.03947900	-0.00000500
C	-2.59792600	-1.33942100	0.00000600
C	-4.32543100	0.83496500	-0.00000500
C	-3.94510600	-1.57045000	0.00000600
H	-1.92144100	-2.18961300	0.00001000

Energies at B3LYP/6-311G(d,p) in gas phase

Zero-point correction=	0.216529 (Hartree/Particle)
Thermal correction to Energy=	0.232492
Thermal correction to Enthalpy=	0.233436
Thermal correction to Gibbs Free Energy=	0.172408
Sum of electronic and zero-point Energies=	-841.049687
Sum of electronic and thermal Energies=	-841.033723
Sum of electronic and thermal Enthalpies=	-841.032779
Sum of electronic and thermal Free Energies=	-841.093807

C	-4.92091100	-0.48628000	0.00000100	
H	-5.01091200	1.68117900	-0.00000900	
H	-4.34352300	-2.58068300	0.00001000	
H	0.19691000	-1.72420100	0.00000900	
C	0.41717400	-0.65979000	0.00000400	
O	5.06583900	-2.15515200	-0.00000900	
H	4.58267900	-2.98738700	-0.00001100	
O	4.12293600	2.52892300	0.00000900	
H	5.08462900	2.53066200	0.00000200	
O	-6.15566300	-0.68166300	0.00000200	
O	-2.44821400	2.32353300	-0.00001000	
H	-3.19937300	2.92453700	-0.00001700	
**Name of cationic radical**				Energies at B3LYP/6-311G(d,p) in gas phase
C	4.61376500	0.15301600	-0.00000400	Zero-point correction= 0.231571 (Hartree/Particle)
C	4.13699500	-1.17171800	-0.00001600	Thermal correction to Energy= 0.247426
C	2.77320600	-1.41894300	-0.00001600	Thermal correction to Enthalpy= 0.248370
C	1.86029000	-0.33562200	-0.00000500	Thermal correction to Gibbs Free Energy= 0.187027
C	2.34793900	0.99281900	0.00000700	Sum of electronic and zero-point Energies= -841.339551
C	3.71772800	1.22847800	0.00000800	Sum of electronic and thermal Energies= -841.323696
H	5.68667600	0.31140700	-0.00000300	Sum of electronic and thermal Enthalpies= -841.322752
H	2.40004000	-2.43696400	-0.00002600	Sum of electronic and thermal Free Energies= -841.384095
H	1.68672400	1.84758700	0.00001500	
C	-0.56912500	0.28135100	0.00000400	
H	-0.31802600	1.33425000	0.00001400	
C	-1.95459500	-0.01267200	0.00000200	
C	-2.89668700	1.07799300	0.00001400	
C	-2.49463700	-1.33291000	-0.00001100	
C	-4.26388000	0.84486800	0.00001300	
C	-3.83894600	-1.56750200	-0.00001200	
H	-1.82022600	-2.17867300	-0.00002100	
C	-4.74017600	-0.46989100	0.00000000	
H	-4.95935000	1.67846100	0.00002300	
H	-4.24860000	-2.56881400	-0.00002200	
H	0.21808600	-1.69633700	-0.00001600	
C	0.46180800	-0.63914300	-0.00000600	
O	5.08209000	-2.13390400	-0.00002600	
H	4.68210600	-3.01121500	-0.00003400	
O	4.12250200	2.51697300	0.00001900	
H	5.08457900	2.57799600	0.00001900	
O	-6.03593500	-0.77710300	-0.00000200	

H -6.59563300 0.01062700 0.00000700<br>O -2.37749400 2.31429100 0.00002700<br>H -3.06778200 2.98888300 0.00003400	

Table S4: Cartesian coordinates and molecular enthalpies of the TS optimized at M05-2X/6-311++G(d,p) level in the studied environments.

**Name of TS**	**4**-O4'-H-$OOCH_3$			Energies (in the gas phase)
C	-5.52195800	0.37483600	0.14070000	Zero-point correction= 0.270233 (Hartree/Particle)
C	-4.57901900	1.37670500	-0.05544000	Thermal correction to Energy= 0.289504
C	-3.22151300	1.09064400	-0.09670200	Thermal correction to Enthalpy= 0.290448
C	-2.79182100	-0.22689500	0.05455900	Thermal correction to Gibbs Free Energy= 0.218379
C	-3.73283200	-1.24098700	0.26530200	Sum of electronic and zero-point Energies= -956.453782
C	-5.08563000	-0.93352000	0.30301000	Sum of electronic and thermal Energies= -956.434511
H	-6.58212600	0.58755900	0.17946300	Sum of electronic and thermal Enthalpies= -956.433567
H	-2.52947700	1.90902000	-0.22369300	Sum of electronic and thermal Free Energies= -956.505636
H	-3.40420500	-2.26596400	0.38775100	
C	-0.38107900	0.16474200	-0.46685100	
H	-0.62093000	1.14045700	-0.87300500	
C	1.02661200	-0.19374800	-0.52418000	
C	1.91209500	0.66364200	-1.19644500	
C	1.55159500	-1.36265400	0.07165900	
C	3.25512700	0.37527600	-1.29685800	
H	1.52406200	1.56793700	-1.64681500	
C	2.88784100	-1.65684600	-0.00759200	
H	0.89767100	-2.03120000	0.61327500	
C	3.77428700	-0.80152900	-0.70849600	
H	3.93829400	1.03589800	-1.81138000	
H	3.30220500	-2.54610500	0.44684800	
H	-1.15847800	-1.60478600	0.35458400	
C	-1.37731700	-0.60366300	0.00287900	
O	-4.94230400	2.67981200	-0.20709200	
H	-5.89603400	2.76298300	-0.15019800	
O	-6.04365200	-1.88123600	0.49836400	
H	-5.63357500	-2.74284500	0.59437400	
O	5.03847100	-1.11948500	-0.80714200	
H	5.68122600	-0.26195400	-0.42263600	
O	6.11568800	0.68278900	0.25761100	
O	4.97673100	1.24632500	0.73510400	
C	4.79736400	0.85423100	2.09535700	
H	3.84440800	1.27892800	2.40028600	
H	4.78010400	-0.23276900	2.15909600	
H	5.61487600	1.25579300	2.69187100	

Name of TS	4-O4'-H-OOCH$_3$	Energies (in the gas pentyl ethanoate)
C -5.47947700 0.09453300 0.31683700<br>C -4.84600700 1.29643300 0.02654600<br>C -3.48049200 1.34004800 -0.22465500<br>C -2.72598300 0.16204600 -0.18064900<br>C -3.35540200 -1.05195800 0.10159000<br>C -4.72131600 -1.07159300 0.34765900<br>H -6.54548600 0.08063100 0.50622500<br>H -2.99855400 2.28435100 -0.44873500<br>H -2.81404500 -1.98614700 0.12158600<br>C -0.38231300 -0.70718900 -0.24091800<br>H -0.70609800 -1.66095000 0.15911200<br>C 1.04621900 -0.60310700 -0.48853900<br>C 1.87214800 -1.66956000 -0.07874800<br>C 1.64891300 0.50712900 -1.11470200<br>C 3.23560100 -1.62753500 -0.25382300<br>H 1.41555700 -2.53128200 0.39153900<br>C 3.00751700 0.55556800 -1.30617300<br>H 1.04178500 1.33268400 -1.45814600<br>C 3.83432600 -0.51153900 -0.87682800<br>H 3.87132900 -2.44209500 0.06609700<br>H 3.47414800 1.40615100 -1.78502900<br>H -0.96090600 1.23756500 -0.79306600<br>C -1.28748600 0.26768600 -0.43716500<br>O -5.62142600 2.41348500 0.00082500<br>H -5.07885600 3.18086000 -0.20635600<br>O -5.28771900 -2.27908900 0.61564100<br>H -6.23310700 -2.17285000 0.76066900<br>O 5.13374200 -0.49025700 -1.08628800<br>H 5.57064500 0.35206700 -0.49779500<br>O 5.79330800 1.20229000 0.42716600<br>O 4.61317700 1.18372600 1.09303600<br>C 4.73993300 0.37312600 2.26613100<br>H 3.74170400 0.31372300 2.69321400<br>H 5.10600700 -0.61430400 1.98968100<br>H 5.43013600 0.85382500 2.95857900		Zero-point correction= 0.270525 (Hartree/Particle)<br>Thermal correction to Energy= 0.289596<br>Thermal correction to Enthalpy= 0.290540<br>Thermal correction to Gibbs Free Energy= 0.219669<br>Sum of electronic and zero-point Energies= -956.485498<br>Sum of electronic and thermal Energies= -956.466428<br>Sum of electronic and thermal Enthalpies= -956.465483<br>Sum of electronic and thermal Free Energies= -956.536355
**Name of TS**	**5**-O3'-H-OOCH$_3$	Energies (in pentyl ethanoate)
O -1.50155300 3.42145900 0.98439400<br>C -1.23470200 2.30680100 0.25256000<br>C 0.04655900 1.77920700 0.16102700		Zero-point correction= 0.450811 (Hartree/Particle)<br>Thermal correction to Energy= 0.482312<br>Thermal correction to Enthalpy= 0.483256

C	-2.30917100	1.72628000	-0.41751100	Thermal correction to Gibbs Free Energy= 0.383124
C	0.27546300	0.63827700	-0.61377300	Sum of electronic and zero-point Energies= -1642.444102
H	0.86286600	2.27574000	0.66997300	Sum of electronic and thermal Energies= -1642.412601
C	-2.07005600	0.59097600	-1.17605600	Sum of electronic and thermal Enthalpies= -1642.411657
H	-3.29569300	2.15872500	-0.35309700	Sum of electronic and thermal Free Energies= -1642.511789
C	-0.79532200	0.05019500	-1.29089300	
H	-0.64485300	-0.82106200	-1.91560600	
O	-3.07142800	-0.02797900	-1.89666500	
C	2.64593500	0.24685900	0.05722900	
H	2.53126500	0.91236600	0.90547800	
C	1.60214300	0.02363800	-0.75054900	
H	1.69064100	-0.68342900	-1.56738500	
C	3.97448300	-0.35917500	-0.06510700	
C	4.88978000	-0.17676600	0.95570900	
C	4.37145800	-1.11935800	-1.18830700	
C	6.18404700	-0.72910200	0.90749500	
H	4.63203200	0.40615500	1.83103600	
C	5.62989600	-1.67987300	-1.26577200	
H	3.68973900	-1.26496300	-2.01398300	
C	6.54629800	-1.49772400	-0.23089700	
O	7.78497900	-2.01933400	-0.25430500	
H	7.91064700	-2.52803300	-1.06387400	
C	-3.85287200	-1.25257900	0.06913600	
C	-5.13923900	-1.73982000	0.70651300	
C	-6.06861600	-0.56651900	0.92822500	
C	-6.31216100	0.15277000	-0.39408100	
C	-4.20108800	-0.49750800	-1.21146600	
H	-5.62293100	-2.45832700	0.03468000	
H	-3.34396200	-0.58123900	0.76515600	
H	-6.78610700	-0.53512200	-1.10399100	
H	-4.69668600	-1.16965200	-1.91600200	
H	-5.59834100	0.13421400	1.62652900	
O	-5.05352400	0.58975700	-0.90624800	
H	-8.56032500	0.39682200	0.70502900	
C	-7.18757100	1.38562700	-0.23615700	
H	-7.14785000	1.96109800	-1.15885000	
H	-6.79830900	2.00526100	0.57801200	
O	-8.54424700	1.03763200	-0.01454100	
O	-7.32172200	-1.00110000	1.44122900	
H	-7.15056500	-1.54819000	2.21532100	

O	-4.90335100	-2.33794800	1.97065200	
H	-4.28991900	-3.06851500	1.84222500	
O	-3.06056900	-2.39653400	-0.20360000	
H	-2.15256000	-2.11488800	-0.35281600	
H	-0.69028600	3.75456900	1.38103400	
H	5.91554800	-2.25808100	-2.13653500	
O	7.03869400	-0.56694500	1.89259700	
H	7.45837800	0.47518300	1.80823900	
O	7.83330000	1.59287400	1.36035900	
O	7.40545700	1.52793600	0.07727400	
C	8.50140100	1.17176200	-0.77160200	
H	8.92878600	0.22849100	-0.43405700	
H	8.08175100	1.07680400	-1.77057000	
H	9.24845800	1.96372200	-0.73947800	
**Name of TS**	**5**-O4'-H-OOCH$_3$			Energies (in pentyl ethanoate)
O	-1.68657400	3.26563600	1.52515700	Zero-point correction= 0.451525 (Hartree/Particle)
C	-1.34245100	2.24963000	0.68983600	Thermal correction to Energy= 0.482718
C	-0.04560800	1.75925700	0.62493600	Thermal correction to Enthalpy= 0.483662
C	-2.35661400	1.73271900	-0.11369900	Thermal correction to Gibbs Free Energy= 0.385863
C	0.25992000	0.72032300	-0.26089600	Sum of electronic and zero-point Energies= -1642.452638
H	0.72031900	2.20735700	1.24529900	Sum of electronic and thermal Energies= -1642.421446
C	-2.04394400	0.69555100	-0.97841400	Sum of electronic and thermal Enthalpies= -1642.420502
H	-3.35450100	2.14023200	-0.06634400	Sum of electronic and thermal Free Energies= -1642.518301
C	-0.75154400	0.19278100	-1.06745700	
H	-0.53900400	-0.59892100	-1.77448000	
O	-2.97949400	0.14884500	-1.83183700	
C	2.62956500	0.36143600	0.44842600	
H	2.48355800	0.98579300	1.32210100	
C	1.60448500	0.15089200	-0.39166500	
H	1.73249600	-0.50705400	-1.24282600	
C	3.96973200	-0.20089100	0.31731000	
C	4.88314400	0.03912000	1.34872200	
C	4.37496600	-0.96715400	-0.79999700	
C	6.16647800	-0.46953800	1.27375900	
H	4.59478200	0.61788900	2.21632600	
C	5.64856300	-1.47289800	-0.87879400	
H	3.68530500	-1.15281700	-1.60994300	
C	6.57574700	-1.22804300	0.15055800	
O	7.81585500	-1.69555900	0.14948500	
H	8.40582100	-1.09760700	-0.56762800	

C	-3.90119200	-1.28142400	-0.07803600	
C	-5.22952600	-1.83034300	0.40399100	
C	-6.17506200	-0.68326300	0.68578000	
C	-6.32544600	0.17800700	-0.56329800	
C	-4.15642100	-0.38847300	-1.28989300	
H	-5.65915400	-2.46727700	-0.37785900	
H	-3.44820300	-0.69386500	0.72431600	
H	-6.74685800	-0.42421300	-1.37649400	
H	-4.59338200	-0.97770100	-2.09962000	
H	-5.75625200	-0.06558900	1.48767500	
O	-5.03433000	0.66207000	-0.93340400	
H	-8.64856200	0.31260500	0.39031200	
C	-7.21229600	1.39105500	-0.33220700	
H	-7.10628400	2.06615900	-1.17904200	
H	-6.88487100	1.91289400	0.57271600	
O	-8.58084000	1.02907600	-0.25059600	
O	-7.46056900	-1.16324200	1.05820100	
H	-7.34369600	-1.79481000	1.77604700	
O	-5.08593400	-2.56575100	1.60791300	
H	-4.46817200	-3.28533400	1.44329900	
O	-3.08661000	-2.39149800	-0.41592800	
H	-2.17139600	-2.09950900	-0.47130300	
H	-0.91398900	3.56250800	2.01642600	
H	5.97238200	-2.05170700	-1.73349200	
O	7.05468300	-0.24512300	2.25932300	
H	7.85768600	-0.74063500	2.03806300	
O	8.84048600	-0.10268600	-1.27986100	
O	7.76650600	0.71274100	-1.25957600	
C	8.01097300	1.82447400	-0.38912000	
H	8.82698800	2.41938600	-0.79634300	
H	7.08485800	2.39338000	-0.37413500	
H	8.26388500	1.46346200	0.60685600	
**Name of TS**	**11**-O4'-H-OOCH$_3$			Energies (in the gas phase)
C	5.67992200	-0.22161400	0.21213700	Zero-point correction= 0.275741 (Hartree/Particle)
C	4.80434000	-1.29892500	0.15239500	Thermal correction to Energy= 0.295790
C	3.43255700	-1.10797100	0.05910100	Thermal correction to Enthalpy= 0.296735
C	2.92090400	0.18760900	0.01615900	Thermal correction to Gibbs Free Energy= 0.223886
C	3.79283700	1.27918400	0.08931100	Sum of electronic and zero-point Energies= -1031.699964
C	5.16117600	1.06628800	0.18179700	Sum of electronic and thermal Energies= -1031.679915
H	6.75036400	-0.35889600	0.28993000	Sum of electronic and thermal Enthalpies= -1031.678971

H 2.79270500 -1.97712900 0.04804900<br>H 3.39922400 2.28807600 0.06141000<br>C 0.56015600 -0.41882300 -0.51667000<br>H 0.87594900 -1.40858300 -0.82468600<br>C -0.86958600 -0.16004000 -0.64198700<br>C -1.65776700 -1.11142300 -1.32303000<br>C -1.48075200 0.98565200 -0.11744800<br>C -3.00939900 -0.92611500 -1.49543600<br>H -1.18054400 -1.99776500 -1.71846900<br>C -2.83475300 1.17751900 -0.28833700<br>H -0.92303800 1.72737500 0.43523900<br>C -3.62871500 0.22224600 -0.97798800<br>H -3.62365500 -1.65097900 -2.01079000<br>H 1.18788000 1.47378600 0.13493500<br>C 1.48547600 0.45939800 -0.10368700<br>O 5.24971900 -2.58483400 0.19260300<br>H 6.20521000 -2.59807800 0.27393100<br>O 6.05566700 2.09095100 0.24769700<br>H 5.59179000 2.92969400 0.21402400<br>O -4.91662700 0.48915400 -1.10222500<br>H -5.48083300 -0.16754600 -0.39352200<br>O -5.77451700 -0.84479800 0.64641500<br>O -4.54751600 -1.16050000 1.11022500<br>C -4.27869800 -0.42173900 2.30461800<br>H -3.27099500 -0.70297400 2.59804100<br>H -4.33928500 0.64546600 2.09550200<br>H -5.00489300 -0.70570700 3.06372000<br>O -3.45122400 2.26160600 0.21939500<br>H -4.37007000 2.22314200 -0.08581100	Sum of electronic and thermal Free Energies= -1031.751819
**Name of TS** **11**-O3’-H-OOCH$_3$	Energies (in pentyl ethanoate)
C 5.58259800 0.09662900 0.24524500<br>C 4.95491400 1.18289400 -0.35054300<br>C 3.57748000 1.19952700 -0.53273800<br>C 2.80589800 0.10951500 -0.11710300<br>C 3.42624300 -0.98237300 0.49214300<br>C 4.80399800 -0.97762300 0.66284100<br>H 6.65617700 0.10418500 0.38361500<br>H 3.10072100 2.05291000 -1.00045400<br>H 2.86401000 -1.83147300 0.85198400<br>C 0.49954900 -0.84620200 -0.22612400	Zero-point correction= 0.274873 (Hartree/Particle)<br>Thermal correction to Energy= 0.295000<br>Thermal correction to Enthalpy= 0.295944<br>Thermal correction to Gibbs Free Energy= 0.222805<br>Sum of electronic and zero-point Energies= -1031.722250<br>Sum of electronic and thermal Energies= -1031.702124<br>Sum of electronic and thermal Enthalpies= -1031.701180<br>Sum of electronic and thermal Free Energies= -1031.774319

H 0.86950900 -1.82829400 0.04579200	
C -0.94616500 -0.77962900 -0.46251200	
C -1.66293100 -1.99133000 -0.55778700	
C -1.64901200 0.40611600 -0.59338700	
C -3.02435300 -2.01459600 -0.79757000	
H -1.12926000 -2.92643000 -0.44771700	
C -3.03405600 0.42131600 -0.83358800	
H -1.16311300 1.36769900 -0.49847200	
C -3.72281400 -0.82067600 -0.93945400	
H -3.55185300 -2.95755700 -0.87618900	
H 0.99313000 1.14215200 -0.67423400	
C 1.35717500 0.17372700 -0.34985900	
O 5.74775900 2.21693200 -0.74233300	
O 5.36160300 -2.06523000 1.26211800	
O -5.04617100 -0.77055500 -1.17330100	
H -5.40016700 -1.66525600 -1.23680800	
H 6.31329700 -1.94476100 1.33688100	
H 5.20632700 2.90894400 -1.13489400	
O -3.69305600 1.54924800 -0.97708600	
H -3.85421200 1.98267900 0.05393000	
O -3.99592900 2.11774600 1.29478800	
O -3.74684300 0.86146200 1.73568200	
C -4.98621300 0.19490100 1.99572500	
H -5.59697700 0.20021300 1.09388900	
H -4.71977600 -0.81981200 2.28285900	
H -5.50057600 0.70480500 2.80914600	
**Name of TS** **11**-O4'-H-OOCH$_3$	Energies (in pentyl ethanoate)
C 5.68350500 -0.13073800 0.26421900	Zero-point correction= 0.275532 (Hartree/Particle)
C 4.85108100 -1.24362800 0.20412600	Thermal correction to Energy= 0.295476
C 3.47668100 -1.11215400 0.05931600	Thermal correction to Enthalpy= 0.296420
C 2.91600200 0.16301900 -0.03363300	Thermal correction to Gibbs Free Energy= 0.224285
C 3.74278700 1.28988700 0.03396000	Sum of electronic and zero-point Energies= -1031.731481
C 5.11547000 1.13392100 0.17970300	Sum of electronic and thermal Energies= -1031.711537
H 6.75478800 -0.23399100 0.38129700	Sum of electronic and thermal Enthalpies= -1031.710593
H 2.87222700 -2.00692000 0.03594400	Sum of electronic and thermal Free Energies= -1031.782728
H 3.31318400 2.28215000 -0.03401400	
C 0.55974500 -0.55176000 -0.47439100	
H 0.87286500 -1.58043300 -0.60850800	
C -0.87200000 -0.32588600 -0.63710500	
C -1.66951200 -1.42387500 -1.02612700	

C -1.47842900 0.91969400 -0.42647400<br>C -3.02732200 -1.29159200 -1.20210300<br>H -1.19507100 -2.38286600 -1.18623700<br>C -2.83869200 1.05727900 -0.60251800<br>H -0.90986900 1.78679100 -0.12196600<br>C -3.64251700 -0.04917600 -0.98465200<br>H -3.64508100 -2.13013300 -1.49406300<br>H 1.17056300 1.42421400 -0.10551000<br>C 1.47695000 0.38918600 -0.20040500<br>O 5.35036400 -2.50610400 0.29025400<br>H 6.30628100 -2.47379300 0.39597400<br>O 5.95996400 2.19790300 0.24720100<br>H 5.45811100 3.01581800 0.17446500<br>O -4.94000600 0.16985000 -1.14407200<br>H -5.47244900 -0.28045600 -0.28825700<br>O -5.75405100 -0.69920500 0.90882100<br>O -4.51695100 -0.82561200 1.43030300<br>C -4.26558400 0.23452300 2.36127600<br>H -3.23136100 0.11157100 2.67270000<br>H -4.41528900 1.19611300 1.87242800<br>H -4.94288500 0.12921600 3.20752000<br>O -3.44071900 2.24508100 -0.40175100<br>H -4.37382800 2.13523500 -0.63898200	
